# Pursuing the Red-Shift in Glycoporphyrins—A New Synthetic Approach toward Glycoconjugates from the *meso*-Tetraethynylporphyrin Scaffold

**DOI:** 10.1021/acsorginorgau.6c00033

**Published:** 2026-06-25

**Authors:** Jakub Mitrzak, Maciej Malinowski

**Affiliations:** 49566 Warsaw University of Technology, Faculty of Chemistry, ul. Noakowskiego 3, Warsaw 00-664, Poland

**Keywords:** sonogashira reaction, glycals, porphyrin conjugate, glycoporphyrin, photosensitizer(s); ROS, palladium catalysis

## Abstract

Photodynamic therapy is a promising approach to anticancer treatment. For further development of this technique, new photosensitizers that better meet biological criteria are highly desired. Glycoporphyrins are considered more than favorable synthetic targets in this matter. This is due to their increased solubility in aqueous solutions and their targeting ability toward cancer cells. Herein, we report synthetic studies, and we propose an original Sonogashira-based strategy toward unique porphyrin-glycal conjugates. The proposed methodology allowed us to synthesize 5 new examples of photosensitizers in which the sugar unit influenced another crucial factor—the bathochromic shift of the photosensitizer’s absorption profile. These structures were able to absorb wavelengths from the region of 740–780 nm. The studies present the development of a synthetic strategy toward water-soluble glycoporphyrins and the initial recognition of their singlet oxygen production ability.

## Graphical abstract

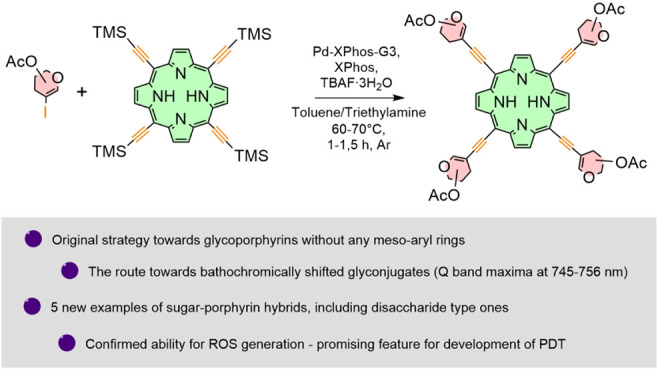

## Introduction

Among the family of organic dyes, the porphyrin framework has become one of the most widely explored scaffolds for its biomedical applications, including bioimaging and photoguided therapies like photodynamic inactivation (PDI) and photodynamic therapy (PDT).^1–7^ The latter is a particularly promising modern approach in anticancer treatment, as it exploits a nontoxic agent (photosensitizer, PS) that, upon light irradiation, produces reactive oxygen species (ROS), which are locally toxic to adjacent cells. As a result, if PS accumulates near cancer cells, their eradication might be achieved with minimal side effects. Regarding the typical properties of porphyrins, they are treated as especially promising scaffolds for the design of new PSs.^3–9^ They are considered generally biocompatible frameworks, probably due to the omnipresence of this architecture in nature (porphyrin is a framework present in hemes and some chlorophylls). Second, the optical features of these macroheterocycles (absorption of light from the visible region and efficient generation of ROS) naturally predestine them for exploitation in PDT.

Nevertheless, there are still several aspects to be addressed regarding the general application of the porphyrin scaffold in PDT, requiring its functional modifications.^10,11^ First of all, typical synthetic porphyrins are characterized by low solubility in polar solvents, which significantly reduces their bioavailability. Moreover, it would be of high value if porphyrin were capable of being vectorized toward cancer cells; thus, the selectivity of the whole therapy might be improved. Finally, a bathochromic shift of the absorption maxima would allow us to use lower-energy and more penetrating light doses.

Considering the aforementioned criteria, glycoporphyrins (porphyrin-carbohydrate conjugates) are recognized as prospective candidates for PSs of the third generation. The natural hydrophilic character of sugar moieties provides the amphiphilic nature for the whole system,^12^ improving their solubility in polar solvents and bioavailability.^13–18^ Furthermore, the carbohydrate-recognizing lectins are usually overexpressed in cancer cells, so the sugar unit might play the role of a Trojan horse, providing an improvement in selectivity toward phototherapy targets.^19–27^

From the perspective of synthetic studies, glycoporphyrins remain an interesting target, as typical routes toward them usually lead to potentially hydrolyzable C–O or ester bonds, and the literature lacks a more general approach toward these types of frameworks.^28–30^ Apart from that, among different strategies, click chemistry has offered the concept most suited to be called versatile, and to date, many new examples of carbohydrate-porphyrin hybrids have been synthesized following it.^31–40^

In the past few years, our group has introduced an alternative idea in synthetic studies, and we initiated the use of palladium-catalyzed strategies toward C–C bonded glycoconjugates based on the Suzuki-Miyaura reaction^41^ or Sonogashira approach.^42–44^ The main challenge of the catalytic system is to adjust it to efficiently cross-couple two subunits (porphyrin macroheterocycle in reaction with carbohydrate residue) that are so different in terms of chemical origins and chemical properties. In the case of Sonogashira reactivity, despite two theoretical approaches, we have already discovered that the electrophilic partner (halogen atom) should be localized at the macrocycle, while the ethynyl substituent should be a part of the sugar moiety (Scheme 1A). During these studies, we also found that if the ethynyl fragment is linked to the porphyrin starting material, the Glaser-type side reactivity prevails, leading to undesired oligomerization (Scheme 1B), and glycoporphyrins were not successfully obtained.^42^ Nevertheless, such dendrimerization might be used to its advantage in the construction of glucose colorimetric sensors.^45^

**Scheme 1:**
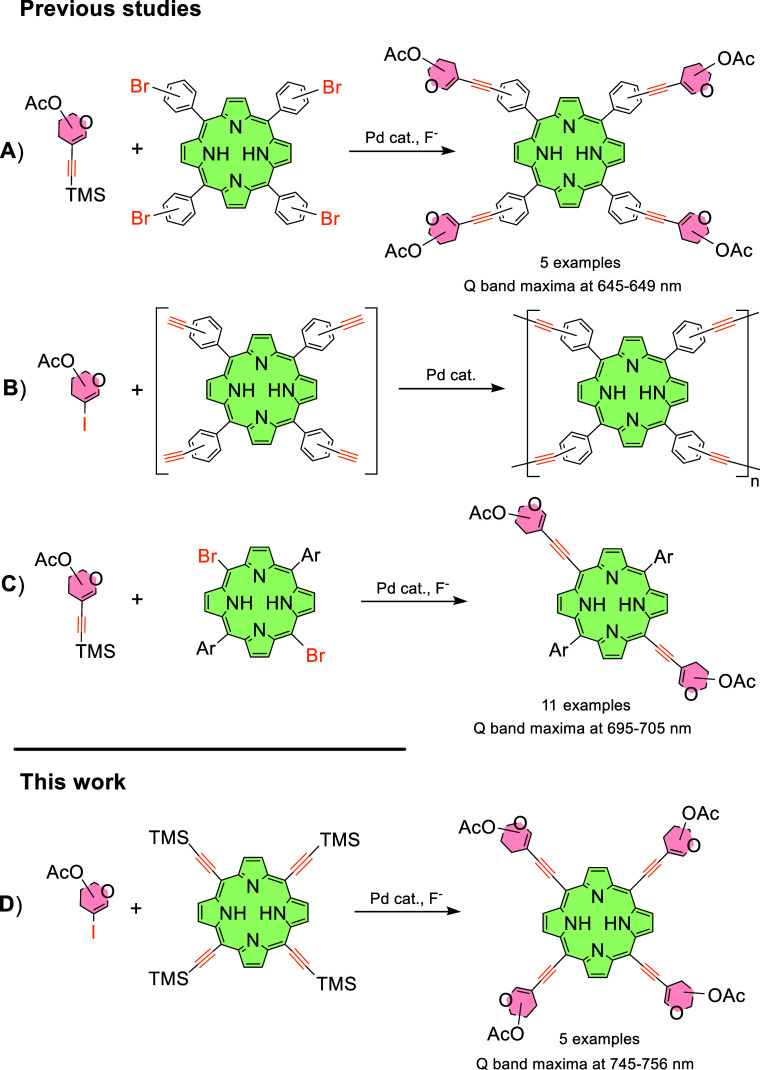
State of Art on Sonogashira-type Reactivity towards Glycoporphyrins

In addition to other glycal-porphyrin hybrids, we already demonstrated on porphyrins of A_2_B_2_-type that if cross-coupling occurs directly at the *meso*-position, the sugar residue might participate in the conjugated system, and the red shift (up to 20 nm) of the porphyrin absorption maxima is observed (Scheme 1C). Therefore, the sugar unit might play an additional beneficial role in the whole photosensitizing system,^43^ and this area has not yet been explored in any other glycoporphyrin studies.

Considering these results, we concluded that the full advantage of this phenomenon might be pursued if successful conjugation could be achieved through all of the *meso* positions on A_4_-type porphin derivatives. Such red-shifts of Q bands have been reported in some porphin conjugates,^46–48^ and in the case of porphin derivatives, Sonogashira procedures were introduced with the use of a triphenylarsine ligand to obtain final products in a range of modest to good yields.^47–51^ Additionally, the Sonogashira-type reactivity was widely applied in the synthesis of molecules for biomedical applications^52^ and is generally recognized as a reliable method of porphyrin framework functionalization.^53^

Herein, we present our studies on the synthesis of red-shifted glycoporphyrins from the *meso*-tetraethynylporphyrin scaffold (Scheme 1D). Compared with previously described glycoconjugates,^42–44^ structures presented herein are characterized by the lack of *meso*-aromatic substituents; therefore, their hydrophobicity is significantly reduced. Following the new method, we obtained 5 original examples of glycoconjugates featuring the bathochromic shift of absorption maxima of up to 40–50 nm.

## Results and Discussion

### Initial Attempts—Application of Glycosylated Aldehyde

Regarding the final glycoporphyrin target of our studies, we began with the recognition of the most typical strategy for the synthesis of macroheterocyclic scaffolds. We decided to test the possibility of obtaining propargyl-type glycosylated aldehyde and to use it in the formation of the porphyrin core under Lindsey conditions (Scheme 2).^54^ That is, this strategy has been somewhat recognized in the case of some carbohydrate-porphyrin hybrids;^55–57^ however, it has not yet been explored with glycal residues.

**Scheme 2:**
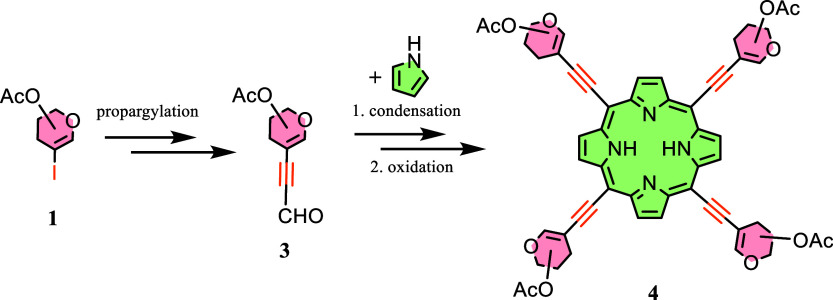
Concept of Strategy Exploiting the Glycosylation Prior to Porphyrin Core Formation

The introduction of a direct procedure toward 2-iodoglycals^58^ has attracted much attention toward them as biologically interesting scaffolds for metal-catalyzed transformations.^59,60^ With the microwave-assisted improvement in this process, gram-scale amounts of 2-iodoglycals are easily accessible, and we decided to exploit this protocol to our advantage in these studies.^61^ As a model glycal substrate, we chose to use 2-iodo-d-glucal derivative **1a** as the most convenient in terms of economical availability. Its palladium-catalyzed reactivity has been widely recognized in modern studies,^62–68^ including methodology of the Sonogashira reaction.^42,69,70^ Following our previously reported protocol, we introduced a propargyl alcohol moiety, leading to alkyne **2a** in a very good yield of 80% (Scheme 3). The subsequent oxidation step was tested under 3 typical conditions described for propargyl alcohol moieties. Among PCC, MnO_2_, or Dess-Martin reagents, the last one was the most optimal to obtain aldehyde **3a** (Scheme 3).

**Scheme 3:**
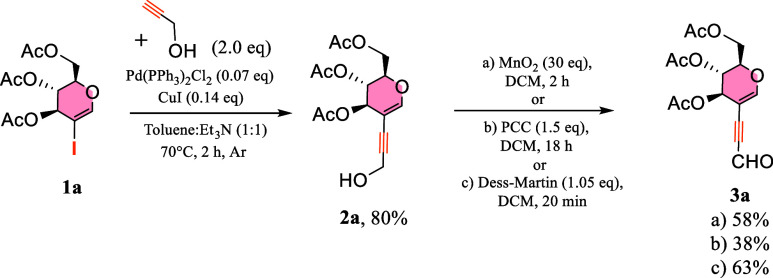
Synthesis of Glycosylated Propargyl Aldehyde **3a**

With aldehyde **3a** in hand, we screened potential catalyst conditions in Lindsey-type procedures (Scheme 4). We quickly excluded BF_3_•EtO_2_ from potential catalysts, as even the smallest amounts of it activated side oligomerization reactivity at the glycal moiety. We then focused on an alternative reagent (TFA). However, despite several attempts, we concluded that aldehyde **3a** was not an adequate substrate to synthesize porphyrin **4a**. Our experiments focused on different concentrations of TFA, but we did not see positive results in the range of 1.7–15.2 mmol/L (Scheme 4). The lower concentration did not activate the aldehyde function for nucleophilic attack, and the starting material was recovered. At higher concentrations of TFA, side-reactivity occurred, leading to quick blackening of the reaction mixture without detecting even traces of porphyrin **4a**.

**Scheme 4:**
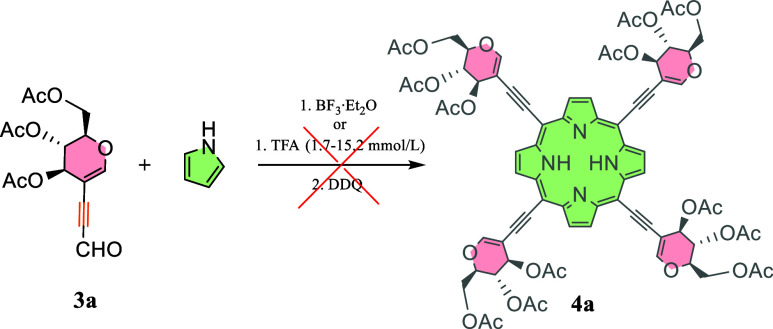
Attempts on Macrocyclization with the use of Aldehyde **3a**

### Glycosylation of the Porphyrin Framework

Following the aforementioned results, we alternated the strategy. We decided to synthesize first the macroheterocycle core armed with conjugable, reactive groups and then to glycosylate this porphyrin scaffold. This might be achieved from two variant approaches (Scheme 5A,B). In both strategies, two glycal starting materials have been well recognized in metal-catalyzed methodologies, and they are rather easily accessible. The major difference is in the availability of the porphyrin scaffold (**5a** vs **5b**, Scheme 5), in which alkynylated porphin **5a** is directly synthesized from commercially available aldehyde (for the synthesis see SI) unlike tetrabrominated porphin **5b** that is obtained through a multistep process with rather low yields. Considering this, we decided to explore only route A in our studies, keeping in mind that suppression of Glaser-type side reactions leading to oligomerization of the macrocycle framework would be the main challenge, as even a singular side process would prevent us from obtaining the final product.

**Scheme 5:**
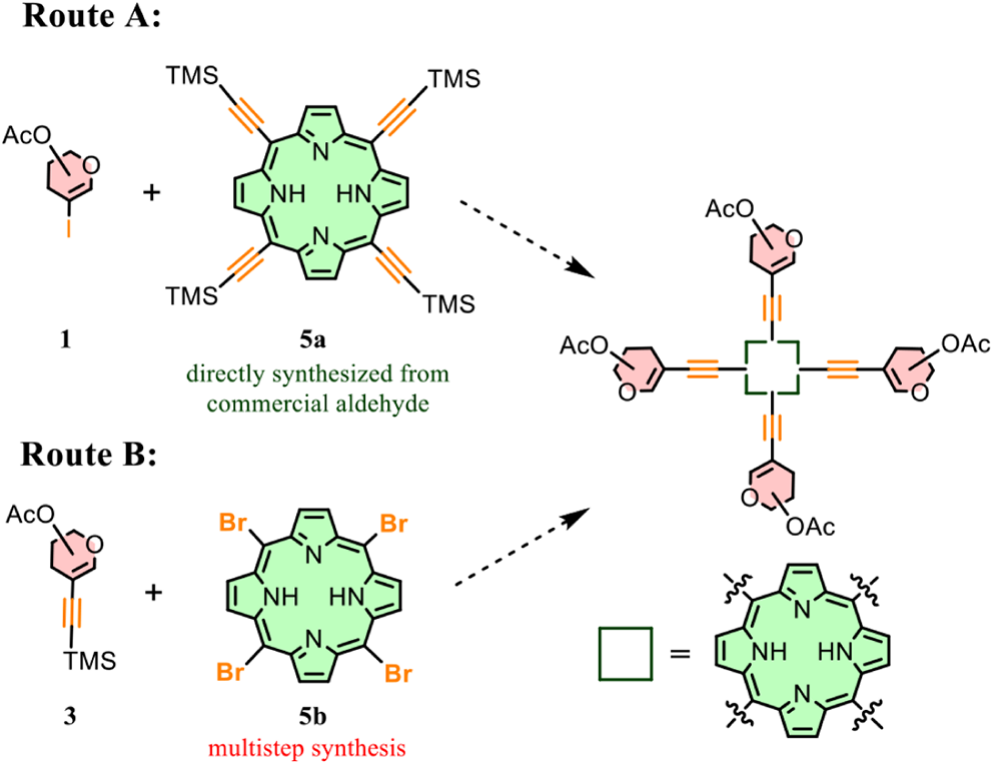
Theoretical Approaches to the Same Type of Glycoporphyrins **4** *via* Sonogashira Reaction

To pursue the goal of our studies, we decided to optimize the Sonogashira procedure between 2-iodoglycal **1a** and porphyrin **5a** (Scheme 6). Starting conditions were selected based on knowledge from previous studies conducted in our group. As the most convenient catalyst, we chose Pd-XPhos-G3, while for the mixture of solvents adequate for both subunits, we decided to use toluene and triethylamine. Finally, we had already observed that oxidative addition of palladium most efficiently occurs with 2-iodoglycals at a temperature of at least 60 °C, so this parameter was kept in current studies as well.^42,66^

**Scheme 6:**
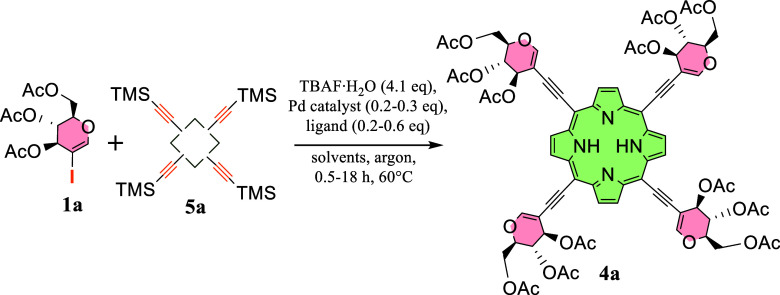
Model Reaction in Optimization of Sonogashira Approach towards Glycoporphyrin **4a**

In very initial experiments toward **4a**, we discovered major challenges to overcome in the whole strategy. First of all, the main side-reactivity of porphyrin **5a** was its oligomerization, which occurred simultaneously under reaction conditions. Second, we also observed that the highly active catalyst produced from the Buchwald palladium third-generation precatalyst efficiently catalyzes the desired Sonogashira reaction; however, after successful cross-coupling, it is able to launch an unwanted dealkynylation process as well. Thus, some sort of prevention should be applied in our procedure. And last but not least, the overall yield of obtaining **4a** describes the yield of 4 subsequent Sonogashira transformations, so every step must be maximally efficient, as even a single unsuccessful reaction prevents from obtaining the final glycoconjugate **4a**.

Table 1 describes our route to optimal Sonogashira conditions, leading to **4a**. In the starting experiment (Table 1, entry 1), under conditions inspired from previous studies, we were able to isolate the product **4a** in 3% yield, and we also observed significant amounts of insoluble porphyrin oligomers. Interestingly, prolonging the reaction time did not improve the yield (Table 1, entry 1 vs entry 2); on the contrary, the dealkinylation process prevailed and no glycoconjugate **4a** was isolated. This prompted us to test shorter reaction times (Table 1, entry 1 vs entries 3, 4). Indeed, some improvement was achieved by following this concept. We observed that Sonogashira processes were not fast enough to finish the reaction after 0.5 h (Table 1, entry 3), although a 2 h reaction was quite a compromise to see high conversion of porphyrin starting material with relatively low side-reactivity, leading to **4a** in 13% yield (Table 1, entry 4). Some enhancement of product formation (Table 1, entry 5) was also observed with the increase of catalyst loading to 0.3 eq (corresponding to 0.075 eq per reaction center), so this parameter was maintained in further experiments. The screening of the solvent mixture (Table 1, entry 5 vs entries 6, 7) suggested that toluene is a rather adequate choice in this process. With the higher catalyst loading, we determined that a 1 h reaction is more optimal, and **4a** could be isolated with 17% yield (Table 1, entry 7). Some progress was made when a decrease in catalyst activity was applied by using more equivalents of stabilizing ligand (Table 1, entry 8). This adjustment allowed us to obtain glycoporphyrin in 21% yield. It also encouraged us to test other catalytic systems (Table 1, entries 10–13); however, none of the experiments improved the final yields. Probably, harsher conditions are required for efficient oxidative addition of palladium when Pd­(OAc)_2_ is used in this transformation (Table 1, entry 10), although not much oligomerization was observed under these conditions. Unlike in the experiment using CuI as a cocatalyst (Table 1, entry 11), the Glaser-like reactivity was momentous, which prevented us from obtaining any amounts of **4a**. This clearly showed us that any amounts of copper are to be excluded from the protocol. Another catalytic system, described for *meso*-ethynylporphyrin-type starting material, was also not efficient in this transformation (Table 1, entry 12). An alternative third-generation precatalyst (Pd-BrettPhos-G3, Table 1, entry 13) showed some positive reactivity (5% yield); however, it was not superior to Pd-XPhos-G3.

**Table 1:** Optimisation Studies on the Synthesis of Glycoconjugate **4a** ^a^

Entry	Catalyst (eq)	Ligand	Solvents (1/1; v/v)	Reaction time [h]	Porphyrin dosing	Overall yield^b^
1.	Pd-XPhos-G3 (0.2)	XPhos (0.2)	Toluene/triethylamine	3	-	3%
2.	Pd-XPhos-G3 (0.2)	XPhos (0.2)	Toluene/triethylamine	18	-	0%
3.	Pd-XPhos-G3 (0.2)	XPhos (0.2)	Toluene/triethylamine	0.5	-	5%
4.	Pd-XPhos-G3 (0.2)	XPhos (0.2)	Toluene/triethylamine	2	-	13%
5.	Pd-XPhos-G3 (0.3)	XPhos (0.3)	Toluene/triethylamine	2	-	16%
6.	Pd-XPhos-G3 (0.3)	XPhos (0.3)	Acetonitrile/triethylamine	2	-	12%
7.	Pd-XPhos-G3 (0.3)	XPhos (0.3)	1,4-Dioxane/triethylamine	2	-	10%
8.	Pd-XPhos-G3 (0.3)	XPhos (0.3)	Toluene/triethylamine	1	-	17%
9.	Pd-XPhos-G3 (0.3)	XPhos (0.6)	Toluene/triethylamine	1	-	20%
10.	Pd(OAc)_2_ (0.3)	XPhos (0.6)	Toluene/triethylamine	18	-	0%
11.	Pd(OAc)_2_ (0.2), CuI (0.4 equiv)	BrettPhos (0.2)	Toluene/triethylamine	1	-	0%
12.	Pd_2_(dba)_3_ (0.15)	AsPh_3_ (1.6)	Toluene/triethylamine	3	-	0%
13.	Pd-BrettPhos-G3 (0.3)	BrettPhos (0.6)	Toluene/triethylamine	1	-	5%
14.	Pd-XPhos-G3 (0.3)	XPhos (0.6)	Toluene/triethylamine	1		12%^c^
15.	Pd-XPhos-G3 (0.3)	XPhos (0.6)	Toluene/triethylamine	1	**5a** Solid portionwise addition (4 portions, 5 min interval)	26%
16.	Pd-XPhos-G3 (0.3)	XPhos (0.6)	Toluene/triethylamine	1	**5a** Portionwise injection (14 portions, 3 min interval)	33%
17.	Pd-XPhos-G3 (0.3)	XPhos (0.6)	Toluene/triethylamine	1	**5a** Continuous injection (20 min)	41%
**18.**	**Pd-XPhos-G**3 (0.3)	**XPhos (0.6)**	Toluene/triethylamine	**1**	**5a Continuous injection (20 min), 5-fold scale reaction** ^d^	**44%**

a General conditions: porphyrin **5a** (20 mg, 0.029 mmol, 1.0 equiv), 2-iodoglycal **1a** (69 mg, 8.0 equiv), catalyst (0.2–0.3 equiv), ligand (0.2–0.6 equiv), and TBAF·3H_2_O (37.5 mg, 4.1 equiv), solvent 1 + triethylamine (6 + 6 mL) under an argon atmosphere at 60 °C.

b Final yield of 4 subsequent reactions, isolated yield of **4a** after chromatographic purification.

c The volume of the solvents increased to 24 mL.

d Reaction at 70 °C.

At this stage of the optimization, we concluded that the main obstacle in the process was the oligomerization of the porphyrin substrate, and it needs to be suppressed to pursue the studies. Following this concept, we attempted to dilute the reaction mixture; however, it slowed down all of the transformations without increasing the amount of product (Table 1, entry 9 vs entry 14). Some progress was made after we decided not to add porphyrin alkyne **5a** in one portion but to divide it into smaller ones (portionwise addition of solid). By this approach, we kept the high concentration of other reagents while keeping the porphyrin substrate at a lower concentration. This allowed us to increase the yield to 26% (Table 1, entry 15). Nevertheless, the level of inert atmosphere remained uncertain due to the need for regular opening of the reaction vessel. We improved this approach by using syringe injection of the **5a** solution (14 portions) into the reaction mixture. By this strategy, we isolated glycoconjugate **4a** in 33% yield (Table 1, entry 16). However, in this experiment, we observed that if the substrate solution was transferred at room temperature, the solubility of **5a** was limited, and significant amounts of porphyrin always remained in the syringe. This prompted us to make a final modification of the reaction protocol. We decided to solubilize **5a** at 80 °C and to apply continuous addition of this warm solution through a cannula. This allowed the yield of **4a** to improve to 41% (Table 1, entry 17). Our final experiment was dedicated to increasing the scale of the reaction (5-fold scale). We observed that if the reaction temperature was raised to 70 °C, we were able to better control the proper rate of substrate addition, and this temperature was applied in the final experiment (Table 1, entry 18). Following this protocol, we isolated the glycoconjugate **4a** in a 44% yield, which corresponds to an 81% yield of each Sonogashira step in the whole process. The experiment also confirmed very good scalability of this strategy. These conditions were treated as the optimal ones in further studies.

With optimal conditions in hand, we decided to determine the scope of our methodology. Unlike in our previous studies, the Sonogashira strategy developed herein allows us to apply 2-iodoglycals as sugar synthons. This is particularly convenient, as new routes toward 2-haloglycals are regularly proposed in the current literature and provide access to direct glycosylation protocols of macrocycles. From the perspective of future biological studies, the carbohydrate moieties are important subunits responsible for targeting ability toward cancer cells; thus, diversification of them helps in the adjustment of the PDT selectivity. Lastly, in our previous studies, we already reported that d-galactal residue most significantly influences the red-shifted absorption profile of the glycohybrid,^43^ so the sugar unit itself might provide benefits for the photophysical properties of the molecules.

During the scope studies (Scheme 7) we exemplified the glycohybrids using monosaccharide glycals of d- and l-series in nearly the same yield: d-galactal moiety (Scheme 7, **4b**, 34%), d-xylal (**4c**, 37%), and l-rhamnal residue (**4d**, 40%). For the first time, we were also able to introduce more than one disaccharide-type glycal to glycoporphyrins (d-maltal motif, **4e**, 22%), proving the complementary character of this strategy in comparison to our previous methodologies.

**Scheme 7:**
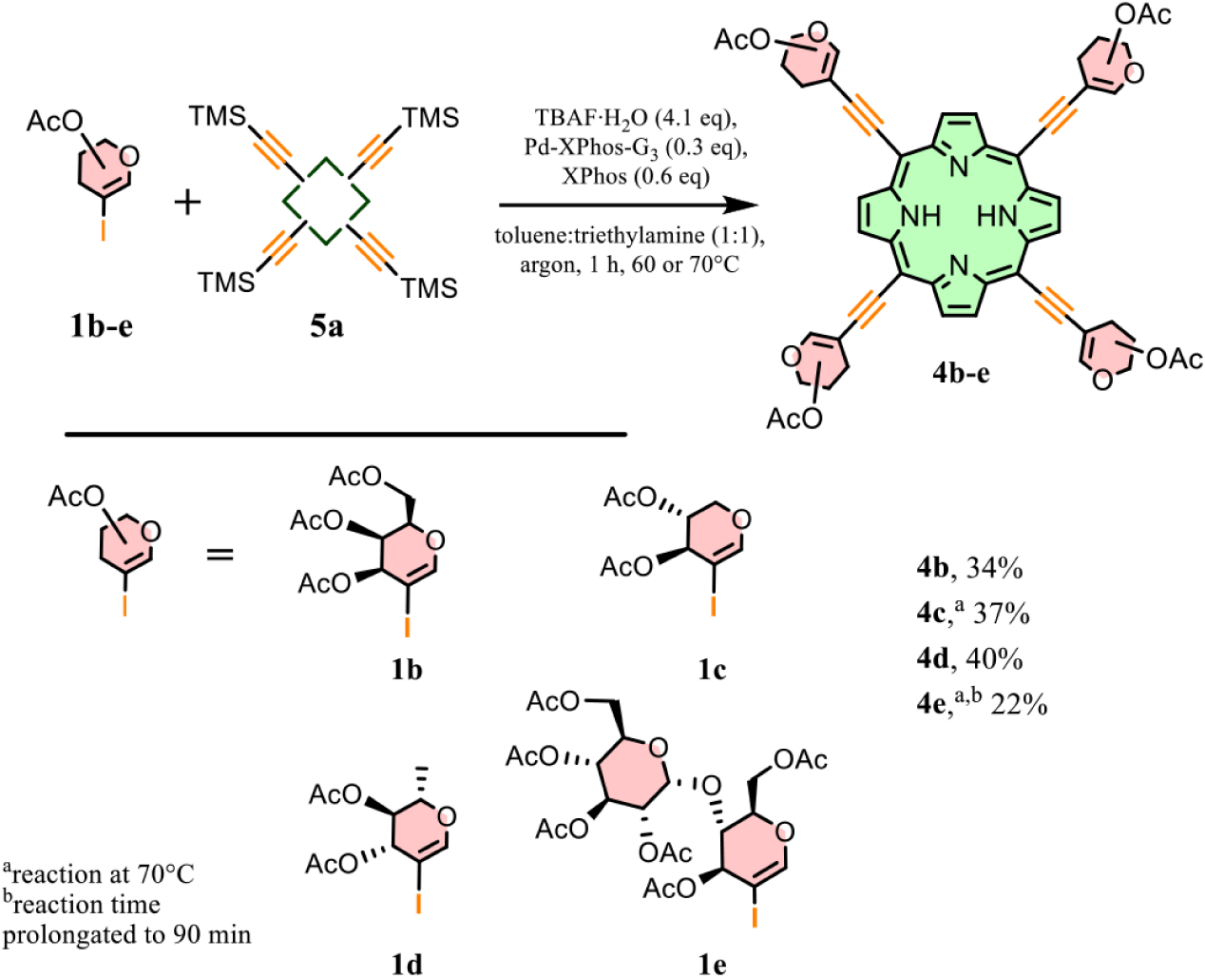
Scope of the Procedure on Regard to Sugar Motif

The series of glycoporphyrins (**4a**–**e**) was then investigated using UV–vis measurements (Figure 1). All of the hybrids were characterized by a bathochromic shift of the absorption bands. For the final application, the most interesting region was the Q-band absorption, from the least energetic wavelengths. In the case of all the hybrids, glycoconjugates absorbed light in the biological window region, in the range of 740–780 nm, with absorption maxima at 745–756 nm. It seems that the d-galactal motif adopts the most efficient conformation for conjugation with the macrocycle, and therefore its absorption occurs at the longest wavelengths. The positive impact of our approach is particularly visible when compared with substrate **5a**, for which the Q-band appeared at 710 nm (Figure 1), while all of the glycoconjugates were bathochromically and hyperchromically shifted.

**Figure 1:**
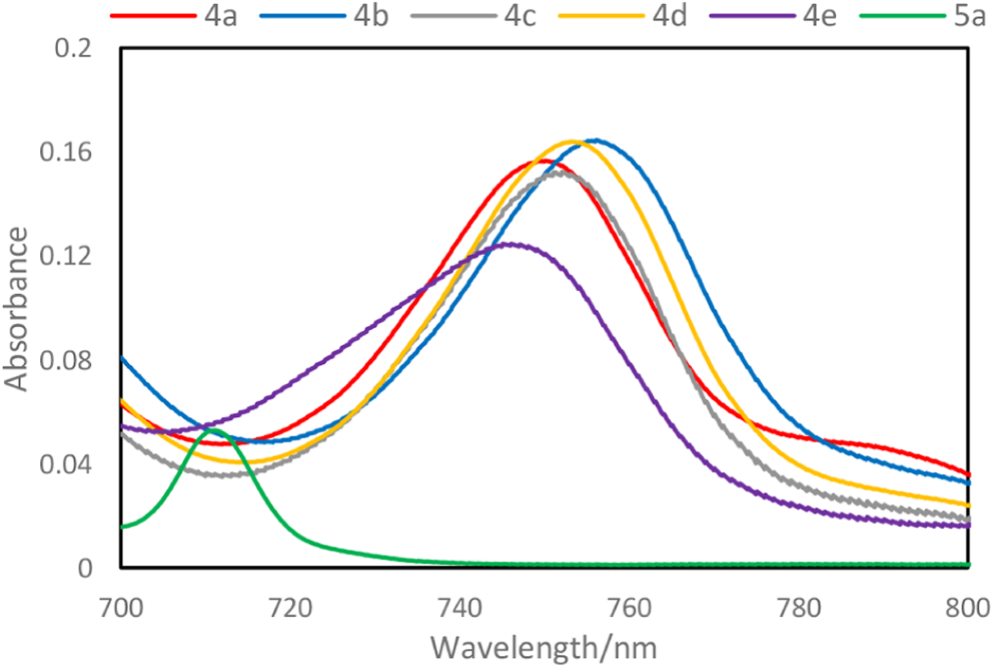
Comparison of the Q bands between glycoporphyrins **4a**–**e** and porphyrin **5a**.

For the initial assessment of the photosensitizing abilities of our compounds to produce singlet oxygen, we followed the established UV–vis protocol with 9,10-dimethylanthracene (DMA) used as an indicator.^71–73^ For the irradiation of **4a**–**e**, we were able to use lower-energy visible light (740 nm lamp), and we monitored the decrease in absorbance of DMA observed at 378 nm. All of the carbohydrate-porphyrin hybrids efficiently generated singlet oxygen, demonstrating their potential for use in PDT (Figure 2).

**Figure 2:**
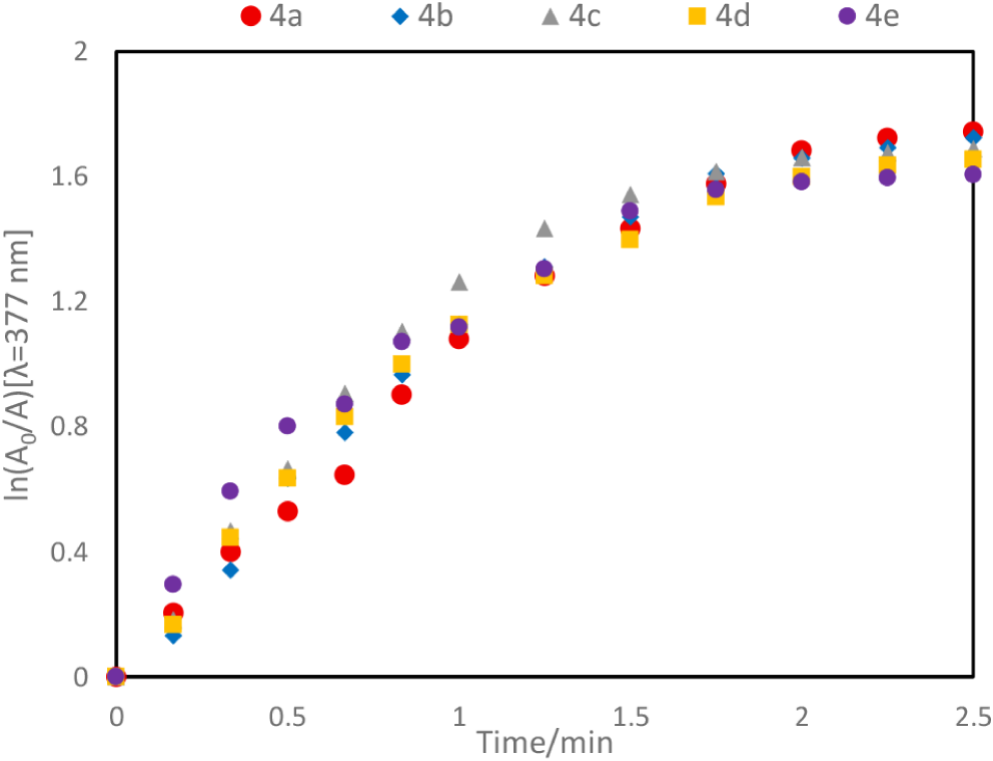
Absorbance change of **4a**–**e** solutions with DMA as a function of irradiation time. DMA (300 μM) and **4a**-**4e** (18 μM), λ_irr_ = 740 nm photosensitized in DMF.

The procedure of the deprotection step was adjusted from our previous studies and exemplified on the d-glucal derivative (Scheme 8, **6a**). Due to the progressive change in solubility of semideprotected glycoconjugates, we performed the reaction in a two-step process: initially in a mixture of solvents compatible with **4a** (DCM:MeOH, 1:1), after 2 h, the solvents were replaced with more compatible ones to solubilize the more polar semideprotected glycoconjugates (acetone:water, 1:1). Following these modifications, we were able to isolate the final glycoporphyrin **6a** in a good yield of 67%.

**Scheme 8:**
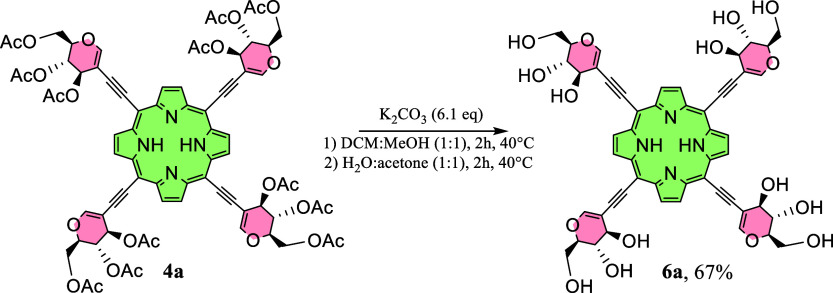
Deacetylation Step towards **6a**

Apart from the convenient UV–vis profile of **6a**, we also considered the benefits of the lack of additional aryl substituents on this type of porphyrin derivative. The parameter of water solubility is still an important matter in current glycoporphyrin studies. These types of structures are often reported as well soluble in aqueous solutions of organic solvents^15,74^ even in highly diluted water systems.^33^ Sole water solubility of sugar-porphyrin hybrids is less frequently reported;^13^ however, some polymer sugar conjugates and glycopolymers are characterized by improved solubility in this particular liquid.^75,76^ To better reflect the affinity of **6a** to the water phase, we decided to determine its 1-octanol/water partition coefficient (log P). As reference glycoporphyrins, we decided to use values determined for A_2_B_2_-type glycosylated porphyrin (determined for phosphate-buffered saline as the aqueous phase)^13^ and A_4_-type glycoporphyrin with additional *meso*-aryl substituents (Scheme 1A).^42,44^

The 1-octanol/water partition coefficient (*P*_o/w_) was determined at 25 °C. P_o/w_ values were calculated and expressed in logarithmic form as a ratio of the concentration of **6a** in octanol to its concentration in water, using the equation log (*P*_o/w_) = log ([**6a**]_o_/[**6a**]_w_) (Table 2). This experiment proved that glycoconjugates directly linked to the porphyrin macrocycle (**6a**) are more hydrophilic than their counterparts with additional *meso*-aryl substituents (Table 2, entries 1,2).

**Table 2:** 1-Octanol/Water Partition Coefficient of Glycoporphyrins

glycoporphyrin	log (P_o/w_)
A_2_B_2_-types	0.45–0.83 ^13^
A_4_-type *meso*-arylglycoporphyrin	1.43 ^44^
**6a**	–0.44

The final aspect of our studies was dedicated to the quantification of PS’s ability to generate singlet oxygen. We applied the procedure for ROS quantum yield measurement, which is based on the comparison of the known reference (**TPP**, 5,10,15,20-tetraphenylporphyrin)^77,78^ with the value determined for our glycoporphyrins (Figure 3, Table 3). For these studies, we exemplified the results using acetylated glycoporphyrin **4a** and its deprotected form (**6a**). Due to the characteristics of the irradiating lamp (λ_irr_ = ± 20 nm), the most reliable results were achieved with the use of the 550 nm lamp (similar, nonzero absorption profile for **TPP** and **4a**, **6a**). Both glycoconjugates were characterized with lower quantum yields of ROS than TPP. Probably, the PS with free hydroxyl groups aggregated in the applied solution and/or singlet oxygen is not the main ROS type produced by this compound. Both of these aspects will be carefully evaluated in further biological studies on the application of these compounds in PDT. Nevertheless, the synthetic access to the deprotected glycoporphyrin, in addition to its ability to generate singlet oxygen, makes it an interesting target for challenging these compounds in photomedicine assays.

**Figure 3:**
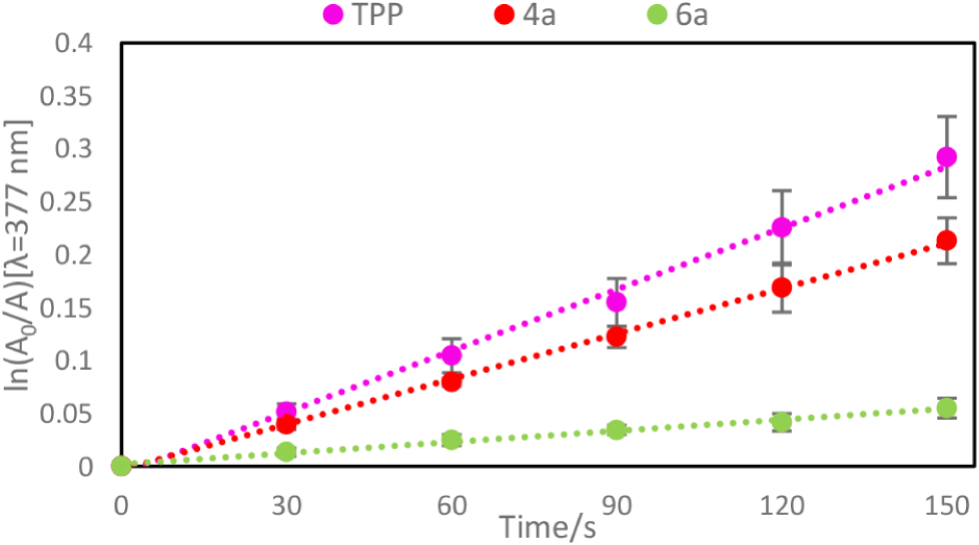
First-order plots for the photooxidation of DMA (300 μM), photosensitized by one of the PS: **TPP**, **4a**, or **6a** in DMF; λ_irr_ = 550 nm. Values represent ± standard deviation of three separate experiments. Molarities of PS were adjusted to equalize absorption at 550 nm.

**Table 3:** Kinetic Parameters for the Photooxidation of DMA: Observed Rate Constant (*k*_obs_) and Singlet Molecular Oxygen Quantum Yield (*Φ_Δ_*) in DMF

PS	TPP	4a	6a
$k_{obs}^{DMF}\left(s^{-1}\right)$	(1.87 ± 0.21)·10^–3^	(1.40 ± 0.11)·10^–3^	(0.37 ± 0.05)·10^–3^
$\Phi_{\Delta }^{DMF}$	0.64 ^79^	0.48 (±0.09)	0.13 (±0.03)

## Conclusions

Herein, we propose a new synthetic procedure toward red-shifted glycoporphyrins characterized by significant water solubility. These structures efficiently absorbed light wavelengths from the region of the biological window. The Sonogashira-based strategy allowed us to efficiently cross-couple 2-iodoglycals, including disaccharide-type glycals, with the *meso*-tetraethynyl porphyrin scaffold. All of the glyconjugates were able to generate singlet oxygen, proving their promising character as candidates for third-generation PSs in photodynamic therapy.

## Experimental Methods

### General Information

All chemical operations were carried out using sealed tubes or round-bottom flasks. Acetonitrile (POCH) was distilled under argon over CaH_2_. Triethylamine (Chempur) was dried over KOH. Toluene (Chempur) was dried over molecular sieves (4Å). Other solvents were used without further purification. ^1^H NMR (600 MHz) and ^13^C NMR (151 MHz) were recorded at 293 K. Chemical shifts are given in ppm (δ) and are referenced to internal solvent signal or to TMS used as an internal standard. Optical rotations were measured on a polarimeter (Anton-Paar MCP-150) at 598 nm. ${[\alpha ]}_{D}^{20}$ is expressed in °·cm^3^·g^‑1^·dm^‑1^, and *c* is expressed in g/100 cm³. High-resolution mass spectra (HRMS, ESI) and mass spectra (MS) were measured on an LTQ Orbitrap Velos spectrometer in positive ionization mode. UV–vis spectra and single-wavelength absorbance measurements for ROS production studies were measured on a VWR UV-6300PC spectrophotometer. IR spectra were measured on a Bruker ALPHA II FT-IR spectrometer with a diamond ATR module. Microwave reactions were conducted in an AntonPaar MonoWave 400 microwave reactor. Reactions were monitored by thin-layer chromatography on silica gel plates (VWR, F254 silica, 200 μm layer thickness, aluminum sheets). Sugar derivatives were spotted by immersing in a 5% ethanolic solution of phosphomolybdic acid followed by heating or rendered under an ultraviolet lamp. Porphyrin derivatives were observed under daylight or rendered under an ultraviolet lamp. All reactions were performed using block heaters unless stated otherwise.

### General Procedure for the Glycosylation of Porphyrin **5a**

To a sealed tube equipped with a stirring bar, Pd-XPhos-G3 (7.3 mg, 0.009 mmol, 0.3 equiv), XPhos (8.0 mg, 0.018 mmol, 0.6 equiv), TBAF (37.5 mg, 0.118 mmol, 4.1 equiv), and a 2-iodoglycal (0.232 mmol, 8.0 equiv) were added. The reactor was then flushed with argon and sealed with a rubber septum. To a round-bottom flask equipped with a stirring bar, porphyrin **5a** (20 mg, 0.029 mmol, 1.0 equiv) was added. The flask was then flushed with argon and sealed with a rubber septum. To the reactor, 2 mL of toluene and 2 mL of triethylamine were added through the rubber septum, and the reactor was heated to 70 °C (or, in some particular procedures, to 60 °C) using a heating mantle. To the flask, 10 mL of toluene and 10 mL of triethylamine were added through the rubber septum, and the flask was heated to 80 °C, also using a heating mantle.

The flask and the reactor were then connected by a cannula, which allowed the transfer of the porphyrin solution to the reactor. The porphyrin solution was added dropwise to the reactor by increasing argon pressure in the flask. The reaction mixture was stirred for 75 min (counted from the moment the addition started; 105 min was applied for **4e**). Upon completion, the mixture was filtered through a short pad of Celite and washed with toluene. The filtrate was concentrated *in vacuo*, and flash chromatography was used to purify the product.

### General Procedure for the Estimation of Quantum Yield by **9**–**10**-Dimethylanthracene (DMA)

2.4 μmol of the tested porphyrin was dissolved in 10.0 mL of DMF. For TPP, a similar solution was prepared using 2.4 μmol of porphyrin and 100 mL of DMF. From the prepared solutions, a specific volume was taken and added to the quartz cell to make the final solution’s absorbance equal to 0.05 at 550 nm (0.50 mL for **TPP**, 0.41 mL for **4a**, 0.18 mL for **6a**). Next, 1.00 mL of a 0.6 mM solution of DMA in DMF was added to the quartz cell. Lastly, the quartz cell was filled up to 2.0 mL with DMF. The cell’s contents were mixed to ensure a stable absorbance reading.

The test sample was irradiated with an EvoluChem 550 PF lamp (18 W, 550 nm) from a distance of 24 cm. After 30 s of irradiation, the absorbance of the test sample at 377 nm wavelength light was measured using a spectrophotometer. This procedure was then repeated until a total irradiation time of 2.5 was reached. For each porphyrin, 3 tests were performed.

Control samples without a photosensitizer were prepared using 1.00 mL of DMF and 1.00 mL of DMA solution. Blank samples without DMA were prepared by using appropriate volumes of tested porphyrin solutions and then filled up to 2.0 mL with DMF.

## Supplementary Material



## Data Availability

Data for this article, including original NMR.fid files and UV–vis measurements, are available at Zenodo database [DOI: 10.5281/zenodo.19257685]

## Notes

Supporting Information includes experimental procedures, spectroscopic characterization, and copies of NMR and UV/vis spectra^41,43,61,80,81^ (PDF)

## Abbreviations

DCM: dichloromethane
DMA: 9,10-dimethylantracane
DMF: *N,N*-dimethylformamide
PCC: pyridinium chlorochromate
PDT: photodynamic therapy
PS: photosensitizer
ROS: reactive oxygen species
TFA: trifluoroacetic acid
TPP: 5,10,15,20-tetraphenylporphyrin
