# Supplementary material for: Pursuing the Red-Shift in Glycoporphyrins—A New Synthetic Approach toward Glycoconjugates from the meso-Tetraethynylporphyrin Scaffold
Source: ACS Org Inorg Au. 2026 Jun 25;6(4):438–48. doi: 10.1021/acsorginorgau.6c00033 (PMC13449439; doi:10.1021/acsorginorgau.6c00033)

Pursuing the red-shift in glycoporphyrins – new synthetic approach towards glycoconjugates from *meso*-tetraethynylporphyrin scaffold

Jakub Mitrzak,[a] Maciej Malinowski[a*]

[a] Warsaw University of Technology, Faculty of Chemistry, ul. Noakowskiego 3, 00-664 Warsaw, Poland

General experimental methods

All chemical operations were carried out using sealed tubes or round-bottom flasks. Acetonitrile (POCH) was distilled under argon, over CaH_2. Triethylamine (Chempur) was dried over KOH. Toluene (Chempur) was dried over molecular sieves (4Å). Other solvents were used without further purification.

TMS-propynal, 3,4,6-tri-O-acetyl-D-glucal, Pd-XPhos-G3 were purchased in AmBeed; pyrrole, NIS, XPhos, Dess-Martin reagent were purchased in Fluorochem; $BF_3 \cdot Et_2O$, propargyl alcohol were purchased in ThermoScientific; DDQ, 3,4-di-O-acetyl-L-rhamnal, CuI were purchased from Sigma Aldrich; $AgNO_3$, $TBAF \cdot 3H_2O$ were purchased from ApolloScientific; K2CO3 was purchased in POCH.

$Pd(PPh_3)_2Cl_2$ was synthesized following the procedure:

$Pd(PPh_3)_2Cl_2$ synthesis:

The mixture of $PdCl_2$ (5.60 g, 0.0316 mol, 1.0 eq), NaCl (7.01 g, 0.120 mol, 3.80 eq), and MeOH (500 mL) was refluxed for 40 min in a 1L round-bottom flask. Then, solution of PPh_3 (17.39 g, 0.0663 mol, 2.10 eq) in $CHCl_3$ (25 mL) was added. The resulting mixture turned very dense and intensive shaking was applied to improve ligand exchange. The mixture was stirred for additional 2 hours, then filtrated through a pad of cotton. The solid residue was washed with $CHCl_3$ (50 mL) and dried overnight under air atmosphere giving 21.6 g (97%) $Pd(PPh_3)_2Cl_2$ as yellow powder-like solid.

^{1}H NMR (600 MHz) and ^{13}C NMR (151 MHz) were recorded at 293 K. Chemical shifts are given in ppm (δ) and are referenced to internal solvent signal or to TMS used as an internal standard. Multiplicities are declared as follows: s (singlet), broad s (broad singlet), d (doublet), t (triplet), p (pentet), dd (doublet of doublets), ddd (doublet of doublets of doublets), dt (doublet of triplets), td (triplet of doublets), tdd (triplet of doublets of doublets), m (multiplet). Coupling constants J are given in Hz. Optical rotations were measured on polarimeter (Anton-Paar MCP-150) at 598 nm. $[\alpha]_D^{20}$ is expressed in °·cm^3 ·g-1 ·dm-1 and c is expressed in g/100 cm3 . High resolution mass spectra (HRMS, ESI) and mass spectra (MS) were measured on a LTQ Orbitrap Velos spectrometer, in positive ionization mode. UV-Vis spectra and single-wavelength absorbance measurements for ROS production studies were measured on VWR® UV-6300PC spectrophotometer. IR spectra were measured on a Bruker ALPHA II FT-IR spectrometer with a diamond ATR module. Microwave reactions were conducted in an AntonPaar MonoWave 400 microwave reactor.

Reactions were monitored by thin-layer chromatography on silica gel plates (VWR, F254 silica, 200 μm layer thickness, aluminium sheets). Sugar derivatives were spotted by immersing in 5% ethanolic solution of phosphomolybdic acid followed by heating or rendered under ultraviolet lamp. Porphyrin derivatives were observed under daylight or rendered under ultraviolet lamp. All reactions were performed using block heaters unless stated otherwise.

Table of contents

1. ROS generation experiments 4

   1.1 General procedure for ROS production detection by 9-10-dimethylanthracene (DMA) photooxidation 4

   1.2 General procedure for the estimation of quantum yield by 9-10-dimethylanthracene (DMA) photo-oxidation 4

2. General procedure for the measurement of 1-octanol/water partition coefficient 4

3. Optimisation studies on Sonogashira cross-coupling between peracetylated 2-iodo-D-glucal (**1a**) and *meso*-tetra(trimethylsilylethynyl)-porphyrin (**5a**) 5

   3.1. General optimisation studies 5

   3.2. Substrate dosing studies 6

   3.3. Catalyst studies 6

4. Synthesis of substrates 7

5. Synthesis of new compounds 12

   5.1. Synthesis and characterisation of new propargyl alcohol and aldehyde peracetylated D-glucal derivatives 12

   5.2. Synthesis and characterisation of new glycoporphyrins with different sugar motifs (**4a-e**) 14

   5.3. Synthesis and characterisation of deprotected glycoporphyrin **6a** 20

6. Reactive oxygen species generation plots of new glycoporphyrins 22

7. UV-vis spectra of porphyrin compounds 24

8. NMR spectra of known compounds 27

9. NMR spectra of new compounds 28

1. ROS generation experiments

1.1 General procedure for ROS production detection by 9-10-dimethylanthracene (DMA) photooxidation

2.4 μmol of tested porphyrin was dissolved in 10.0 mL of DMF. From this solution, 0.15 mL was taken and added to a quartz cell. Next, 0.85 mL of DMF was added, followed by 1.00 mL of 0.6 mM solution of DMA in DMF. Cell content was mixed to ensure stable absorbance reading.

The test sample was irradiated by an EvoluChem 740 PF lamp (18 W, 740 nm) from a distance of 24 cm. After 10 seconds of irradiation, the absorbance of the test sample of 377 nm wavelength light was measured using a spectrophotometer. This procedure was then repeated until 1 minute of total irradiation time. Later, irradiation time increased to 15 seconds between each absorbance measurement, until 2.5 minutes of total irradiation time.

Control samples without photosensitizer were prepared using 1.00 mL of DMF and 1.00 mL of DMA solution. Blank samples without DMA were prepared using 0.15 mL of tested porphyrin solution and 1.85 mL of DMF.

For **5a** due to its unsatisfactory solubility in DMF, tested solution was prepared in chloroform. This solution was later diluted with DMF by using the same protocol as any other samples of photosensitisers.

1.2 General procedure for the estimation of quantum yield by 9-10-dimethylanthracene (DMA) photo-oxidation

2.4 μmol of tested porphyrin was dissolved in 10.0 mL of DMF. For TPP, a similar solution was made using 2.4 μmol of porphyrin and 100 mL of DMF. From prepared solutions, such volume was taken and added to the quartz cell to make the final solution's absorbance equal to 0.05 at 550 nm (0.50 mL for **TPP**, 0.41 mL for **4a**, 0.18 mL for **6a**). Next, 1.00 mL of 0.6 mM solution of DMA in DMF was added to the quartz cell. Lastly, the quartz cell was filled up to 2.0 mL with DMF. Cell content was mixed to ensure stable absorbance reading.

The test sample was irradiated by an EvoluChem 550 PF lamp (18 W, 550 nm) from a distance of 24 cm. After 30 seconds of irradiation, the absorbance of the test sample of 377 nm wavelength light was measured using a spectrophotometer. This procedure was then repeated until 2.5 minutes of total irradiation time. For each porphyrin, 3 tests were performed.

Control samples without photosensitizer were prepared using 1.00 mL of DMF and 1.00 mL of DMA solution. Blank samples without DMA were prepared using appropriate volumes of tested porphyrin solutions and filled up to 2.0 mL with DMF.

2. General procedure for the measurement of 1-octanol/water partition coefficient

To a 1 μmol of tested porphyrin, 0.5 ml of 1-octanol and 0.5 ml of water were added. Next, this suspension was stirred at 25 °C for 1 h. After that, 50 μL of each phase was taken using syringe with a filter. The filtrated solutions were placed in separate quartz cuvettes and into each of them 1.95 mL of DMF was added. The absorbance of each solution was measured, and the absorbance of the peak of

Soret band was used to calculate the partition coefficient following the equation: $\log(P_{o/w}) = \log([\mathbf{A}]_o/[\mathbf{A}]_w)$.

3. Optimisation studies on Sonogashira cross-coupling between peracetylated 2-iodo-D-glucal (**1a**) and *meso*-tetra(trimethylsilylethynyl)-porphyrin (**5a**)

3.1. General optimisation studies

Entry[1]	Catalyst (eq)	Ligand (eq)	Solvents (1/1; v/v)	Reaction time [h]	Overall yield[2]
1.	Pd-XPhos-G3 (0.2)	XPhos (0.2)	toluene/triethylamine	3	3%
2.	Pd-XPhos-G3 (0.2)	XPhos (0.2)	toluene/triethylamine	18	0%
3.	Pd-XPhos-G3 (0.2)	XPhos (0.2)	toluene/triethylamine	0.5	5%
4.	Pd-XPhos-G3 (0.2)	XPhos (0.2)	toluene/triethylamine	2	13%
5.	Pd-XPhos-G3 (0.08)	XPhos (0.08)	toluene/triethylamine	0.5	0%
6.	Pd-XPhos-G3 (0.3)	XPhos (0.3)	toluene/triethylamine	2	16%
7.	Pd-XPhos-G3 (0.3)	XPhos (0.3)	toluene/triethylamine	48	4%[3]
8.	Pd-XPhos-G3 (0.3)	XPhos (0.3)	acetonitrile/triethylamine	2	12%
9.	Pd-XPhos-G3 (0.3)	XPhos (0.3)	1,4-dioxane/triethylamine	2	10%
10.	Pd-XPhos-G3 (0.3)	XPhos (0.3)	toluene/triethylamine	1	17%
11.	Pd-XPhos-G3 (0.3)	XPhos (0.6)	toluene/triethylamine	1	20%
12.	Pd-XPhos-G3 (0.1)	XPhos (0.2)	toluene/triethylamine	1	trace
13.	Pd-XPhos-G3 (0.3)	XPhos (0.6)	toluene/triethylamine	1	12%[4]

[1] General condition porphyrin **5a** (20 mg, 0.029 mmol, 1.0 eq), 2-iodoglycal **1a** (69 mg, 8.0 eq), catalyst (0.2-0.3 eq), ligand (0.2-0.6 eq), TBAF·$3H_2O$ (37.5 mg, 4.1 eq), solvent 1 + triethylamine (6+6 mL), argon atmosphere, 60°C; [2] Final yield of 4 subsequent reactions, isolated yield of **4a** after chromatographic purification; [3] Reaction at room temperature; [4] volume of the solvents increased to 24 mL

3.2. Substrate dosing studies

Entry[1]	Catalyst (eq)	Ligand (eq)	Solvents (1/1; v/v)	Reaction time [h]	Porphyrin dosing	Overall yield
1.	Pd-XPhos-G3 (0.3)	XPhos (0.6)	toluene/triethylamine	1	**5a** solid portionwise addition (3 portions, 5 min interval)	22%
2.	Pd-XPhos-G3 (0.3)	XPhos (0.6)	toluene/triethylamine	1	**5a** solid portionwise addition (4 portions, 5 min interval)	26%
3.	Pd-XPhos-G3 (0.3)	XPhos (0.6)	toluene/triethylamine	1	**5a** portionwise injection (14 portions, 3 min interval)	33%
4.	Pd-XPhos-G3 (0.3)	XPhos (0.6)	toluene/triethylamine	1	**5a** continuous injection (20 min)	41%
5.	Pd-XPhos-G3 (0.3)	XPhos (0.6)	toluene/triethylamine	1	**5a** continuous injection (20 min)[2]	17%
6.	Pd-XPhos-G3 (0.3)	XPhos (0.6)	toluene/triethylamine	1	**5a** continuous injection (20 min)[3]	44%

[1] General condition porphyrin **5a** (20 mg, 0.029 mmol, 1.0 eq), 2-iodoglycal **1a** (69 mg, 8.0 eq), catalyst (0.2-0.3 eq), ligand (0.2-0.6 eq), TBAF·3H_2O (37.5 mg, 4.1 eq), solvent 1 + triethylamine (12+12 mL), argon atmosphere, 60°C; [2] Reaction at 50°C; [3] Reaction at 70°C, 5-fold scale reaction

3.3. Catalyst studies

Entry[1]	Catalyst (eq)	Ligand (eq)	Solvents (1/1; v/v)	Reaction time [h]	Porphyrin dosing	Overall yield
1.	$Pd(OAc)_2$ (0.3)	XPhos (0.6)	toluene/triethylamine	18	-	0%
2.	$Pd(OAc)_2$ (0.2), CuI (0.4 eq)	BrettPhos (0.2)	toluene/triethylamine	1	-	0%
3.	$Pd_2(dba)_3$ (0.15)	$AsPh_3$ (1.6)	toluene/triethylamine	3	-	0%
4.	$Pd_2(dba)_3$ (0.15)	$AsPh_3$ (1.6)	toluene/triethylamine	24	-	0%[2]
5.	$Pd(PPh_3)_2Cl_2$ (0.2)	CuI (0.2)	toluene/triethylamine	24	-	0%[3]
6.	Pd-BrettPhos-G3 (0.3)	BrettPhos (0.6)	toluene/triethylamine	1	-	5%
7.	Pd-BrettPhos-G3 (0.3)	BrettPhos (0.6)	toluene/triethylamine	1	**5a** portionwise injection (14 portions, 3 min interval)	9%
8.	Pd-tBuXPhos-G3 (0.3)	tBuXPhos (0.6)	toluene/triethylamine	1	**5a** continuous injection (20 min)	0%

[1] General condition porphyrin **5a** (20 mg, 0.029 mmol, 1.0 eq), 2-iodoglycal **1a** (69 mg, 8.0 eq), catalyst (0.2-0.3 eq), ligand (0.2-0.6 eq), TBAF·3H_2O (37.5 mg, 4.1 eq), solvent 1 + triethylamine (6+6 mL), argon atmosphere, 60°C; [2] Reaction at room temperature; [3] Reaction at 70°C

4. Synthesis of substrates

Meso-tetra(trimethylsililethynyl)porphyrin (**5a**)

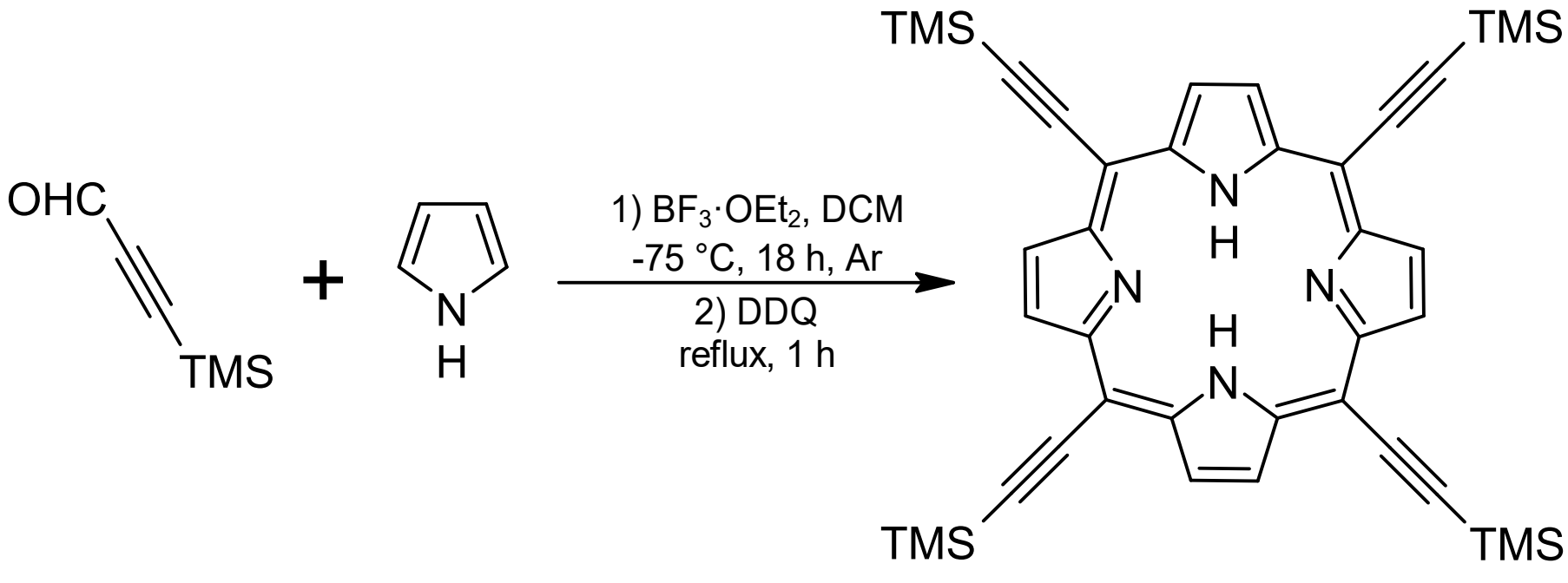

5a

To a round-bottom flask equipped with a stir bar, dichloromethane (560 mL) and 3-trimethylsililpropionaldehyde (3.00 mL, 20.3 mmol, 1.0 eq) were added. Next, stirring was started and the solution was flushed with argon and cooled down to -70 °C in an acetone bath. Pyrrole (1.45 mL, 21.0 mmol, 1.0 eq) was subsequently added through a rubber septum, under argon. Then solution of BF_3·OEt_2 in DCM (5.75 μg/mL, 0.04 mol/dm^3) was added (0.40 mL, 0.46 mg, 3.28 mmol, 0.16 eq) dropwise. Upon completion, the reaction mixture was stirred for a further 3 hours in -75 °C, and then at room temperature for 18 hours. After that, DDQ (4.85 g, 21.4 mmol, 1.05 eq) was added and the mixture was refluxed in a heating mantle for 1 hour. The reaction mixture was then filtered through a layer of silica in a large-diameter porosity funnel (G-2 type), which was then washed three times with DCM and once with chloroform. The filtrate was then concentrated to dryness with silica.

Residual solid was purified by flash chromatography (hexane/DCM, 4:1, (v/v)) yielding porphyrin **5a** (567.4 mg, 16%). The characterisation data was in agreement with literature[1].

***R**$_f$* = 0.46 (hexane/DCM, 4:1, (v/v))

^{1}H NMR (600 MHz, $CDCl_3$) δ [ppm]: 9.50 (s, 8H, H_β), 0.62 (s, 36H, H_{TMS}), -1.92 (s, 2H, NH)

UV-Vis ($CHCl_3$): λ_{max} [nm] (log ε): 710.9 nm (4.01), 647.5 (3.82), 607.5 (4.72), 568.1 (4.18), 452.7 (5.55; Soret band)

General procedure for 2-iodination of glycals using microwave irradiation[2]

Glycal (1.0 eq), NIS (1.2 eq), $AgNO_3$ (0.2 eq) and acetonitrile (2.1 mL/mmol of glycal) were added into a glass vial. Next, the vial was sealed and immediately put in Monowave 400 microwave reactor. Reaction was divided into 4 subsequent steps during which mixture was constantly stirred:

1. Slow heating with microwaves to 35 °C for 30 seconds (average microwave power: 10 W).
2. Rapid heating to 150 °C with microwaves (pick microwave power: 300 W, average microwave power: 100 W average step time: 1.0 minutes).
3. Holding 150 °C for 2.5 minutes (average microwave power: 16 W).
4. Cooling down to 60 °C (average step time: 2 minutes).

After reaction, mixture was filtrated through a viscose wool and washed with chloroform (for monosaccharide derivatives) or ethyl acetate (for polysaccharide derivatives). Filtrate was then concentrated to dryness with silica. Residue was purified by flash chromatography yielding desired 2-iodoglycal.

2-iodo-3,4,6-tri-O-acetyl-D-glucal (**1a**)

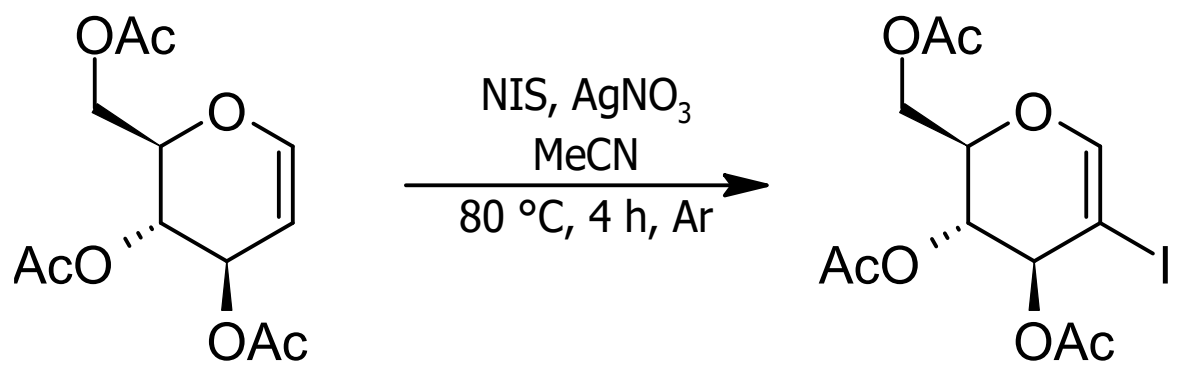

1a

A flame-dried flask, equipped with a stir bar and a condenser was evacuated and backfilled with argon 3 times (Schlenck technique). Next, 40 mL of freshly distilled MeCN (distillation over CaH_2, under argon) was added, under constant flow of argon. The solvent was preheated to 80 °C using a heating mantle. After that 3,4,6-tri-O-acetyl-D-glucal (5.00 g, 18.37 mmol, 1.0 eq), $AgNO_3$ (0.63 g, 3.71 mmol, 0.2 eq), and NIS (4.98 g, 22.1 mmol, 1.2 eq) were added under constant flow of argon. The last portion of MeCN (10 mL) was then added and used to flush the substrate remains from flask walls. The mixture was stirred under argon in darkness at 80 °C for 4 hours. Upon completion, the mixture was filtered through a short pad of Celite® and washed with EtOAc. The filtrate was concentrated to dryness with silica.

Residue was purified by flash chromatography (hexane (until violet colour was removed), to hexane/ethyl acetate, 8:2, (v/v)) yielding glycal **1a** (2.82 g, 39%) in the form of a yellow oil, which crystalised at 4 °C.

Microwave-assisted procedure for **1a**

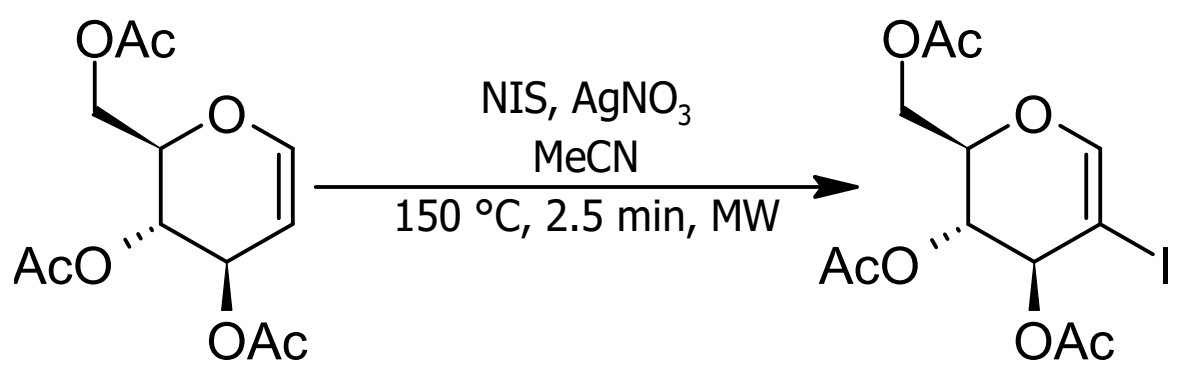

1a

In accordance with the general procedure, 273 mg (1.00 mmol) of 3,4,6-tri-O-acetyl-D-glucal, 270 mg (1.2 mmol) of NIS and 34 mg (0.20 mmol) of $AgNO_3$ were used.

Residue was purified by flash chromatography (hexane (until violet colour was removed), to hexane/ethyl acetate, 8:2, (v/v)) yielding glycal **1a** (286 mg, 72%) in the form of yellowish crystals.[2]

The characterisation data was in agreement with literature[3].

R_f = 0.43 (hexane/ethyl acetate, 7:3, (v/v))

^{1}H NMR (600 MHz, $CDCl_3$) δ [ppm]: 6.78 (d, J = 1.3 Hz, 1H), 5.49 (ddd, J = 5.2, 1.2, 0.7 Hz, 1H), 5.22 (dd, J = 7.0, 5.2 Hz, 1H), 4.41-4.37 (m, 2H), 4.20-4.17 (m, 1H), 2.11 (s, 3H) , 2.07 (s, 3H), 2.06 (s, 3H)

2-iodo-3,4,6-tri-O-acetyl-D-galactal (**1b**)

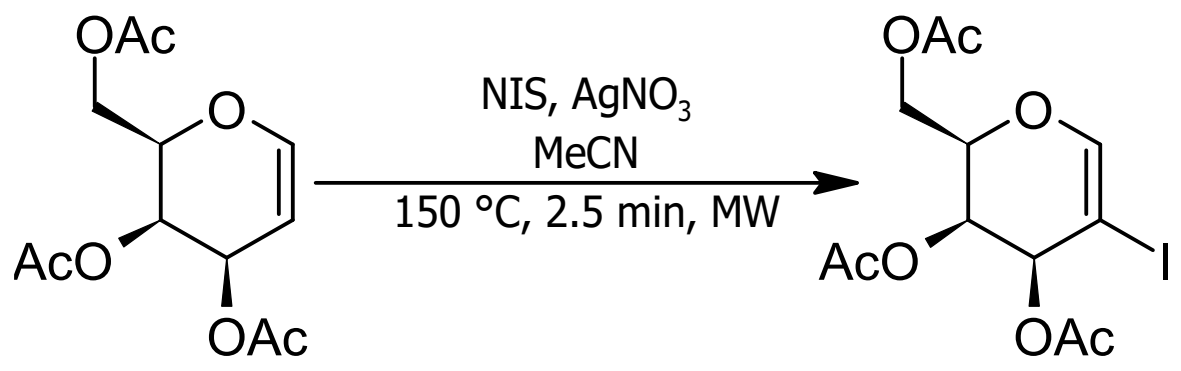

1b

In accordance with the general procedure, 273 mg (1.00 mmol) of 3,4,6-tri-O-acetyl-D-galactal, 270 mg (1.2 mmol) of NIS and 34 mg (0.20 mmol) of $AgNO_3$ were used.

Residue was purified by flash chromatography (hexane (until violet colour was removed), to hexane/ethyl acetate, 8:2, (v/v)) yielding glycal **1b** (232 mg, 58%) in the form of yellowish crystals. The characterisation data was in agreement with literature[4].

R$_f$ = 0.62 (hexane/ethyl acetate, 6:4, (v/v))

^{1}H NMR (600 MHz, $CDCl_3$) δ [ppm]: 6.78 (d, *J* = 1.6 Hz, 1H), 5.59 (ddd, *J* = 4.6, 1.6, 1.0 Hz, 1H), 5.50 (dd, *J* = 4.6, 2.1 Hz, 1H), 4.43 (t, *J* = 6.0 Hz, 1H), 4.27 (dd, *J* = 11.8, 7.5 Hz, 1H), 4.20 (dd, *J* = 11.8, 5.3 Hz, 1H), 2.13 (s, 3H), 2.09 (s, 3H), 2.08 (s, 3H)

2-iodo-3,4-di-O-acetyl-D-xylal (**1c**)

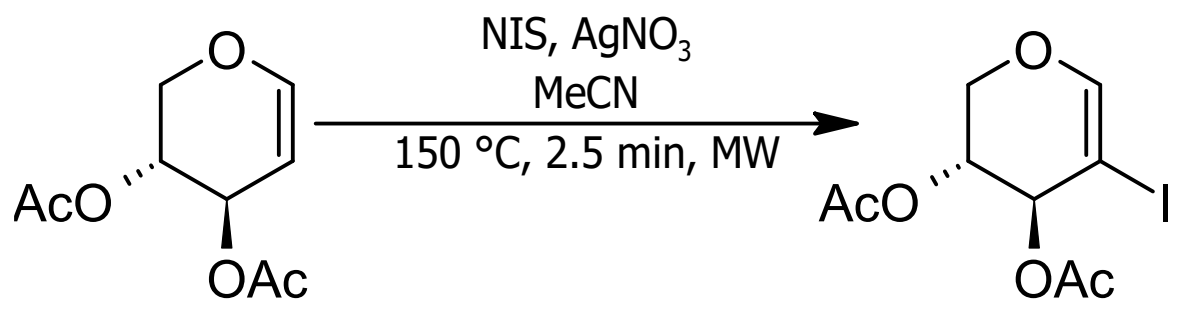

1c

In accordance with the general procedure, 105 mg (0.53 mmol) of 3,4-di-O-acetyl-D-xylal, 142 mg (0.63 mmol) of NIS and 18 mg (0.11 mmol) of $AgNO_3$ were used.

Residue was purified by flash chromatography (hexane (until violet colour was removed), to hexane/ethyl acetate, 8:2, (v/v)) yielding glycal **1c** (119 mg, 70%) in the form of yellowish oil. The characterisation data was in agreement with literature[4].

R$_f$ = 0.45 (hexane/ethyl acetate, 85:15, (v/v))

^{1}H NMR (600 MHz, $CDCl_3$) δ [ppm]: 6.92 (s, 1H), 5.27 – 5.25 (m, 1H), 4.97 (td, *J* = 2.6, 1.6 Hz, 1H), 4.30 (ddd, *J* = 12.4, 2.8, 1.7 Hz, 1H), 3.97 (dd, *J* = 12.4, 1.6 Hz, 1H), 2.14 (s, 3H), 2.11 (s, 2H)

2-iodo-3,4-di-O-acetyl-L-rhamnal (**1d**)

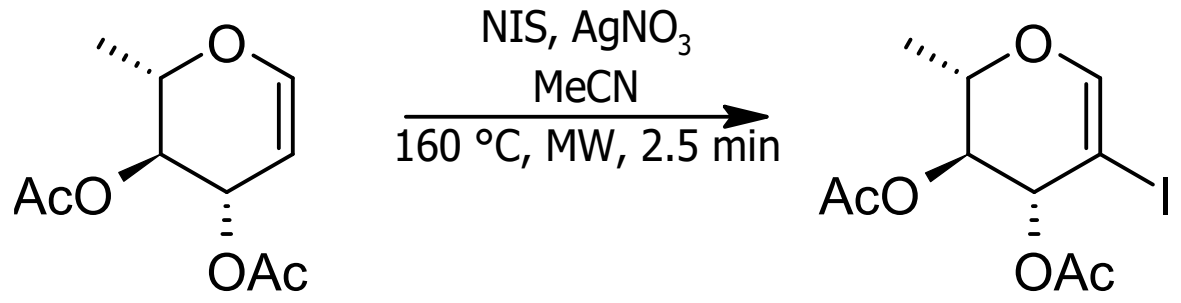

1d

To a microwave reactor equipped with a stirring bar, 3,4-di-O-acetyl-L-rhamnal (1.12 g, 5.23 mmol, 1.0 eq), NIS (1.27 g, 5.64 mmol, 1.1 eq) and $AgNO_3$ (171.8 mg, 1.01 mmol, 0.2 eq). The reactor was then flushed with argon and 10 mL of MeCN was added through a rubber septum. The reactor was then sealed. The reaction mixture was then rapidly heated under microwave irradiation to 160 °C and kept at this temperature for 2.5 minutes. Upon completion, the mixture was filtered through a short pad of Celite® and washed with EtOAc. The filtrate was concentrated to dryness with silica.

Residue was purified by flash chromatography (hexane (until violet colour was removed), to hexane/ethyl acetate, 9:1, (v/v)) yielding glycal **1d** (1.23 g, 69%) in the form of an orange oil. The characterisation data was in agreement with literature[3].

R_f = 0.30 (hexane/ethyl acetate, 9:1, (v/v))

^{1}H NMR (600 MHz, $CDCl_3$) *δ* [ppm]: 6.75 (d, *J* = 1.2 Hz, 1H), 5.47 (ddd, *J* = 5.3, 1.3, 0.8 Hz, 1H), 5.02 (dd, *J* = 7.3, 5.3 Hz, 1H), 4.25 (p, *J* = 6.9 Hz, 1H), 2.10 (s, 3H), 2.07 (s, 3H), 1.32 (d, *J* = 6.6 Hz, 3H)

2-iodo-2',3,3',4',6,6'-hexa-O-acetyl-D-maltal (**1e**)[2]

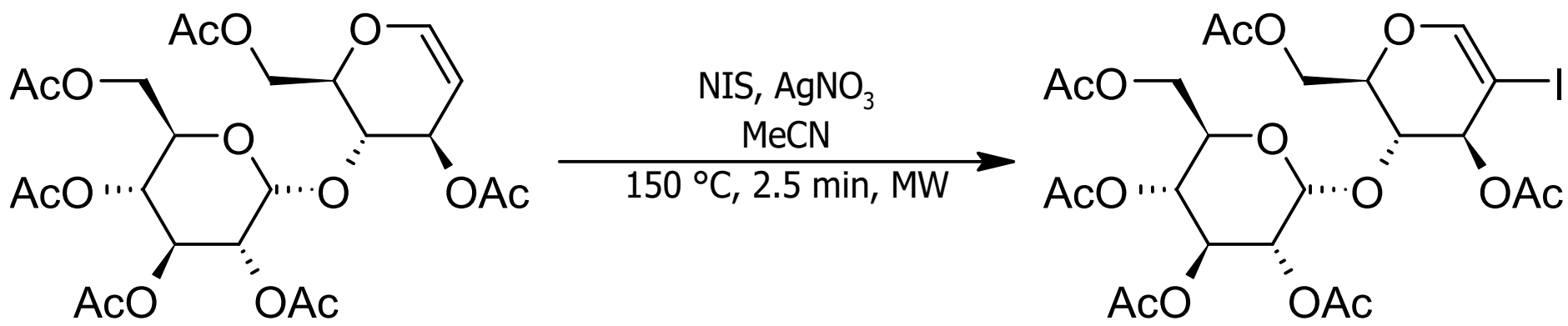

1e

In accordance with the general procedure, 280 mg (0.50 mmol) of 2',3,3',4',6,6'-hexa-O-acetyl-D-maltal, 135 mg (0.60 mmol) of NIS and 17 mg (0.10 mmol) of $AgNO_3$ were used.

Residue was purified by flash chromatography (hexane (until violet colour was removed), to hexane/ethyl acetate, 6:4, (v/v)) yielding glycal **1e** (242 mg, 70%) in the form of yellowish oil. The characterisation data was in agreement with literature[5].

R_f = 0.22 (hexane/ethyl acetate, 6:4, (v/v))

^{1}H NMR (600 MHz, $CDCl_3$) δ [ppm]: 6.80 (broad s, 1H), 5.41 (t, *J* = 9.9 Hz, 1H), 5.37 (d, *J* = 3.9 Hz, 1H), 5.17 (d, *J* = 1.6 Hz, 1H), 5.03 (dd, *J* = 10.3, 9.5 Hz, 1H), 4.84 (dd, *J* = 10.3, 4.0 Hz, 1H), 4.58 – 4.55 (m, 1H), 4.34 (dd, *J* = 12.1, 7.5 Hz, 1H), 4.22 – 4.17 (m, 3H), 4.05 (ddd, *J* = 10.3, 4.8, 2.3 Hz, 1H), 4.00 (t, *J* = 3.2 Hz, 1H), 2.12 (s, 3H), 2.09 (s, 3H), 2.08 (s, 3H), 2.03 (s, 3H), 2.02 (s, 3H), 2.00 (s, 3H)

5. Synthesis of new compounds

5.1. Synthesis and characterisation of new propargyl alcohol and aldehyde peracetylated D-glucal derivatives

2-(prop-2-yn-1-ol)-3,4,6-tri-O-acetyl-D-glucal (**2a**)

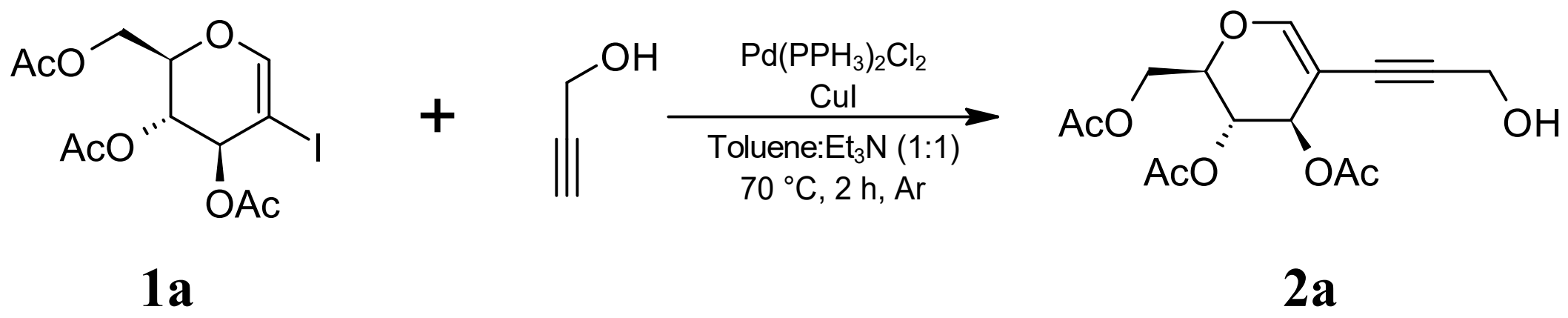

To a sealed tube equipped with a stirring bar 2-iodoglycal **1a** (525.2 mg, 1.32 mmol, 1.0 eq), $Pd(PPh_3)_2Cl_2$ (65.0 mg, 0.093 mmol, 0.07 eq), and CuI (36.6 mg, 0.19 mmol, 0.14 eq) were added. The reactor was flushed with argon and 7.5 mL of toluene,7.5 mL of triethylamine, and propargyl alcohol (0.16 mL, 2.64 mmol, 2.0 eq) were added through a rubber septum. The reactor was then sealed and heated to 70 °C using a heating mantle. The reaction was stirred for 2 hours. Upon completion, the reaction mixture was concentrated *in vacuo*.

Residue was purified by flash chromatography (DCM, to DCM/ethyl acetate, 7:3, (v/v)) yielding glycal **2a** (341.7 mg, 80%) in the form of a yellow oil.

$\boldsymbol{R_f}$ = 0.54 (DCM/ethyl acetate, 7:3, (v/v)); $[\boldsymbol{\alpha}]_{\boldsymbol{D}}^{\mathbf{20}}$ = +17.0° (1.00, $CHCl_3$)

^{1}H NMR (600 MHz, $CDCl_3$) δ [ppm]: 6.86 (s, 1H, H-1_{glycal}), 5.48 (d, J = 5.3 Hz, 1H, H-3_{glycal}), 5.17 (dd, J = 6.6, 5.2 Hz, 1H, H-4_{glycal}), 4.39 (dd, J = 12.2, 6.2 Hz, 1H, H-6_{glycal}), 4.34-4.32 (m, 3H, H-5_{glycal},-$\underline{CH_2}$OH), 4.18 (dd, J = 12.1, 3.3 Hz, 1H, H H-6'$_{glycal}$), 2.09 (s, 3H, $C\underline{H_3}$COO-), 2.08 (s, 3H, $C\underline{H_3}$COO-), 2.07 (s, 3H, $C\underline{H_3}$COO-), 1.55 (s, 1H, OH)

^{13}C{^{1}H} NMR (151 MHz, $CDCl_3$) δ [ppm]: 170.5; 170.3; 169.5; 151.4; 96.3; 88.1; 80.2; 74.4; 67.1; 66.4; 61.1; 51.6; 20.9; 20.8; 20.7

IR (film) 3478 (O-H), 2164 (C≡C), 1739 (C=O), 1630 (C=C), 1434 (C-H), 1368 (C-H), 1218, 1177, 1021 (C-O), 962, 910 cm^{-1}

HRMS (ESI) m/z: $[M+H]^+$ Calcd for $C_{15}H_{19}O_8$ 327.1074; Found 327.1070

2-(prop-2-yn-1-al)-3,4,6-tri-O-acetyl-D-glucal (**3a**)

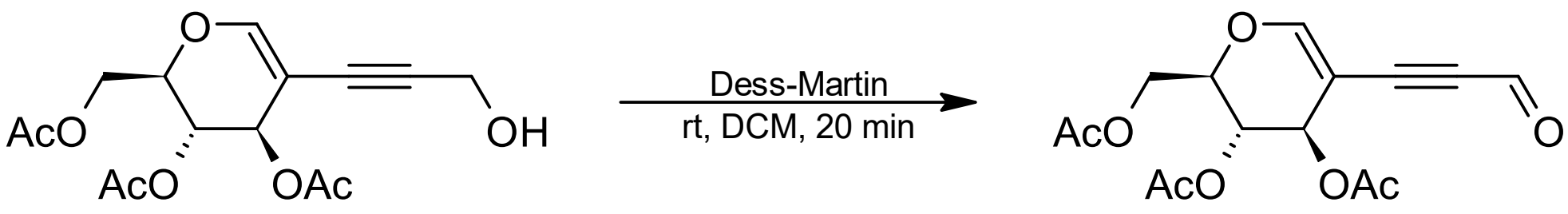

To a round-bottom flask equipped with a stirring bar, glycal **2a** (61.0 mg, 0.19 mmol, 1.0 eq), Dess-Martin periodinane (91.8 mg, 0.22 mmol, 1.16 eq), and 2 mL of DCM were added. The reaction was stirred for 20 minutes at room temperature. Upon completion, 5 mL of water, 15 mL of a saturated sodium thiosulfate solution, and 15 mL of a saturated sodium bicarbonate solution were added. The mixture was then transferred to a separatory funnel, where the phases were separated. The organic phase was then dried over $MgSO_4$ (anh.), and the resulting suspension was filtered and concentrated *in vacuo*.

Residue was purified by flash chromatography (DCM, to DCM/ethyl acetate, 8:2, (v/v)) yielding glycal **3a** (39.3 mg, 63%) in the form of an orange oil.

R_f = 0.89 (DCM/ethyl acetate, 7:3, (v/v)); $[\alpha]_D^{20}$ = +23.0° (1.00, $CHCl_3$)

^{1}H NMR (600 MHz, $CDCl_3$) δ [ppm]: 9.23 (s, 1H, CHO), 7.20 (s, 1H, H-1_{glycal}), 5.51 (dt, *J* = 5.1, 0.9 Hz, 1H, H-3_{glycal}), 5.20 (dd, *J* = 6.1, 5.1 Hz, 1H, H-4_{glycal}), 4.46 (tdd, *J* = 6.1, 3.3, 1.0 Hz, 1H, H-5_{glycal}), 4.42 (dd, *J* = 12.1, 6.4 Hz, 1H, H-6_{glycal}), 4.20 (dd, *J* = 12.1, 3.2 Hz, 1H, H-6’$_{glycal}$), 2.11 (s, 3H, C$\underline{H}_3$COO-), 2,09 (s, 3H, C$\underline{H}_3$COO-), 2,08 (s, 3H, C$\underline{H}_3$COO-)

^{13}C{^{1}H} NMR (151 MHz, $CDCl_3$) δ [ppm]: 175.8; 170.4; 169.9; 169.3; 157.5; 94.6; 92.2; 91.3; 75.3; 65.8; 65.7; 65.7; 60.8; 20.7; 20.7

IR (film) 2176 (C≡C), 1740 (C=O), 1656 (C=O), 1607 (C=O), 1433 (C-H), 1369 (C-H), 1216, 1186, 1025 (C-O), 954, 906 cm^{-1}

HRMS (ESI) m/z: $[M+H]^+$ Calcd for $C_{15}H_{17}O_8$ 325.0918; Found 325.0916

5.2. Synthesis and characterisation of new glycoporphyrins with different sugar motifs (**4a-e**)

General procedure for the glycosylation of porphyrin 5a

To a sealed tube equipped with a stirring bar Pd-XPhos-G3 (7.3 mg, 0.009 mmol, 0.3 eq), XPhos (8.0 mg, 0.018 mmol, 0.6 eq), TBAF (37.5 mg, 0.118 mmol, 4.1 eq), and a 2-iodoglycal (0.232 mmol, 8.0 eq). The reactor was then flushed with argon and sealed with a rubber septum. To a round-bottom flask equipped with a stirring bar, porphyrin **5a** (20 mg, 0.029 mmol, 1.0 eq) was added. The flask was then flushed with argon and sealed with a rubber septum. To the reactor 2 mL of toluene and 2 mL of triethylamine were added through the rubber septum, and the reactor was heated to 70 °C (or in some particular procedures to 60°C) using a heating mantle. To the flask 10 mL of toluene and 10 mL of triethylamine were added through the rubber septum, and the flask was heated to 80 °C also using a heating mantle.

The flask and the reactor were then connected by a cannula that allowed the transfer of the porphyrin solution to the reactor. The porphyrin solution was added dropwise to the reactor by increasing argon pressure in the flask. The reaction mixture was stirred for 75 minutes (counted from the moment of start of the addition; 105 minutes was applied for **4e**). Upon completion, the mixture was filtered through a short pad of Celite® and washed with toluene. The filtrate was concentrated *in vacuo* and flash chromatography was used to purify the product.

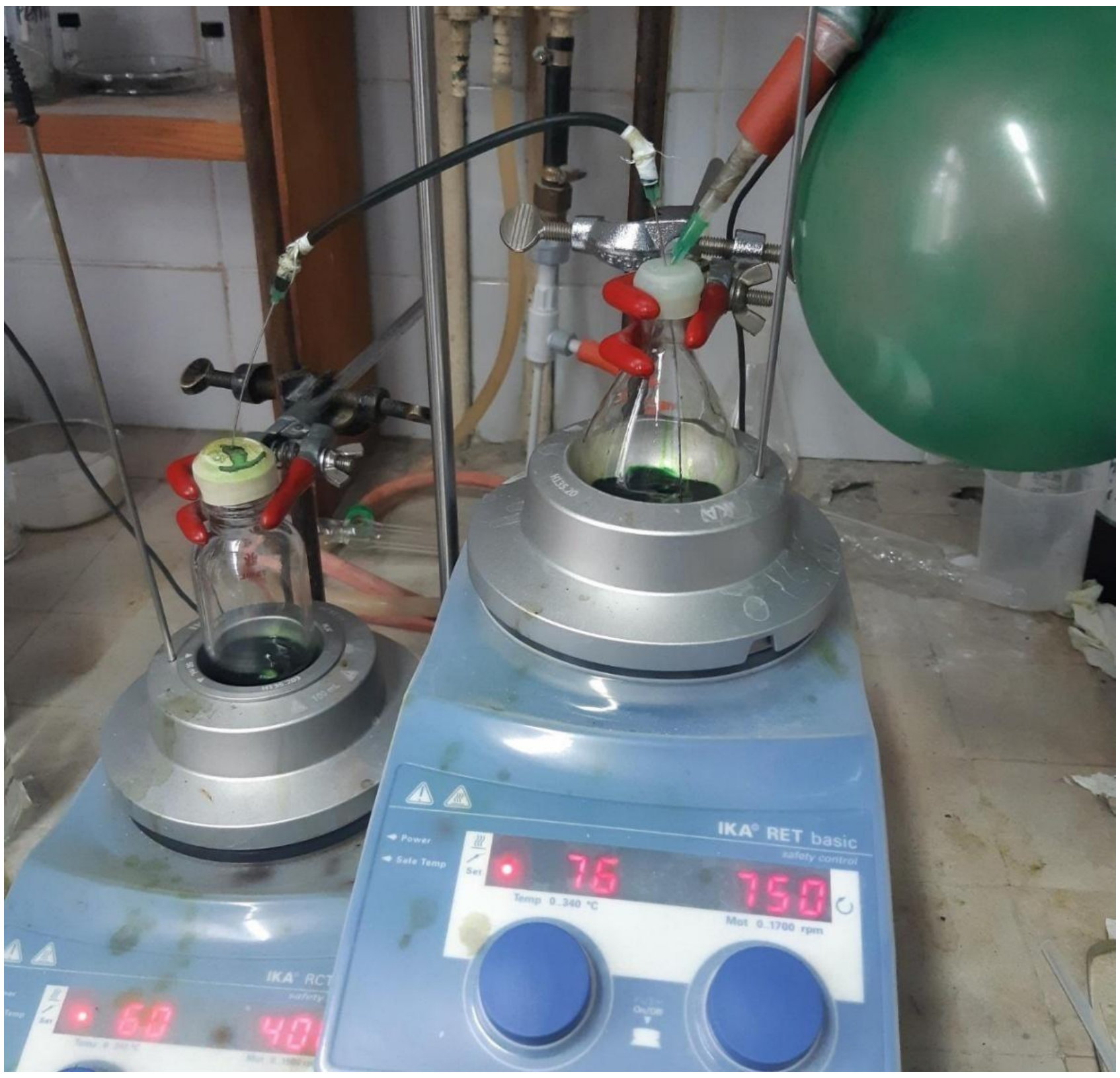

Picture 1. The reaction setup for the glycosylation of porphyrin **5a**.

5,10,15,20-tetrakis((3,4,6-tri-O-acetyl-D-glucal-2-yl)ethynyl)porphyrin (**4a**)

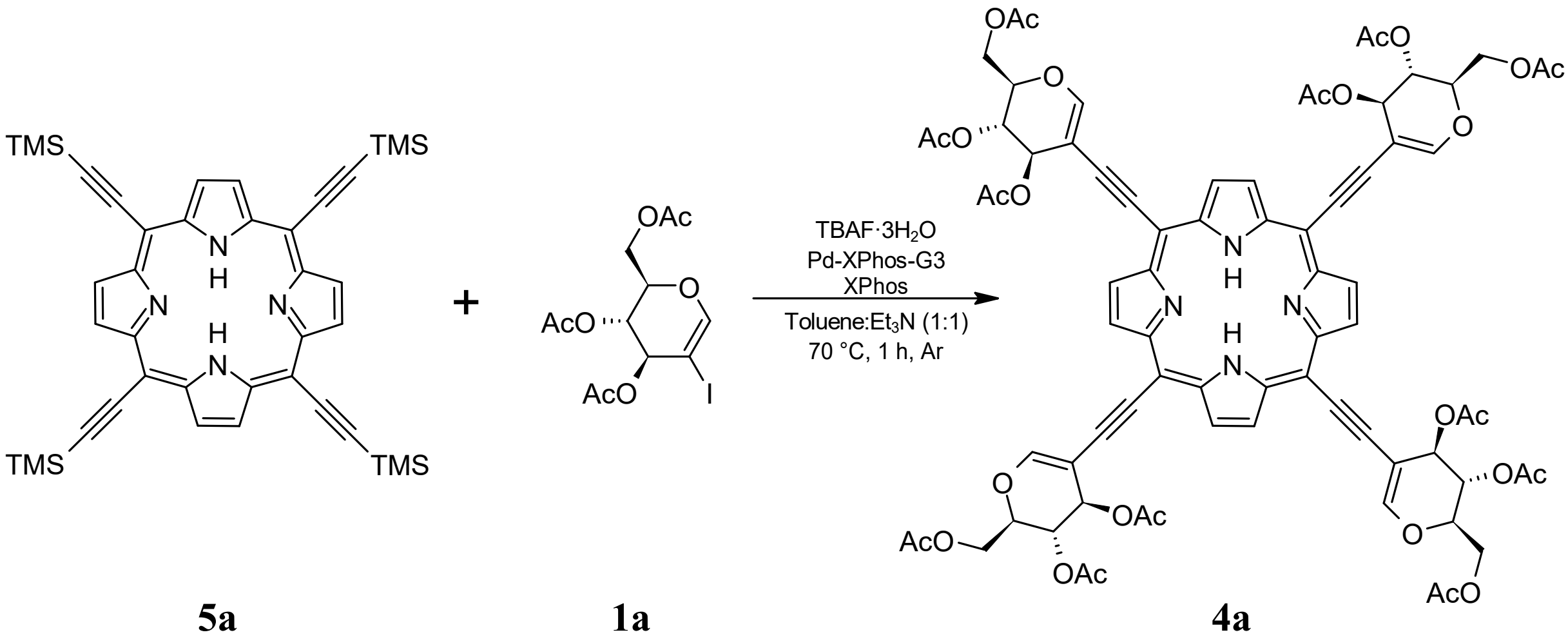

5a **1a** **4a**

In accordance with the general procedure (5-fold scale), 460.8 mg (1.16 mmol) of 2-iodoglycal **1a**, 100 mg (0.145 mmol) of porphyrin **5a**, 36.9 mg (0.045 mmol) of Pd-XPhos-G3, 40.2 mg (0.090 mmol) of XPhos and 191.2 mg (0.590 mmol) of TBAF was used. The product was purified by flash chromatography (DCM (until brown was removed), to DCM/ethyl acetate, 9:1, (v/v) to chloroform/methanol, 200:3, (v/v)) yielding porphyrin **4a** (95.5 mg, 44%) in the form of a violet crystalline solid. Analytical sample was obtained by crystallisation ($CHCl_3$/cyclohexane, 1:3, (v/v), 1.0 mL of $CHCl_3$ per 54.6 mg of **4a**, solvents added at room temperature and left in a refrigerator for 30 minutes).

R_f = 0.23 ($CHCl_3$/MeOH, 200:3, (v/v)), mp: >200 °C

^{1}H NMR (600 MHz, $CDCl_3$) δ [ppm]: 9.32 (s, 8H, H_β), 7.50 (s, 4H, H-1_{glycal}), 6.10 (d, J = 5.3 Hz, 4H, H-3_{glycal}), 5.48-5.46 (m, 4H, H-4_{glycal}), 4.64-4.59 (m, 8H, H-$5,6_{glycal}$), 4.41-4.38 (m, 4H, H-6'$_{glycal}$), 2.27 (s, 12H, C<u>H</u>$_3$COO-), 2.20 (s, 12H, C<u>H</u>$_3$COO-), 2.18 (s, 12H, C<u>H</u>$_3$COO-), -1.53 (s, 2H, NH)

^{13}C{^{1}H} NMR (151 MHz, $CDCl_3$) δ [ppm]: 170.6; 170.5; 169.7; 151.8; 102.7; 97.9; 92.3; 92.0; 74.9; 67.3; 66.8; 61.3; 21.2; 21.0; 20.9

UV-Vis ($CHCl_3$): λ_{max} [nm] (log ε): 750.1 nm (4.26), 653.5 (4.64), 476.7 (5.33; Soret band)

HRMS (ESI) m/z: $[M+H]^+$ Calcd for $C_{76}H_{71}N_4O_{28}$ 1487.4249; Found 1487.4245

5,10,15,20-tetrakis((3,4,6-tri-O-acetyl-D-galactal-2-yl)ethynyl)porphyrin (**4b**)

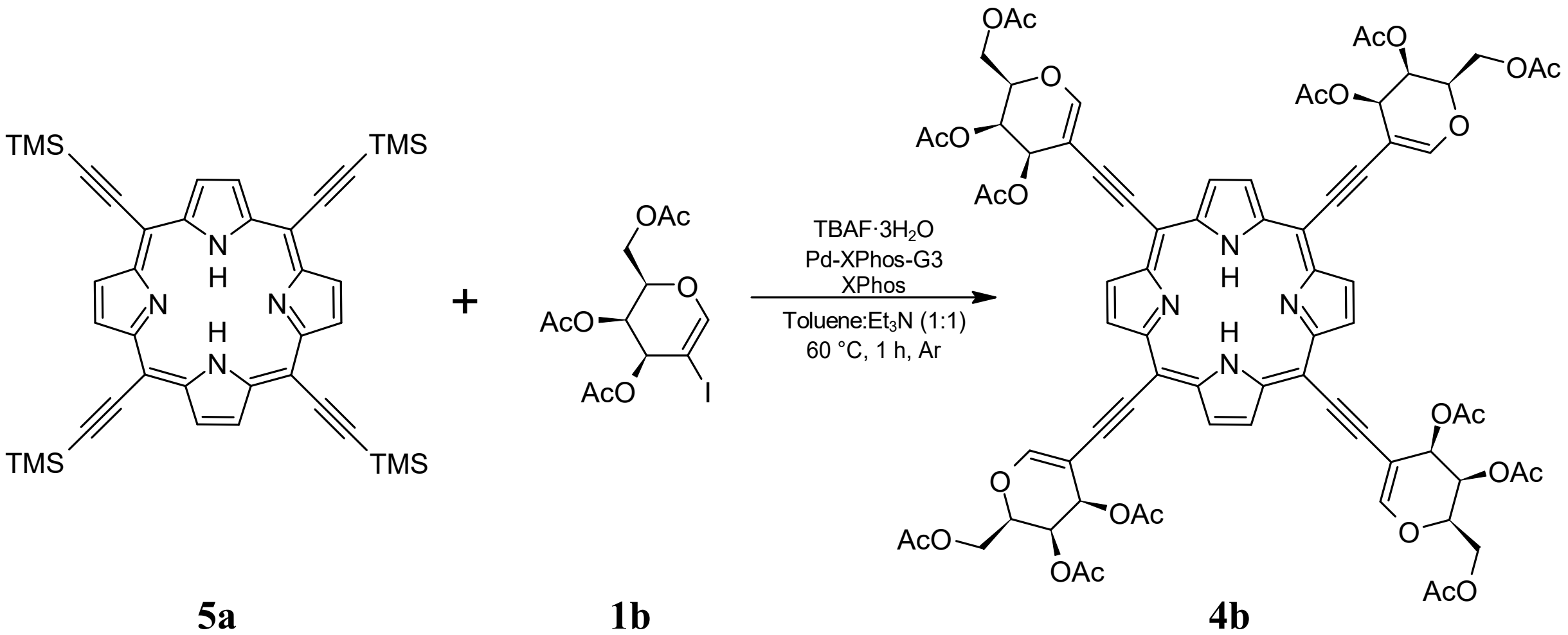

5a **1b** **4b**

In accordance with the general procedure (reactor heated to 60 °C), 91.4 mg (0.23 mmol) of 2-iodoglycal **1b** was used. The product was purified by flash chromatography (DCM (until brown was removed), to DCM/ethyl acetate, 9:1, (v/v), to DCM/ethyl acetate, 8:2, (v/v), to chloroform/methanol, 200:3, (v/v)) yielding porphyrin **4b** (14.7 mg, 34%) in the form of a violet crystalline solid. Analytical sample was obtained by crystallisation ($CHCl_3$/cyclohexane, 1:4, (v/v), 0.30 mL of $CHCl_3$ per 14.7 mg of **4b**, solvents added at room temperature and left in a refrigerator for 30 minutes).

***R**$_f$* = 0.23 ($CHCl_3$/MeOH, 200:3, (v/v)), mp: >200 ℃

^{1}H NMR (600 MHz, $CDCl_3$) *δ* [ppm]: 9.17 (s, 8H, H_β), 7.49 (d, *J* = 1.6 Hz, 4H, H-1_{glycal}), 6.23-6.22 (m, 4H, H-3_{glycal}), 5.74 (dd, *J* = 4.4, 1.8 Hz, 4H, H-4_{glycal}), 4.67 (t, *J* = 6.6 Hz, 4H, H-5_{glycal}), 4.46 (dd, *J* = 11.6, 7.2 Hz, 4H, H-6_{glycal}), 4.40 (dd, *J* = 11.6, 5.7 Hz, 4H, H-$6'_{glycal}$), 2.30 (s, 12H, C$\underline{H}_3$COO-), 2.26 (s, 12H, C$\underline{H}_3$COO-), 2.18 (s, 12H, C$\underline{H}_3$COO-), -1.80 (s, 2H, NH)

^{13}C{^{1}H} NMR (151 MHz, $CDCl_3$) *δ* [ppm]: 170.6; 170.6; 170.3; 151.7; 102.6; 97.9; 92.4; 92.3; 74.2; 64.9; 63.3; 61.8; 21.1; 20.9; 20.8

UV-Vis ($CHCl_3$): λ_{max} [nm] (log *ε*): 756.1 nm (4.24), 659.9 (4.61), 479.0 (5.28; Soret band)

HRMS (ESI) m/z: $[M+H]^+$ Calcd for $C_{76}H_{71}N_4O_{28}$ 1487.4249; Found 1487.4230

5,10,15,20-tetrakis((3,4-di-O-acetyl-D-xylal-2-yl)ethynyl)porphyrin (**4c**)

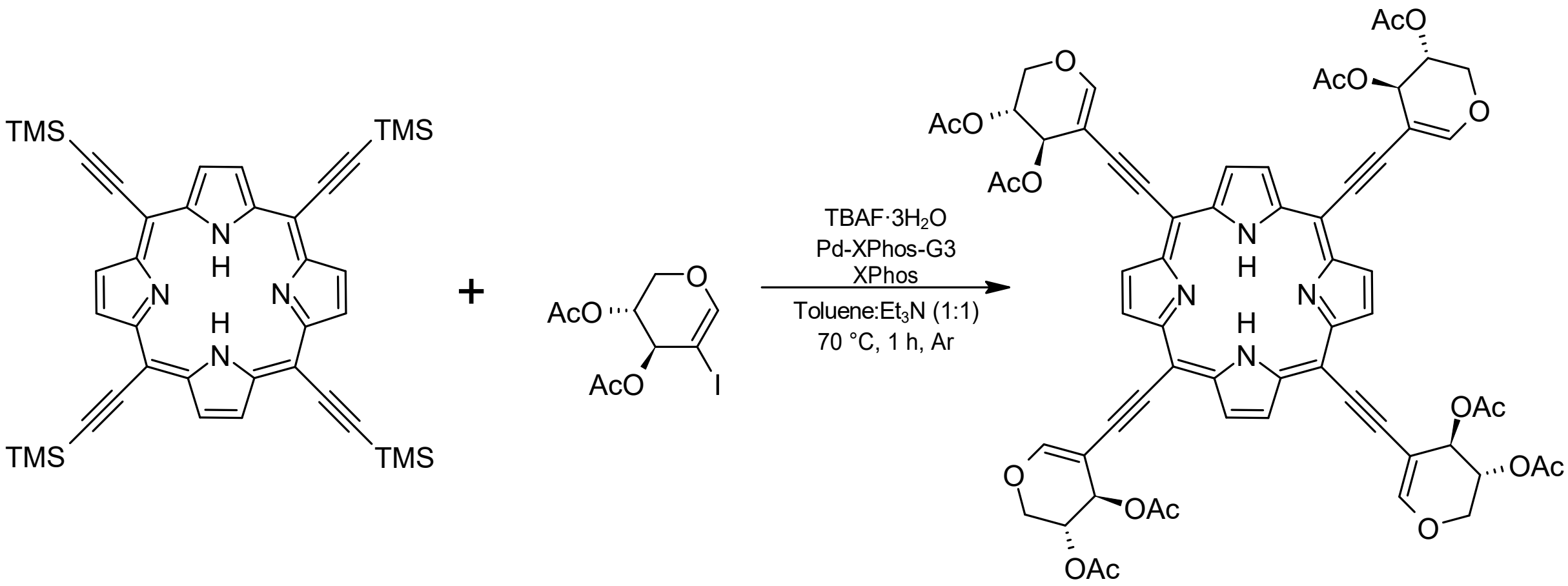

5a **1c** **4c**

In accordance with the general procedure, 75.8 mg (0.23 mmol) of 2-iodoglycal **1c** was used. The product was purified by flash chromatography (DCM (until brown was removed), to DCM/ethyl acetate, 9:1, (v/v), to chloroform/methanol, 200:3, (v/v)) yielding porphyrin **4c** (12.9 mg, 37%) in the form of a violet crystalline solid. Analytical sample was obtained by crystallisation ($CHCl_3$/cyclohexane, 1:3.6, (v/v), 0.28 mL of $CHCl_3$ per 12.9 mg of **4c**, solvents added at room temperature and left in a refrigerator for 30 minutes).

R_f = 0.35 ($CHCl_3$/MeOH, 200:3, (v/v)), mp: >200 ℃

^{1}H NMR (600 MHz, $CDCl_3$) *δ* [ppm]: 9.38 (s, 8H, $H_β$), 7.64 (s, 4H, H-1_{glycal}), 5.90 (t, *J* = 2.2, 1.8 Hz, 4H, H-3_{glycal}), 5.20 (p, *J* = 2.7 Hz, 4H, H-4_{glycal}), 4.51 (dt, *J* = 12.1, 2.1 Hz, 4H, H-5_{glycal}), 4.26 (dd, *J* = 12.3, 1.7 Hz, 4H, H-5'$_{glycal}$), 2.32 (s, 12H, C<u>H</u>$_3$COO-), 2.23 (s, 12H, C<u>H</u>$_3$COO-), -1.43 (s, 2H, NH)

^{13}C{^{1}H} NMR (151 MHz, $CDCl_3$) *δ* [ppm]: 170.0; 169.8; 154.1; 103.0; 96.4; 94.0; 66.6; 64.9; 64.3; 21.3; 21.1

UV-Vis ($CHCl_3$): $λ_{max}$ [nm] (log *ε*): 750.2 nm (3.70), 655.1 (4.09), 476.6 (4.78; Soret band)

HRMS (ESI) m/z: $[M+H]^+$ Calcd for $C_{64}H_{55}N_4O_{20}$ 1198.3326; Found 1198.3299

5,10,15,20-tetrakis((3,4-di-O-acetyl-L-rhamnal-2-yl)ethynyl)porphyrin (**4d**)

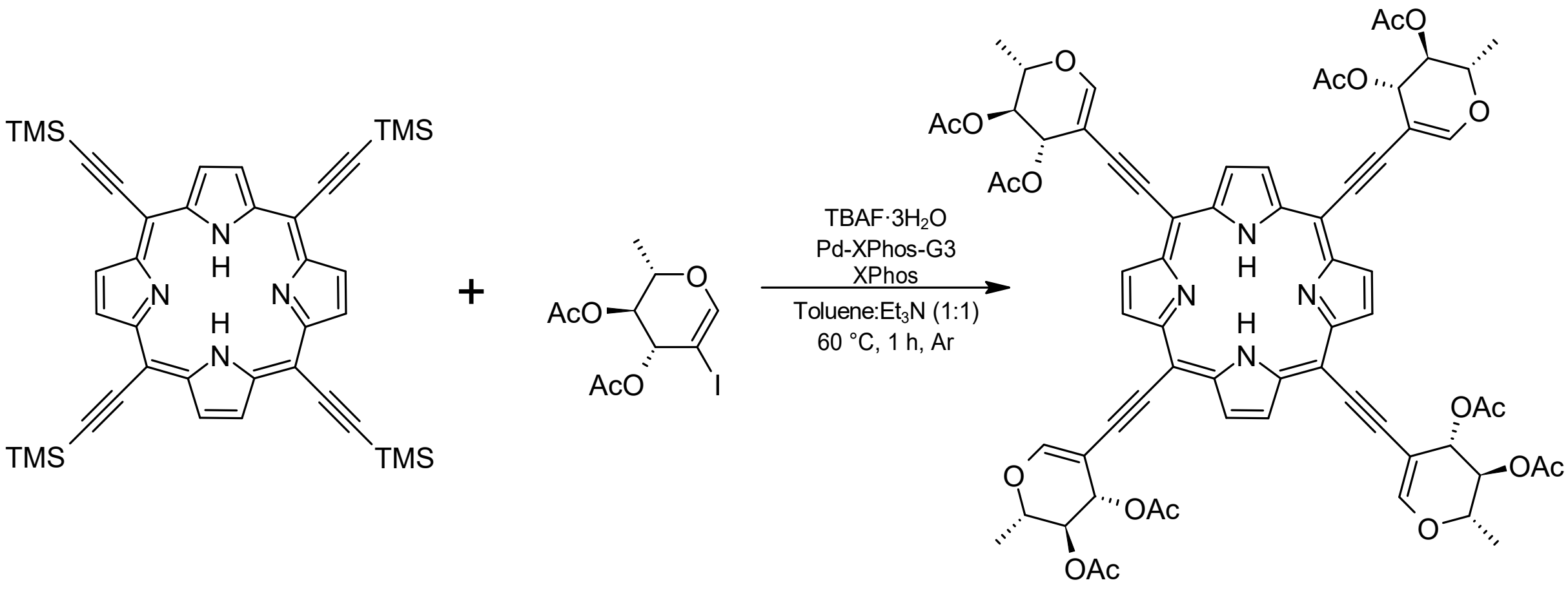

5a **1d** **4d**

In accordance with the general procedure (reactor heated to 60 °C), 78.6 mg (0.23 mmol) of 2-iodoglycal **1d** was used. The product was purified by flash chromatography (DCM (until brown was removed), to DCM/ethyl acetate, 9:1, (v/v) to chloroform/methanol, 200:3, (v/v)) yielding porphyrin **4d** (14.6 mg, 40%) in the form of a violet crystalline solid. Analytical sample was obtained by crystallisation ($CHCl_3$/cyclohexane, 1:4, (v/v), 0.28 mL of $CHCl_3$ per 14.6 mg of **4d**, solvents added at room temperature and left in a refrigerator for 30 minutes).

R_f = 0.27 ($CHCl_3$/MeOH, 200:3, (v/v)), mp: >200 ℃

^{1}H NMR (600 MHz, $CDCl_3$) *δ* [ppm]: 9.15 (s, 8H, $H_β$), 7.50 (s, 4H, H-1_{glycal}), 6.09 (d, *J* = 6.0 Hz, 4H, H-3_{glycal}), 5.28 (dd, *J* = 7.4, 5.6 Hz, 4H, H-4_{glycal}), 4.48 (p, *J* = 6.7 Hz, 4H, H-5_{glycal}), 2.28 (s, 12H, C<u>H</u>$_3$COO-), 2.21 (s, 12H, C<u>H</u>$_3$COO-), 1.54 (d, *J* = 6.6 Hz, 12H, H-6_{glycal}), -2.05 (s, 2H, NH)

^{13}C{^{1}H} NMR (151 MHz, $CDCl_3$) *δ* [ppm]: 170.7; 170.0; 152.4; 102.5; 97.6; 92.5; 91.8; 73.6; 71.4; 68.0; 21.3; 21.1; 16.7

UV-Vis ($CHCl_3$): λ_{max} [nm] (log *ε*): 753.1 nm (4.44), 657.3 (4.82), 477.8 (5.48; Soret band)

HRMS (ESI) m/z: $[M+H]^+$ Calcd for $C_{68}H_{63}N_4O_{20}$ 1255.4030; Found 1255.4011

5,10,15,20-tetrakis((2',3,3',4',6,6'-hexa-O-acetyl-D-maltal-2-yl)ethynyl)porphyrin (**4e**)

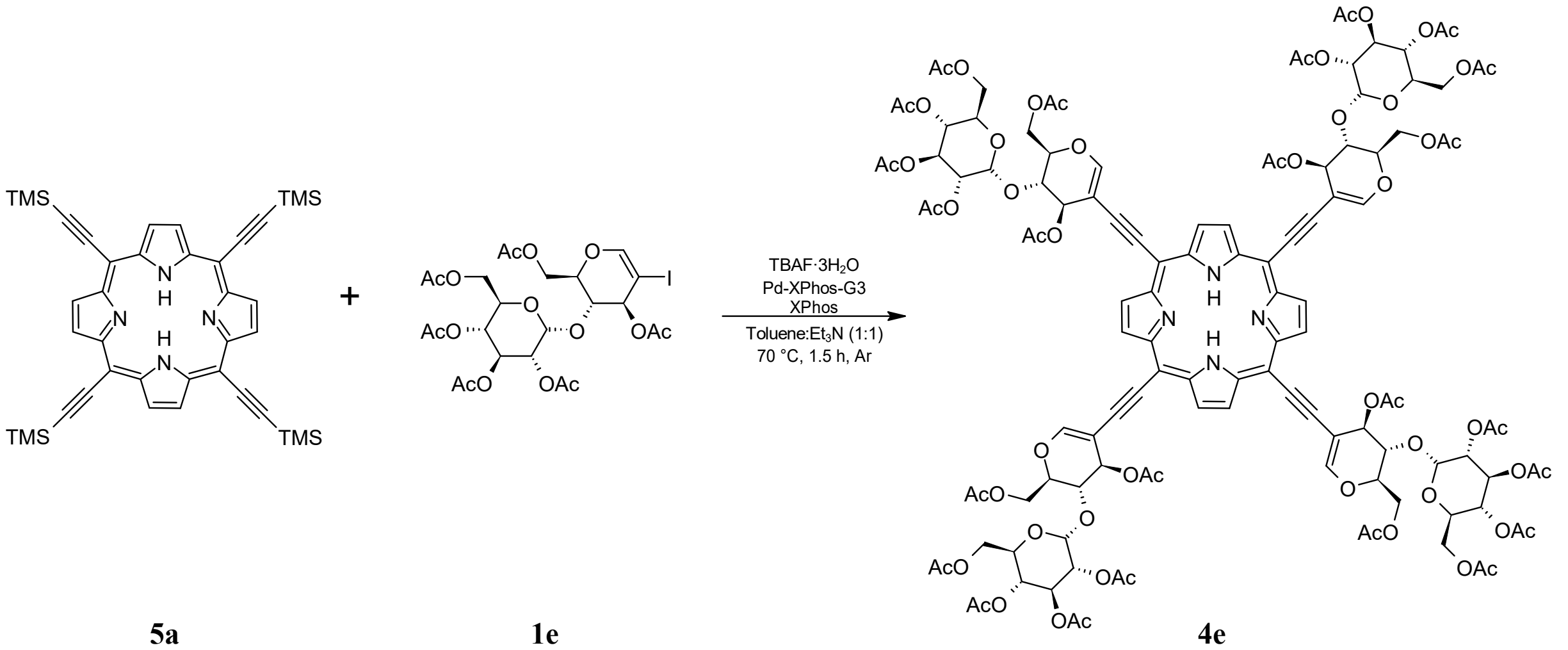

5a **1e** **4e**

In accordance with the general procedure (reaction time extended to 1.5 h), 160.3 mg (0.23 mmol) of 2-iodoglycal **1e** was used, and the reaction was stirred for 90 minutes. The product was purified by flash chromatography (DCM (until brown was removed), to DCM/ethyl acetate, 9:1, (v/v), to chloroform/methanol, 200:3, (v/v)) yielding porphyrin **4e** (16.8 mg, 22%) in the form of a violet crystalline solid. Analytical sample was obtained by crystallisation ($CHCl_3$/cyclohexane, 1:9, (v/v), 0.28 mL of $CHCl_3$ per 16.8 mg of **4e**, solvents added at room temperature and left in a refrigerator for 30 minutes).

***R*$_f$** = 0.32 ($CHCl_3$/MeOH, 200:3, (v/v)), mp: >200 ℃

^{1}H NMR (600 MHz, $CDCl_3$) δ [ppm]: 9.39 (s, 8H, H_β), 7.50 (s, 4H, H-1A_{glycal}), 5.85 (s, 4H, H-3A_{glycal}), 5.63 (d, J = 4.1 Hz, 4H, H-1B_{glycal}), 5.52 (t, J = 9.8 Hz, 4H, H-3B_{glycal}), 5.11 (t, J = 9.9 Hz, 4H, H-4B_{glycal}), 4.93 (dd, J = 10.2, 4.2 Hz, 4H, H-2B_{glycal}), 4.76-4.72 (m, 4H, H-5A_{glycal}), 4.60 – 4.56 (m, 4H, H-6A_{glycal}), 4.49 (dd, J = 12.4, 4.3 Hz, 4H, H-6A'$_{glycal}$), 4.29 (dd, J = 12.2, 4.2 Hz, 4H, H-6B_{glycal}), 4.26 (t, J = 4.0 Hz, 4H, H-4A_{glycal}), 4.22-4.20 (m, 4H, H-6B'$_{glycal}$), 4.19-4.17 (m, 4H, H-5B_{glycal}), 2.26 (s, 12H, C$\underline{H}_3$COO-), 2.20 (s, 12H, C$\underline{H}_3$COO-), 2.15 (s, 12H, C$\underline{H}_3$COO-), 2.14 (s, 12H, C$\underline{H}_3$COO-), 2.06 (s, 12H, C$\underline{H}_3$COO-), 2.00 (s, 12H, C$\underline{H}_3$COO-), -1.36 (s, 2H, NH)

^{13}C{^{1}H} NMR (151 MHz, $CDCl_3$) δ [ppm]: 207.0; 170.7; 170.7; 170.5; 170.4; 170.1; 169.7; 96.5; 72.4; 70.5; 69.9; 68.5; 68.5; 67.4; 61.9; 61.5; 32.0; 31.0; 29.8; 29.4; 27.0; 22.8; 21.2; 20.9; 20.9; 20.8; 20.8; 20.8; 20.7; 20.7; 14.2

UV-Vis ($CHCl_3$): λ_{max} [nm] (log ε): 745.1 nm (3.79), 650.1 (4.20), 468.7 (4.95; Soret band)

HRMS (ESI) m/z: $[M+H]^+$ Calcd for $C_{124}H_{135}N_4O_{60}$ 2640.7664; Found 2640.7703

5.3. Synthesis and characterisation of deprotected glycoporphyrin **6a**

5,10,15,20-tetrakis((D-glucal-2-yl)ethynyl)porphyrin (**6a**)

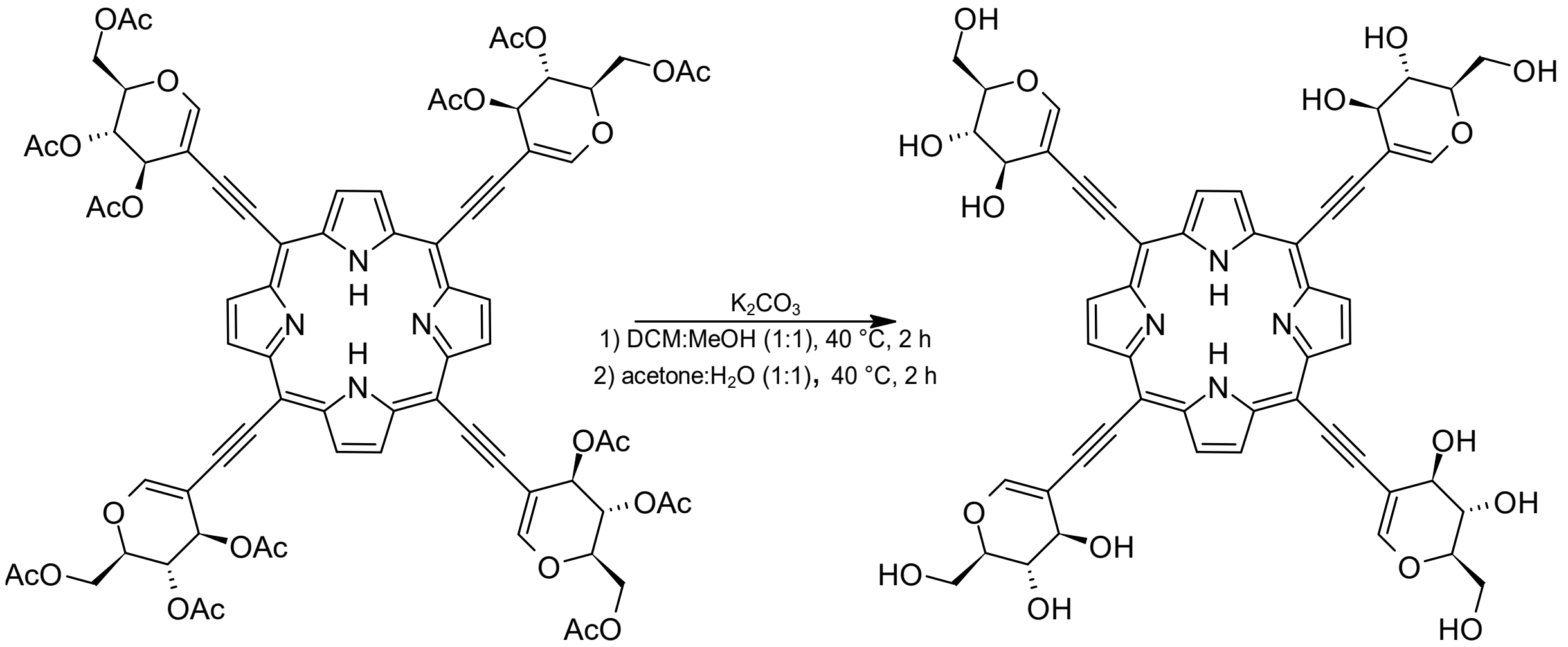

4a **6a**

To a round-bottom flask equipped with a stirring bar, porphyrin **4a** (35.1 mg, 0.024 mmol, 1.0 eq), K_2CO_3 (20.3 mg, 0.147 mmol, 6.1 eq), and 1.3 mL of a DCM/MeOH (1:1, (v/v)) solution were added. The reaction was stirred at 40 °C, by means of a heating mantle, for 2 hours, after which the mixture was concentrated to dryness and 1.3 mL of a water/acetone (1:1, (v/v)) solution was added. The reaction was then stirred for a further 2 hours at 40 °C. Upon completion, the reaction mixture was concentrated to dryness with silica.

Residue was purified by flash chromatography (acetone, to acetone/water acetate, 95:5, (v/v) to acetone/water, 9:1, (v/v)) yielding porphyrin **6a** (15.8 mg, 67%) in the form of a dark violet crystalline solid. Analytical sample was obtained by twice washing with pure boiling acetone.

$\boldsymbol{R_f}$ = 0.61 (acetone/water, 95:5, (v/v)), mp: >200 ℃

^{1}H NMR (600 MHz, DMSO-D_6) δ [ppm]: 9.51 (s, 8H, H_β), 7.60 (s, 4H, H-1_{glycal}), 6.04 (broad s, 4H, OH), 5.75 (broad s, 4H, OH), 4.93 (broad s, 4H, OH), 4.41 (d, J = 6.7 Hz, 4H), 3.99 (ddd, J = 8.4, 5.4, 2.6 Hz, 8H), 3.83 – 3.74 (m, 8H), -0.98 (s, 2H, NH)

^{13}C{^{1}H} NMR (151 MHz, DMSO-D_6) δ [ppm]: 104.2; 102.0; 98.2; 91.1; 81.6; 69.9; 68.7; 60.6

UV-Vis (DMF): λ_{max} [nm] (log ε): 750.1 (3.48), 660.2 (3.86), 469.6 (4.60; Soret band)

HRMS (ESI) m/z: $[M+H]^+$ Calcd for $C_{52}H_{47}N_4O_{16}$ 983.2982; Found 983.2972

[1] Anderson, H. L. Meso-Alkynyl Porphyrins. *Tetrahedron Lett.*, **1992**, *33* (8), 1101–1104. https://doi.org/10.1016/S0040-4039(00)91871-9.

[2] Baran, D.; Kuklewska, A. E.; de Robichon, M.; Stankiewicz, B.; Hurej, K.; Pobudkowska, A.; Plichta, A.; Ferry, A.; Malinowski, M. Rapid Microwave-Assisted Iodination of Glycals to 2-Iodoglycals under Operationally Simple Conditions - a New Chapter in (C-2)-Functionalisation of Sugar Moieties , Chemistry-Methods, *Chemistry–Methods* **2026**, *6* (5). https://doi.org/10.1002/cmtd.70113

[3] Godlewski, B.; Malinowski, M. Suzuki-Miyaura Reaction of Glycals with Base-Labile Protecting Groups as a New Route to Glycoporphyrins. *Adv. Synth. Catal.*, **2024**, *366* (13), 2956–2966. https://doi.org/10.1002/adsc.202400273.

[4] Baran, D.; Malinowski, M. Red-Shifted Glycoporphyrins: Synthesis via Sonogashira Cross-Coupling and Studies on Reactive Oxygen Species Production. *Org. Lett.*, **2025**. https://doi.org/10.1021/acs.orglett.5c02038.

[5] Liu, B.; Liu, D.; Rong, X.; Lu, X.; Fu, Y.; Liu, Q. Ligand-Controlled Stereoselective Synthesis of 2-Deoxy-β-C-glycosides by Cobalt Catalysis. *Angew. Chem. Int. Ed.*, **2023**, *62* (22). https://doi.org/10.1002/anie.202218544.

6. Reactive oxygen species generation plots of new glycoporphyrins

ROS generation plot of **4a** irradiated with 740 nm light

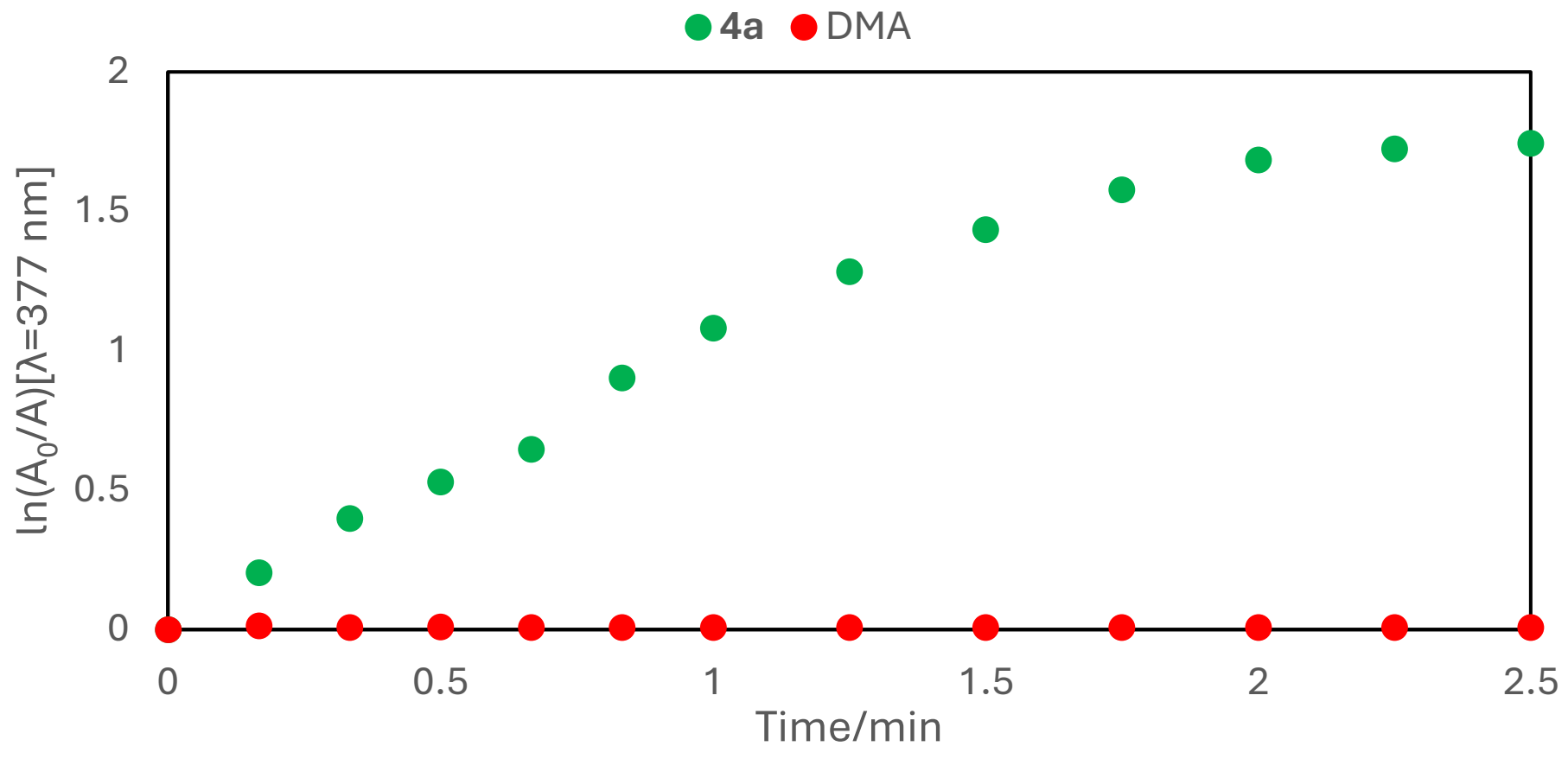

ROS generation plot of **4b** irradiated with 740 nm light

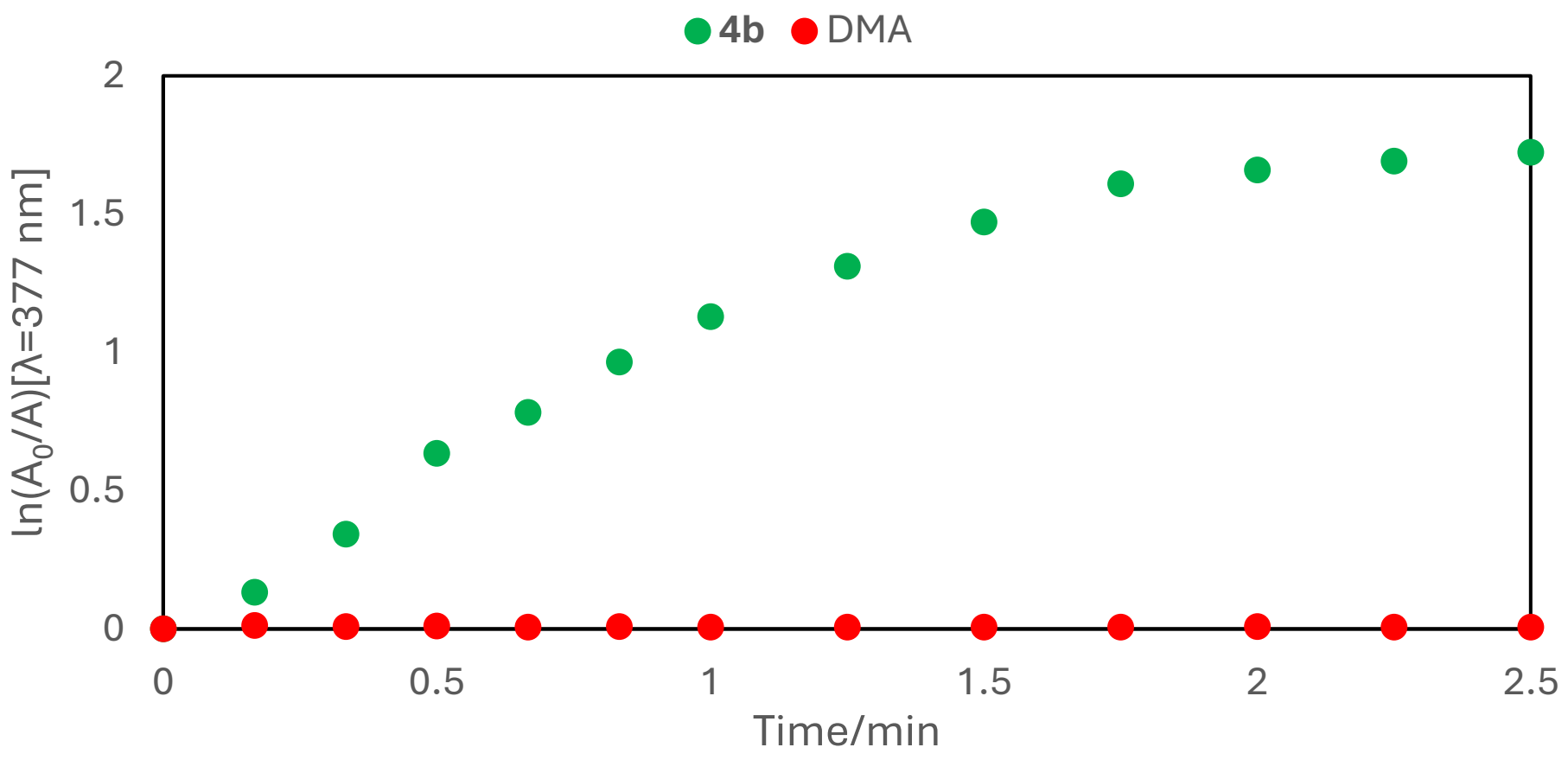

ROS generation plot of **4c** irradiated with 740 nm light

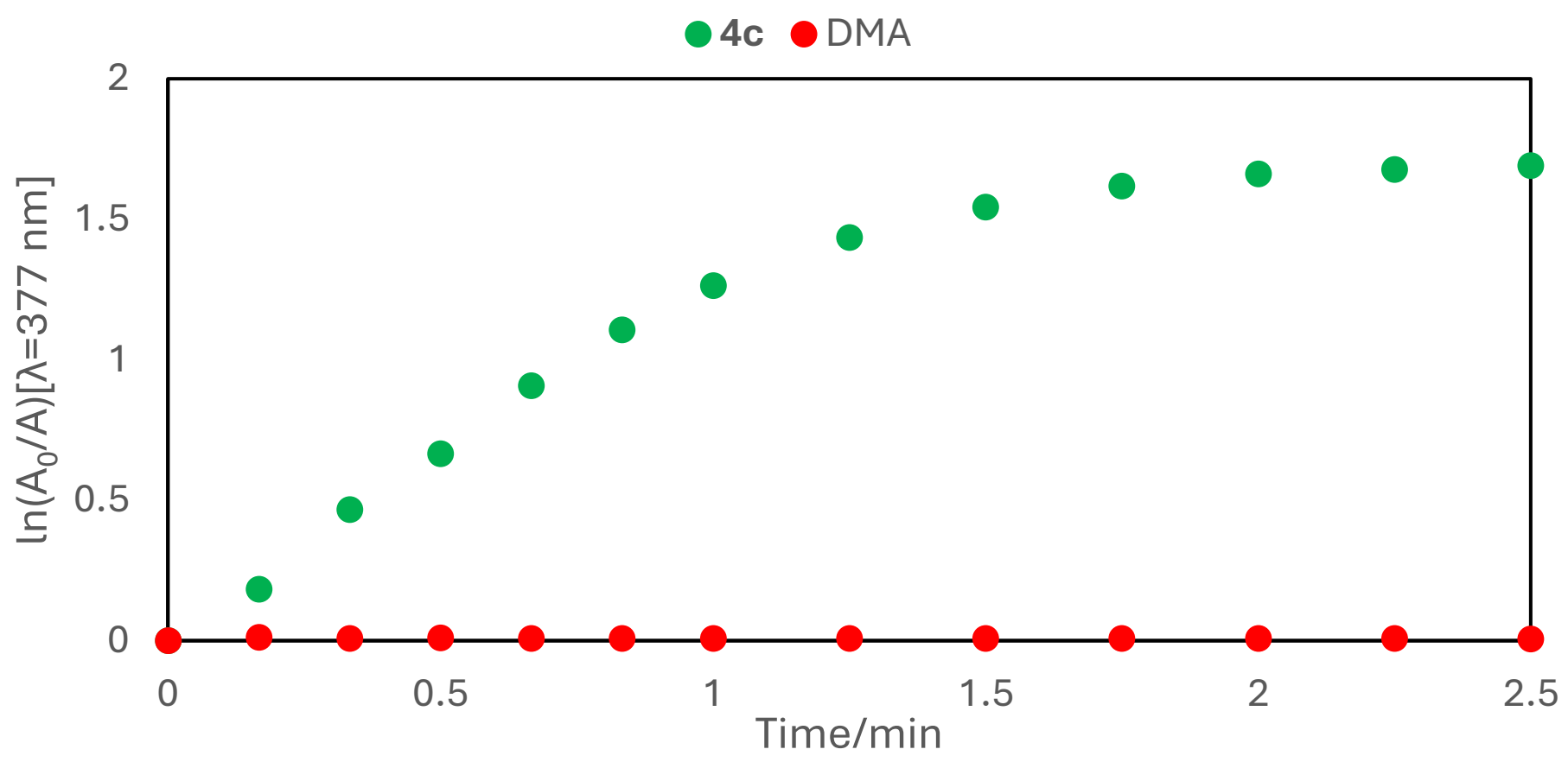

ROS generation plot of **4d** irradiated with 740 nm light

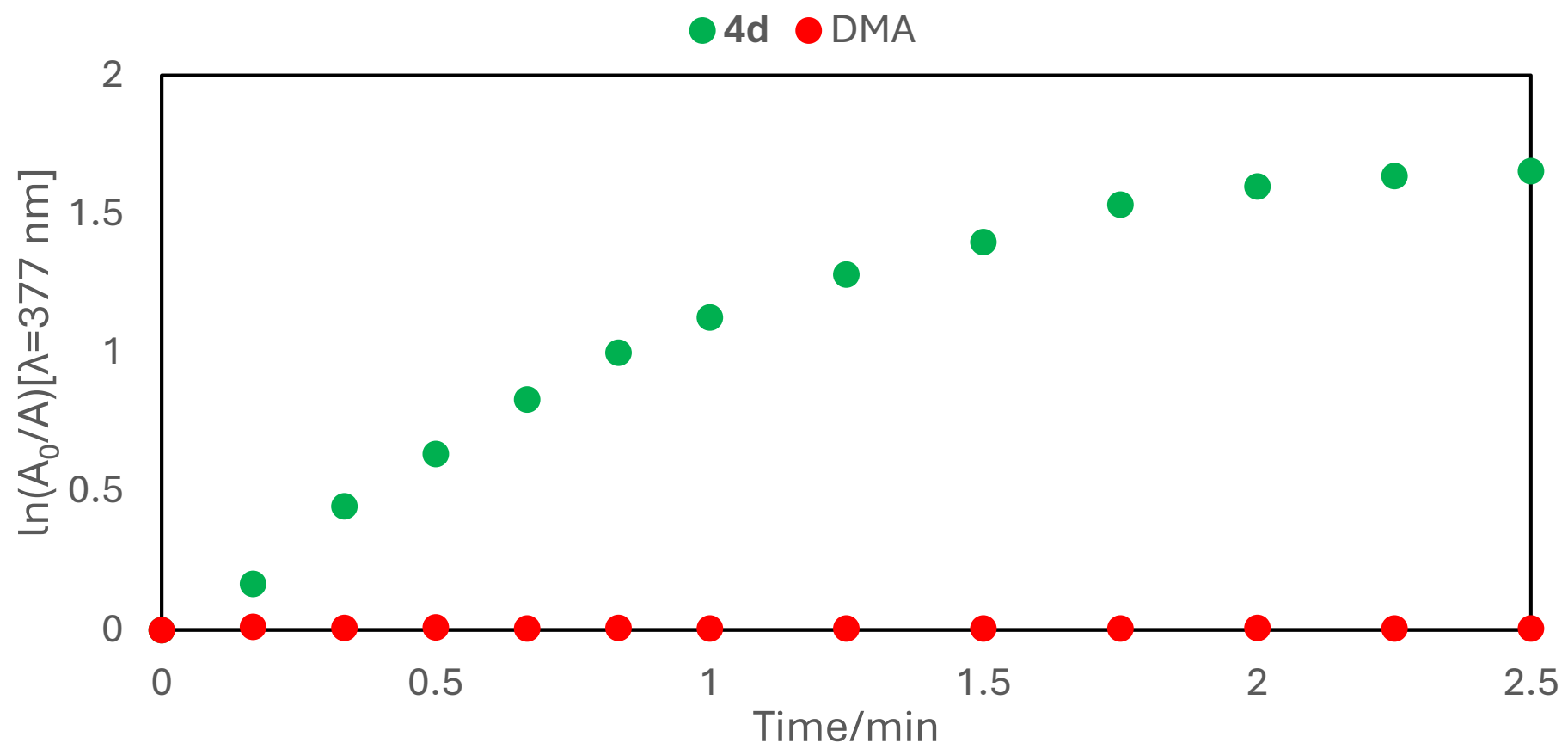

ROS generation plot of **4e** irradiated with 740 nm light

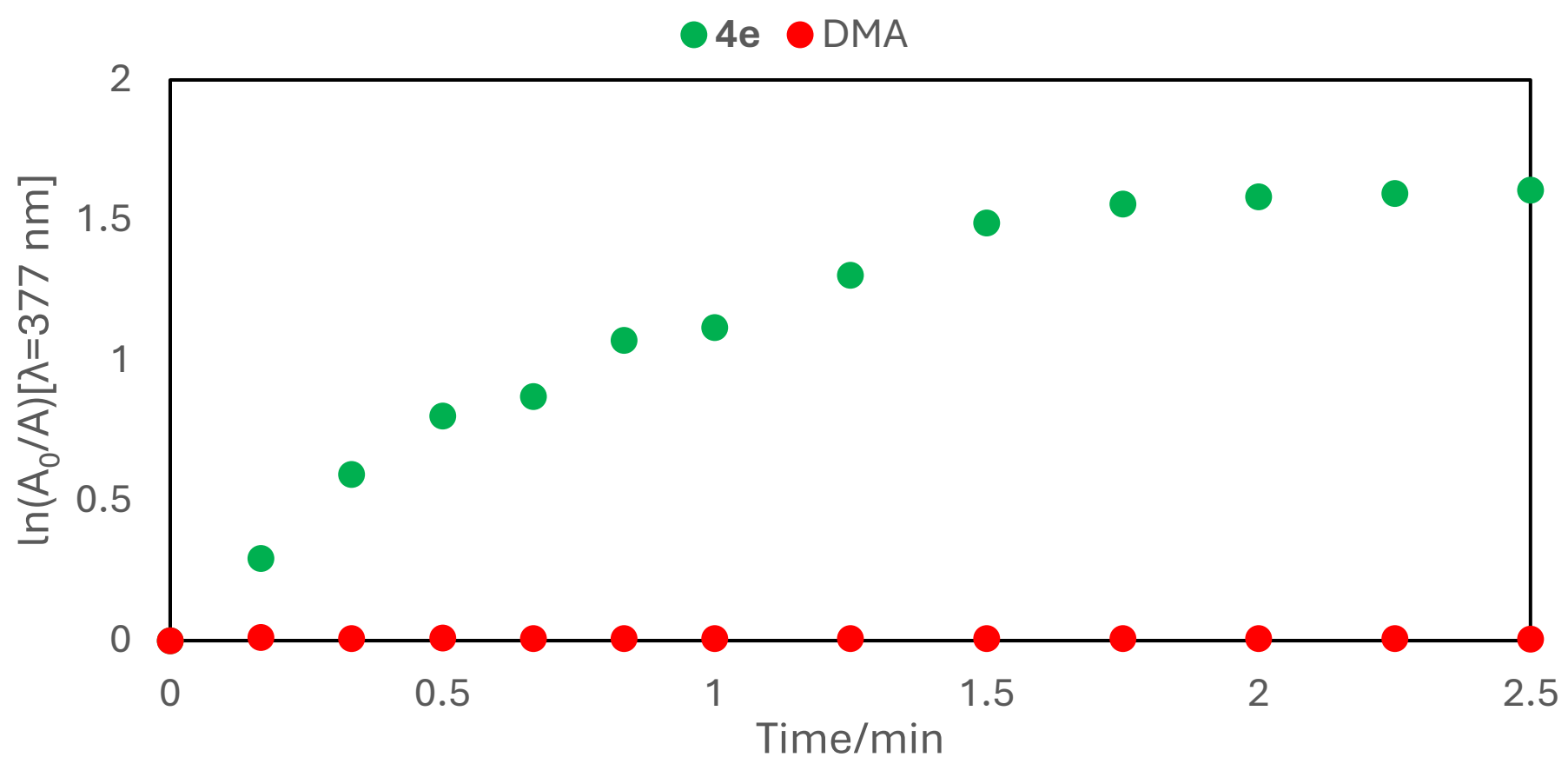

ROS generation plot of **6a** irradiated with 740 nm light

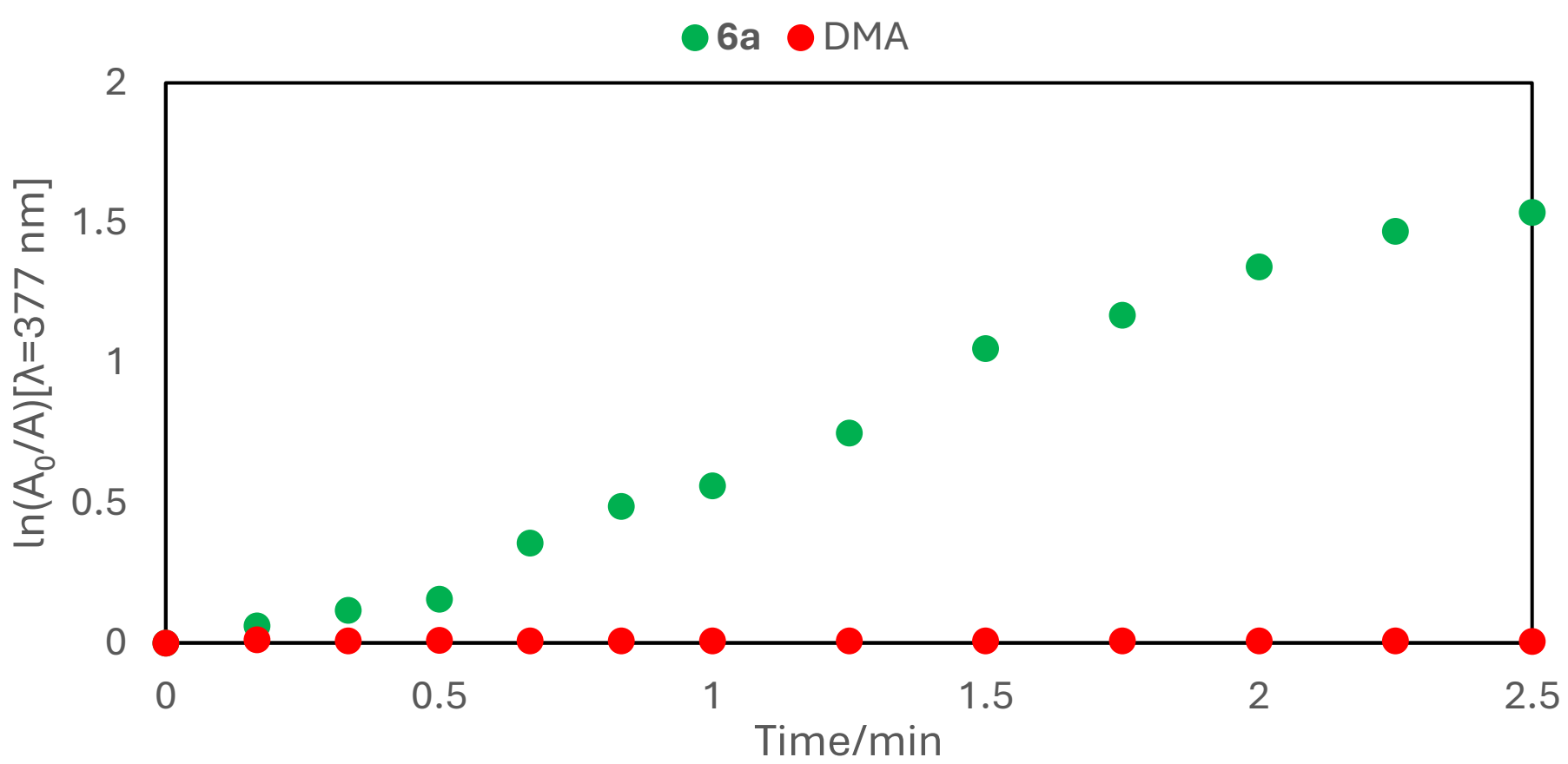

7. UV-vis spectra of porphyrin compounds

UV-vis absorbance spectrum of 5a in $CHCl_3$

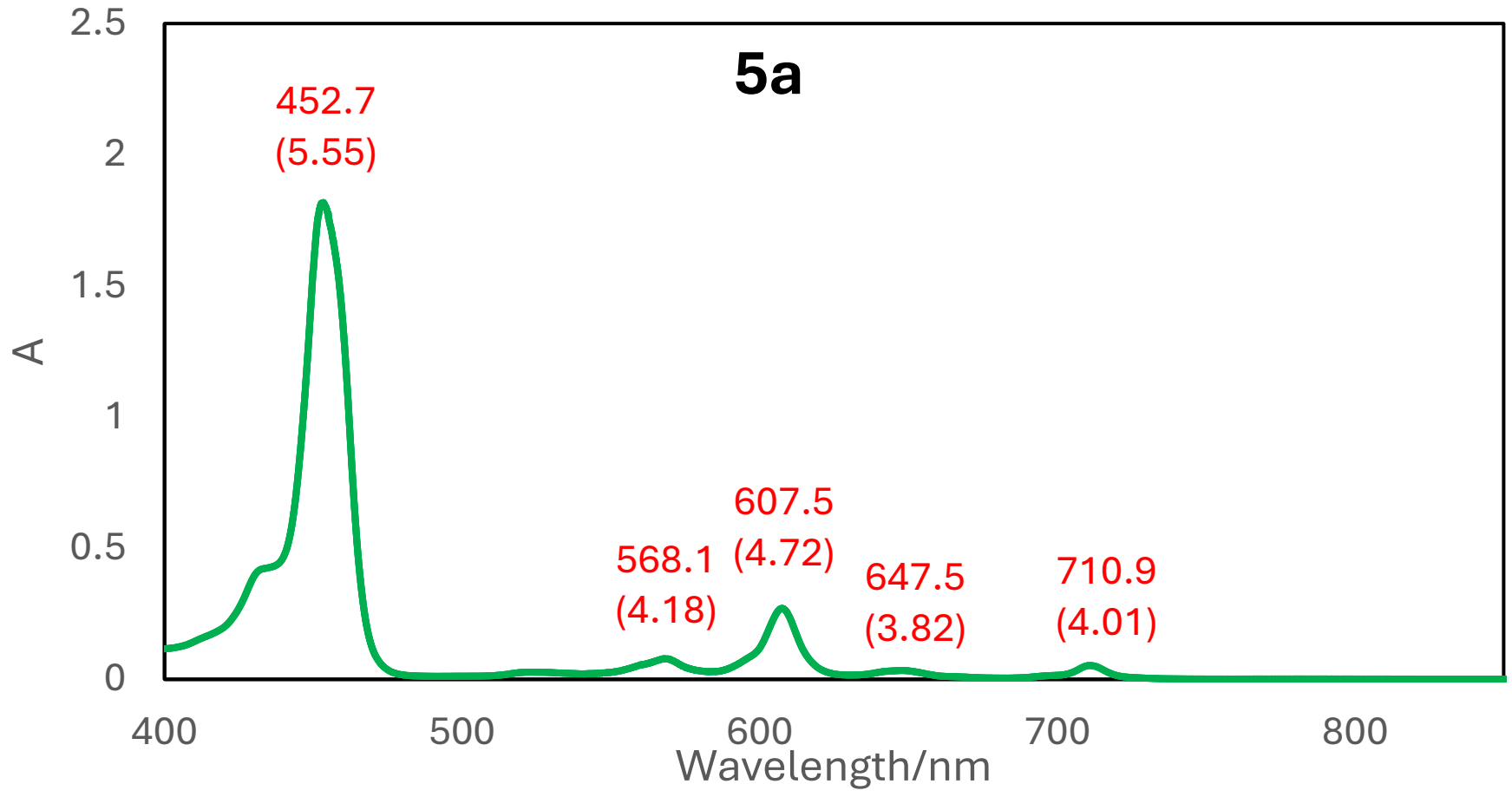

UV-vis absorbance spectrum of 4a in $CHCl_3$

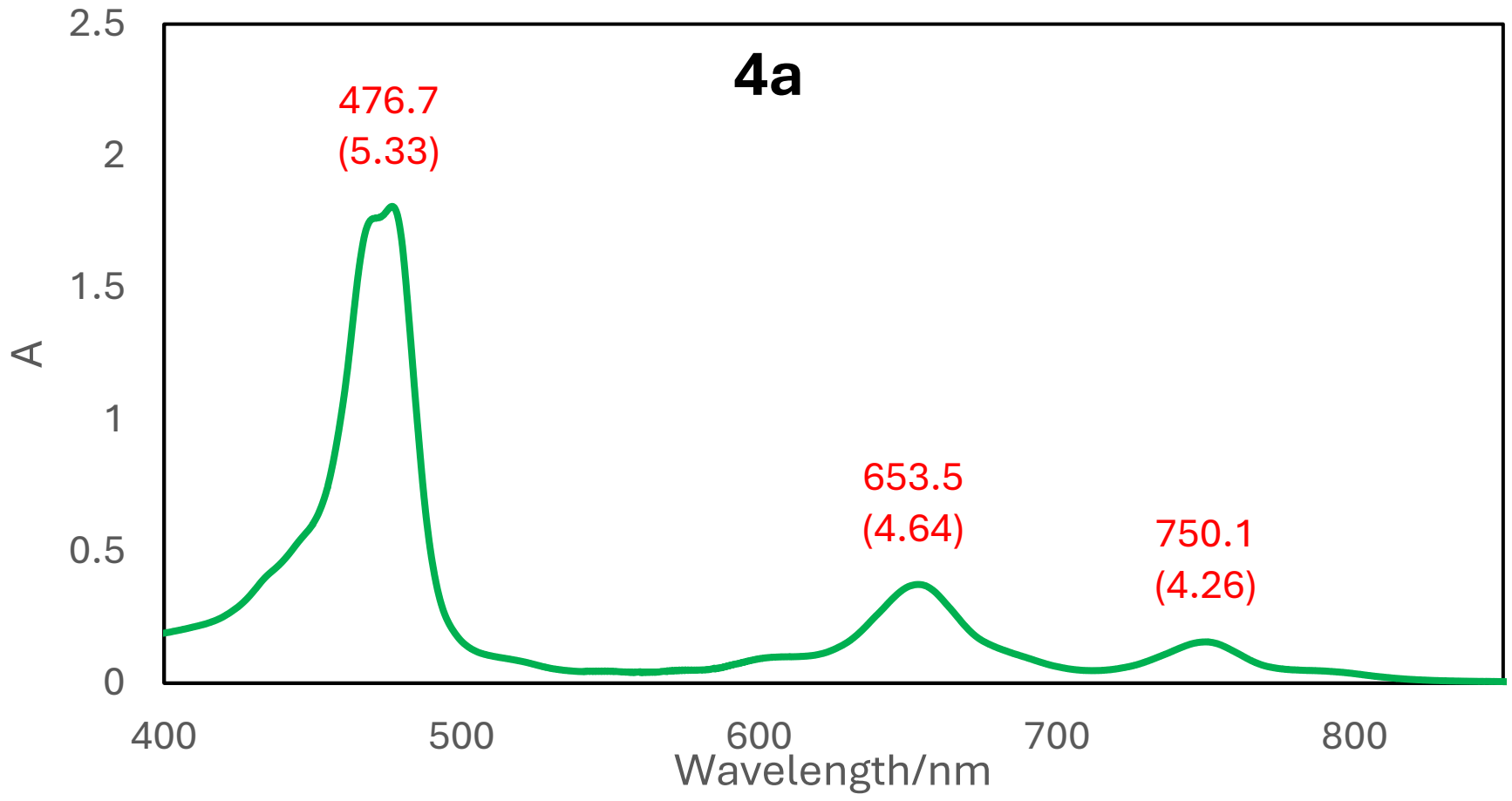

UV-vis absorbance spectrum of 4b in $CHCl_3$

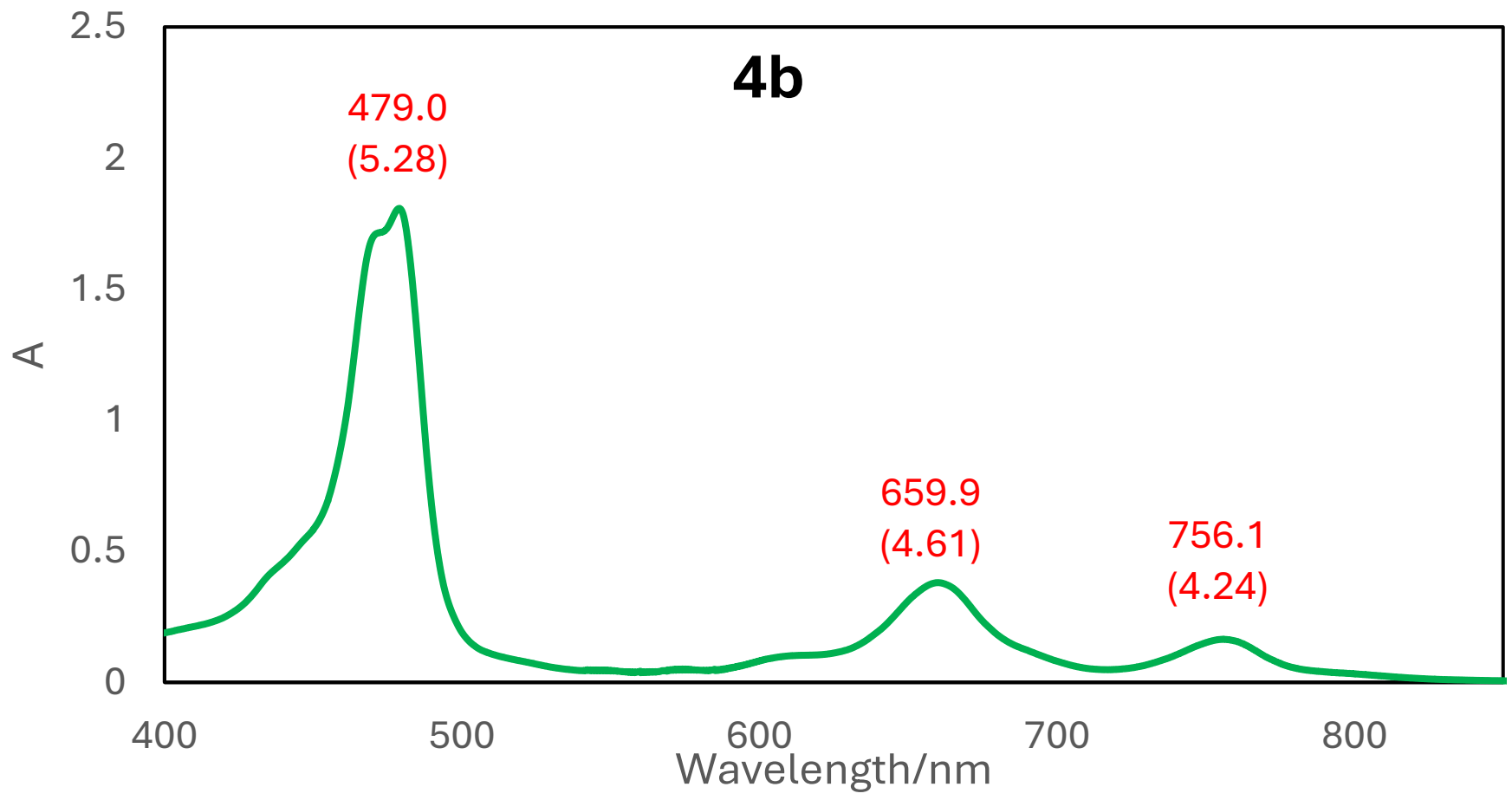

UV-vis absorbance spectrum of 4c in $CHCl_3$

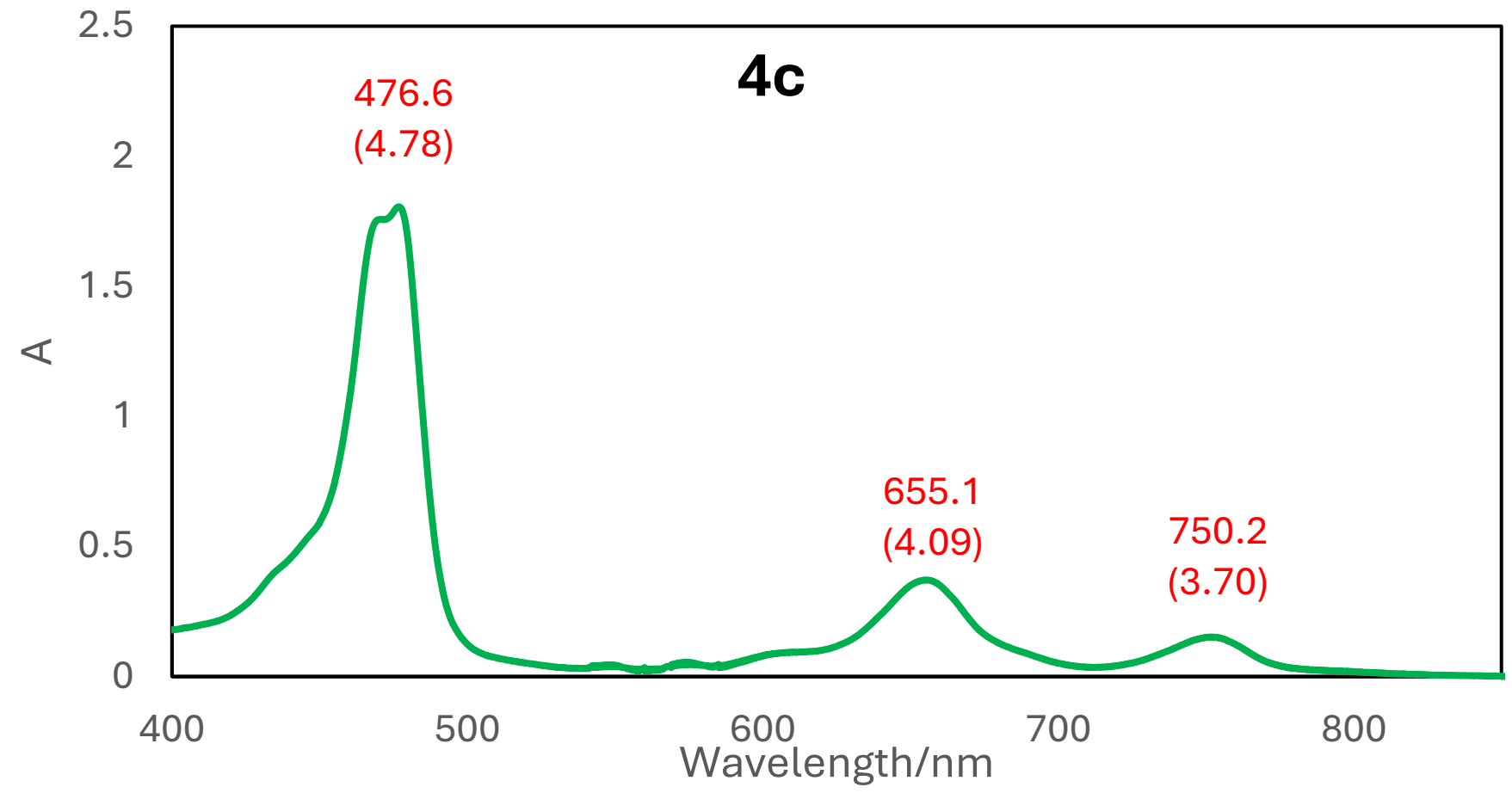

UV-vis absorbance spectrum of 4d in $CHCl_3$

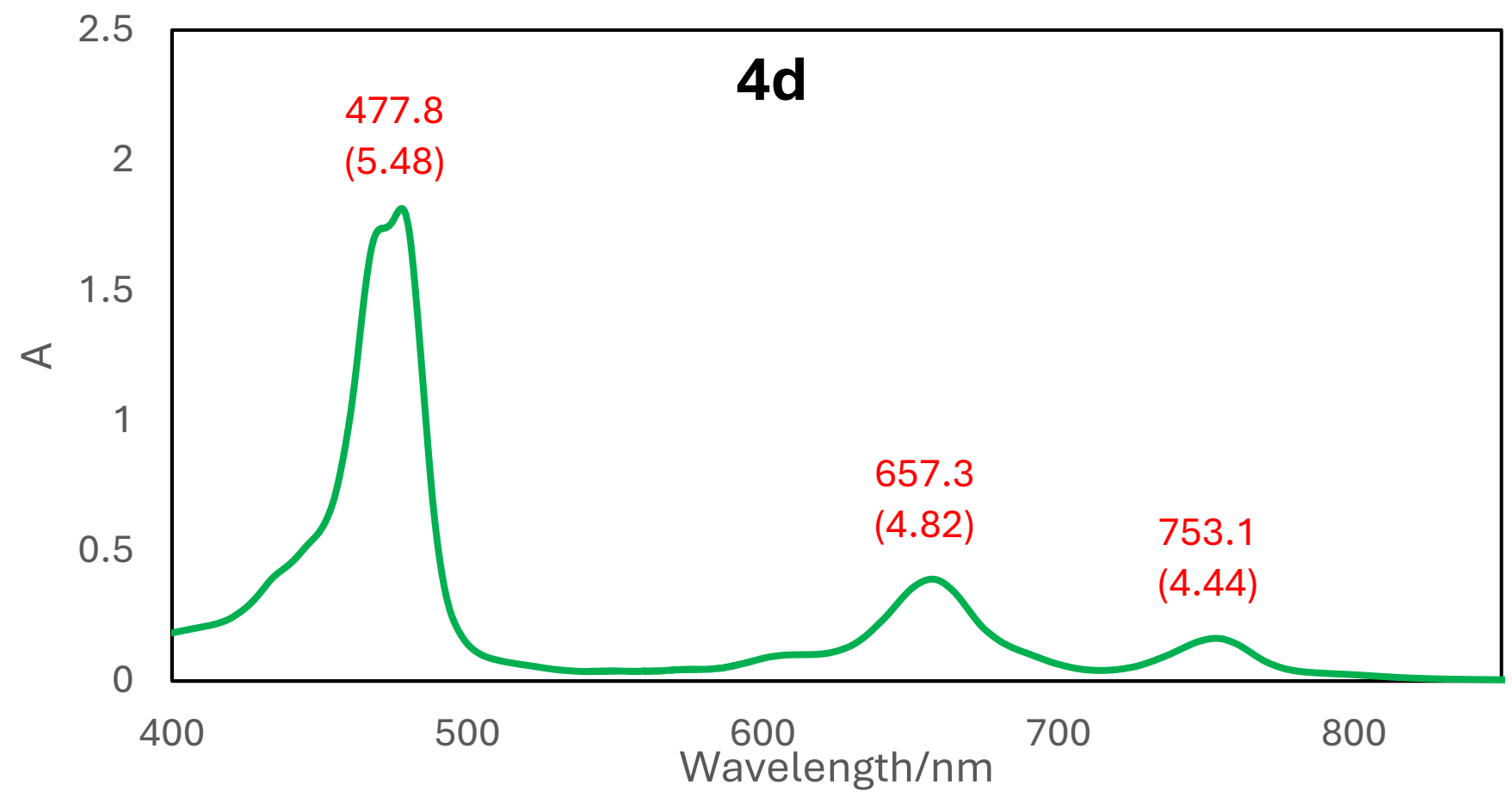

UV-vis absorbance spectrum of 4e in $CHCl_3$

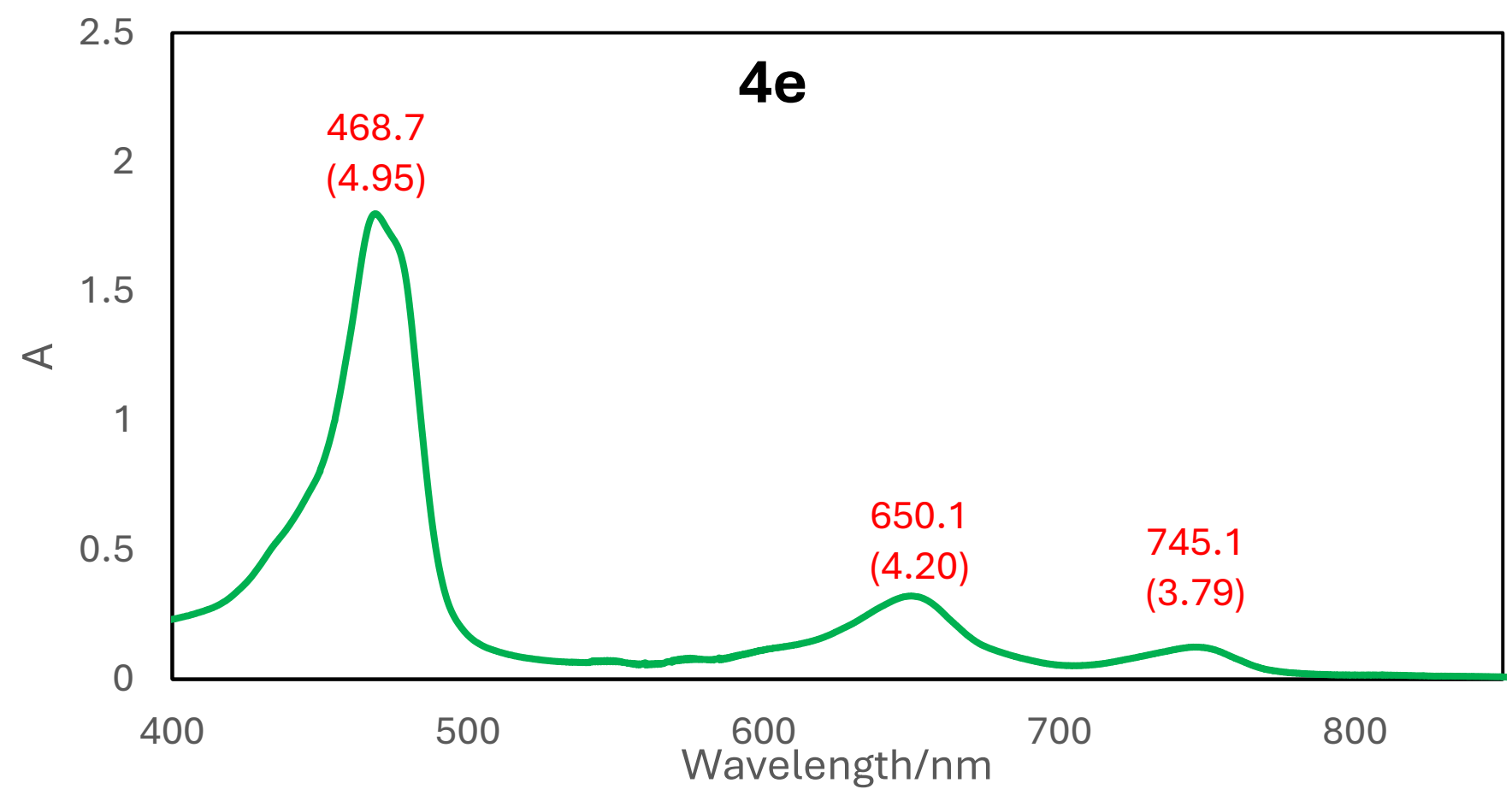

UV-vis absorbance spectrum of 6a in DMF

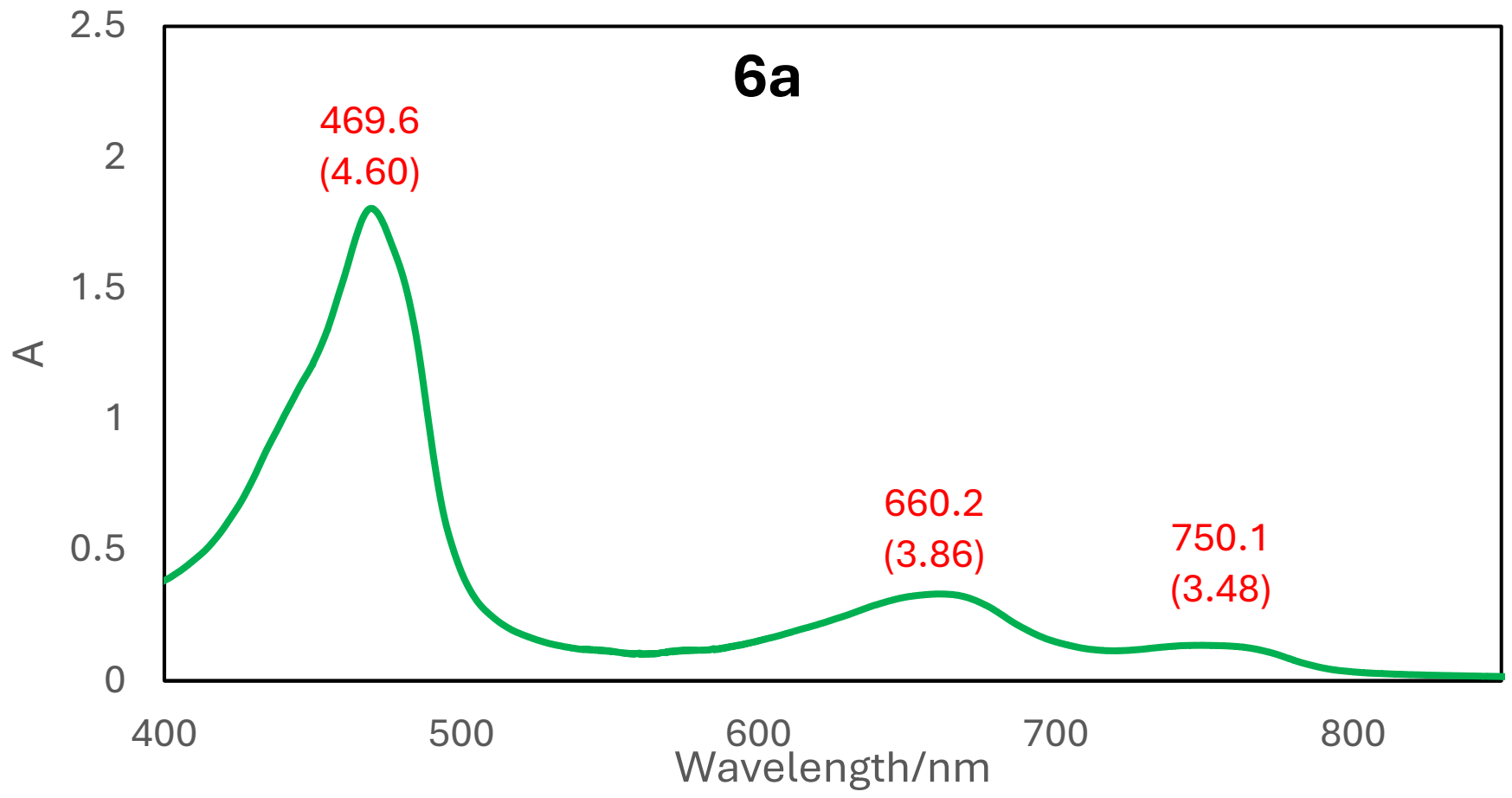

8. NMR spectra of known compounds

^1H NMR (600 MHz, $CDCl_3$) of $Pd(PPh_3)_2Cl_2$

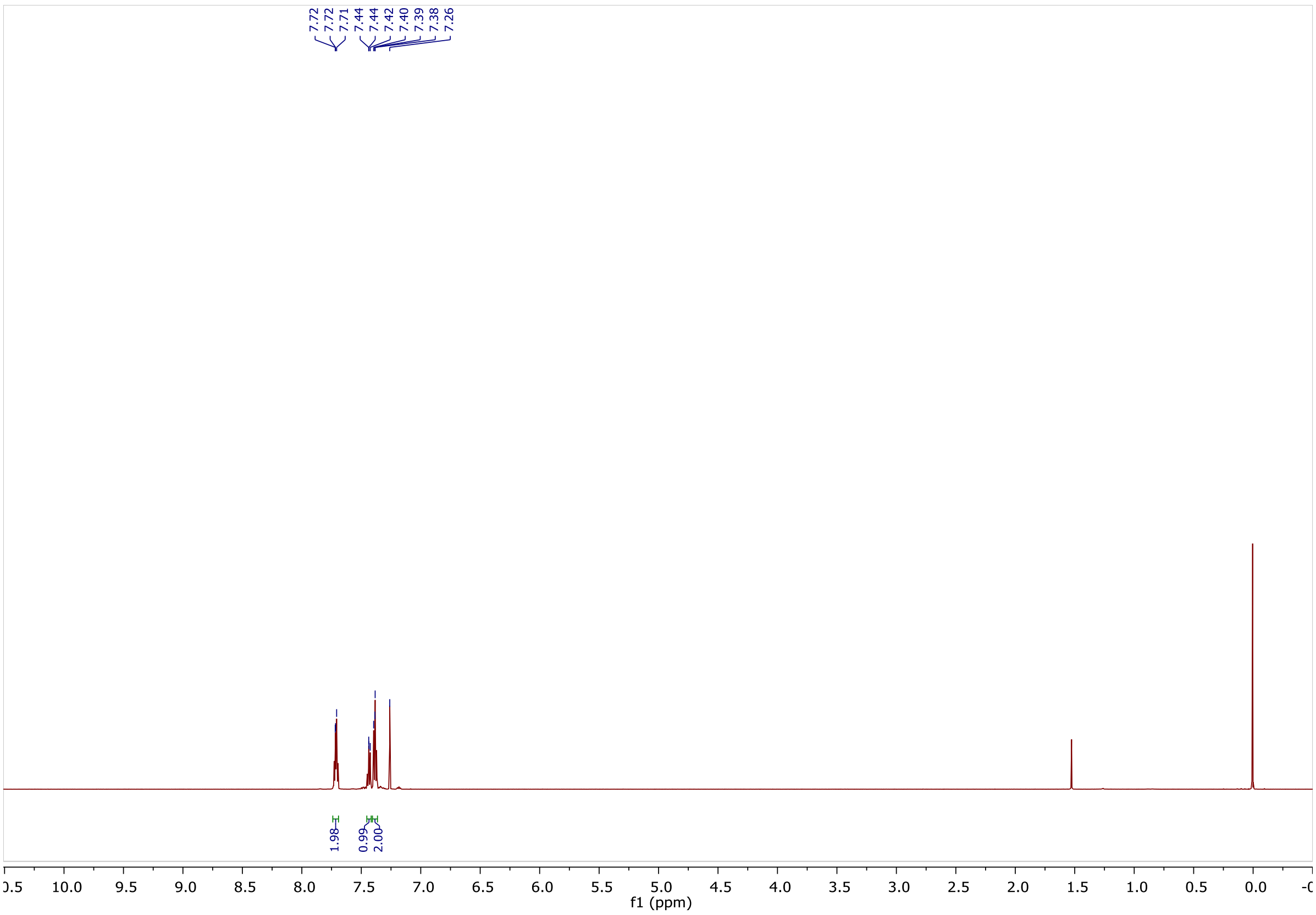

^1H NMR (600 MHz, $CDCl_3$) of 5a

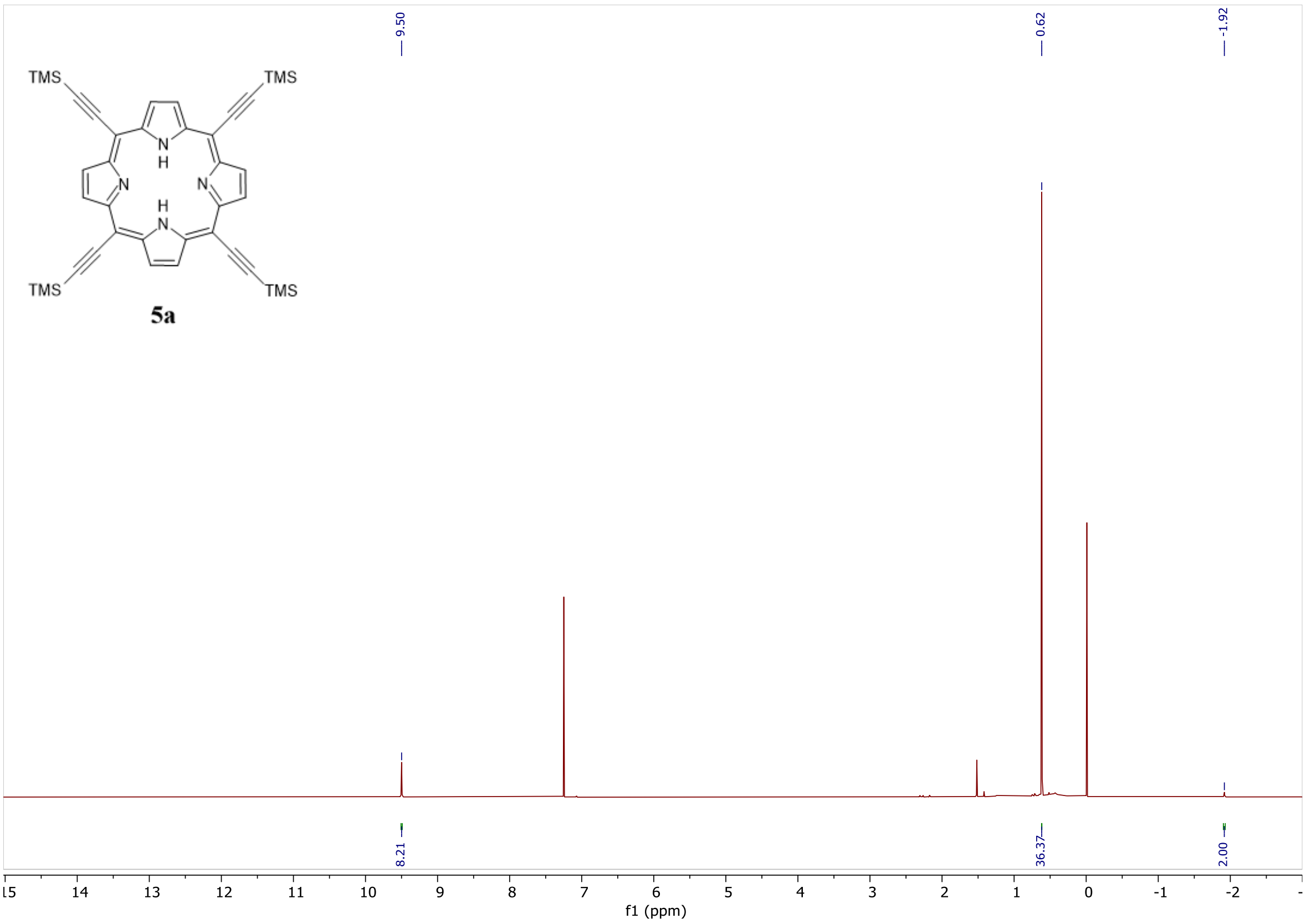

9. NMR spectra of new compounds

^{1}H NMR (600 MHz, CDCl$_3$) of 2a

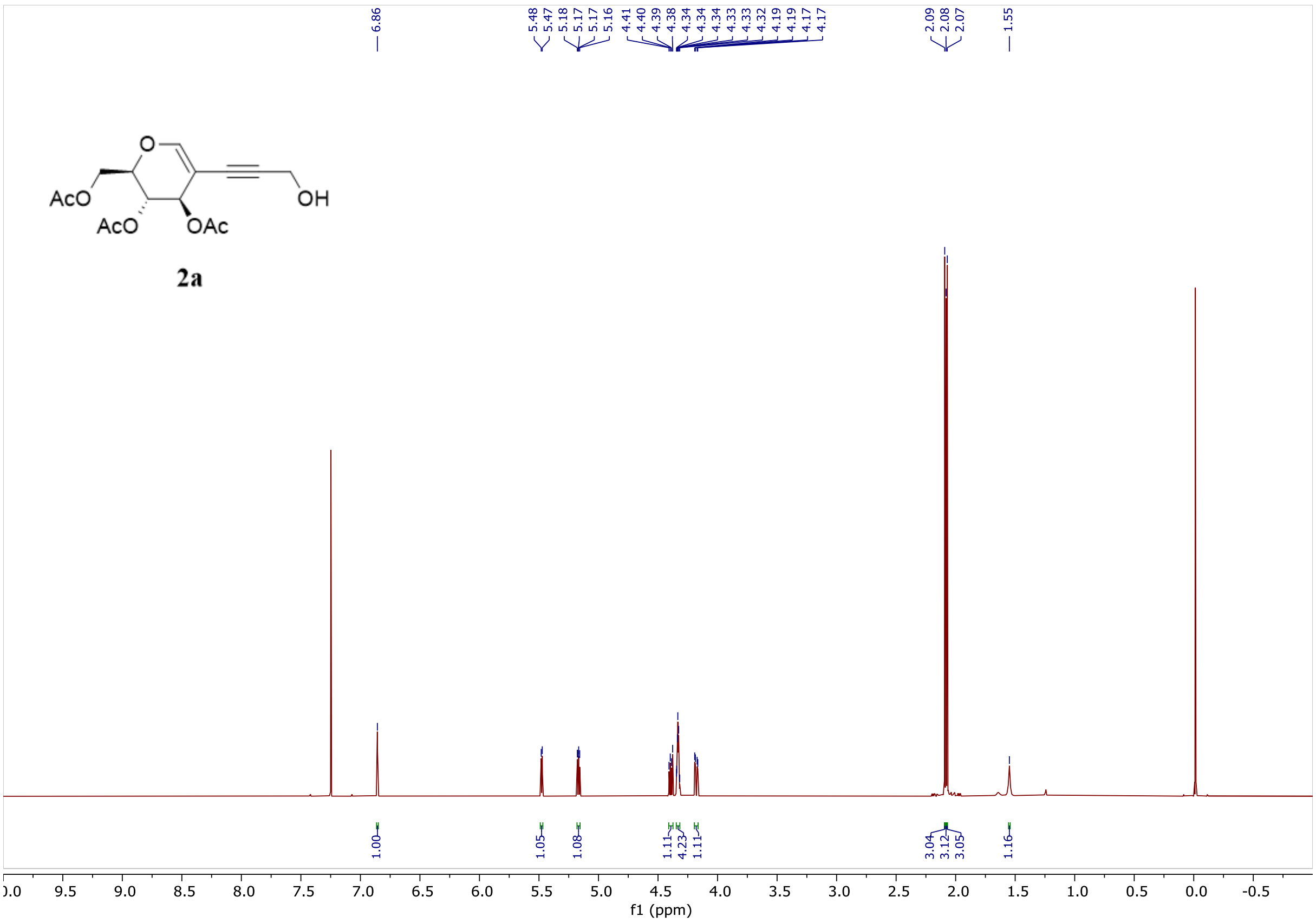

^{13}C{^{1}H} NMR (151 MHz, CDCl$_3$) of 2a

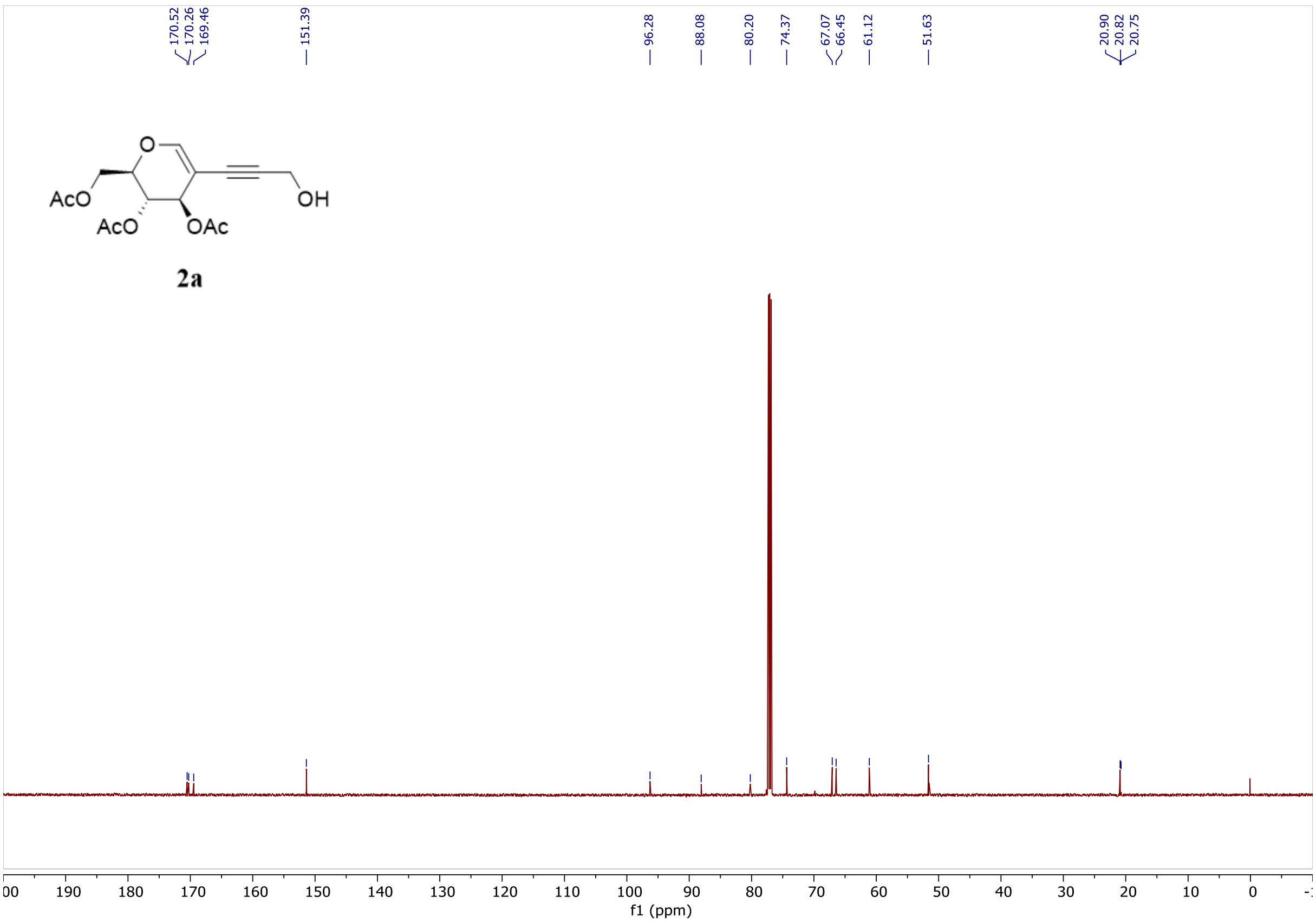

^{1}H NMR (600 MHz, $CDCl_3$) of 3a

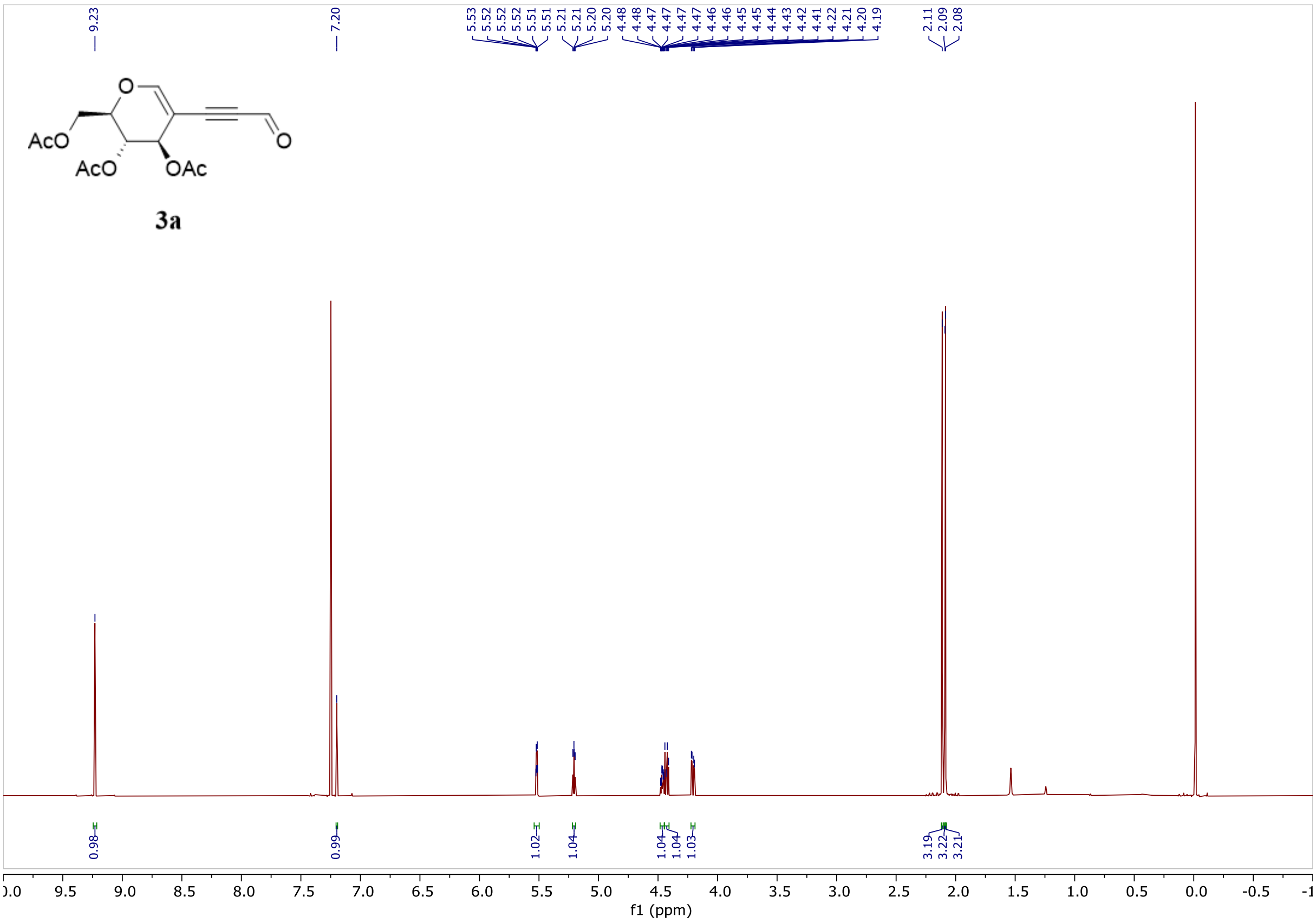

$^{13}C\{^{1}H\}$ NMR (151 MHz, $CDCl_3$) of 3a

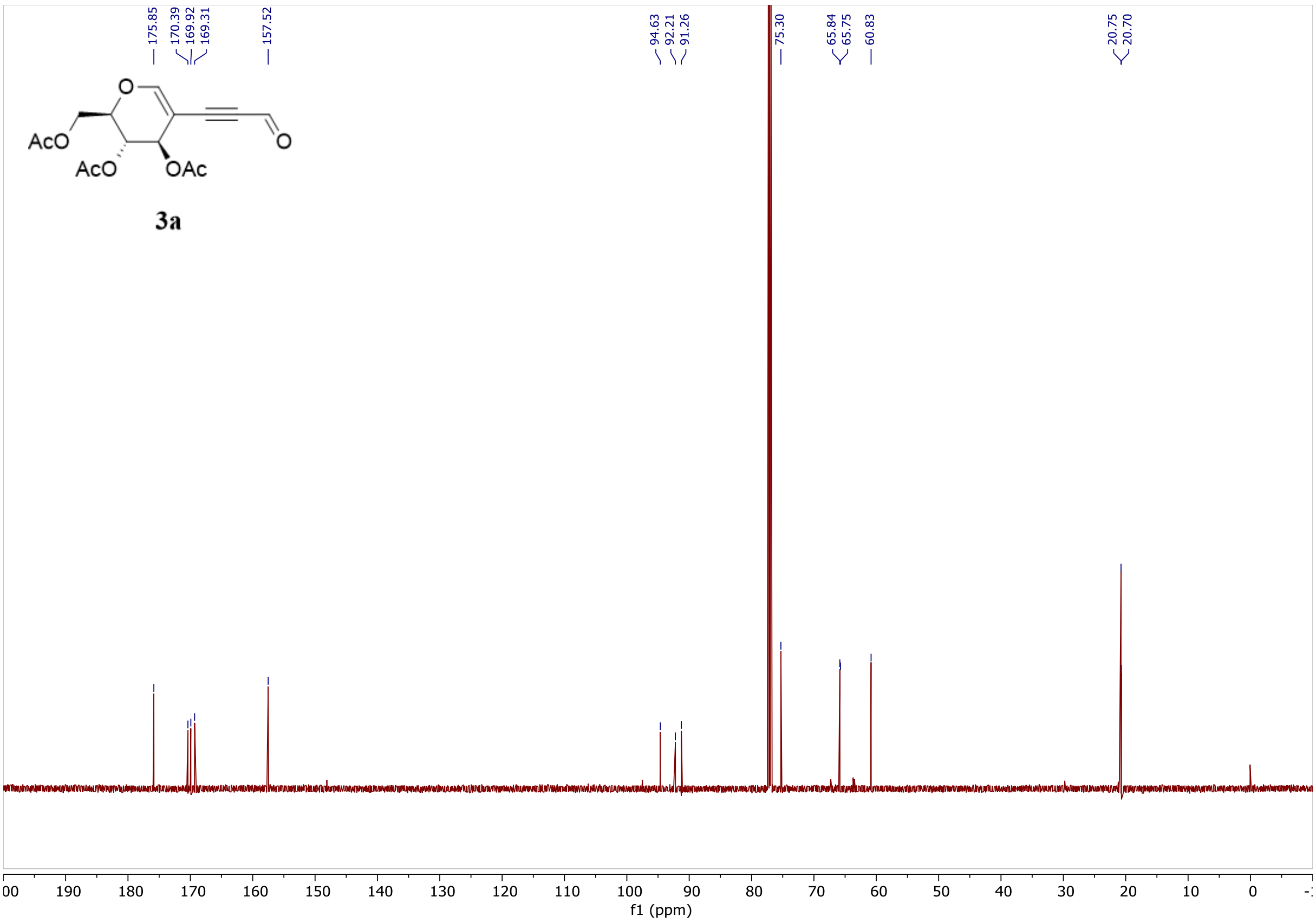

^{1}H NMR (600 MHz, $CDCl_3$) of 4a

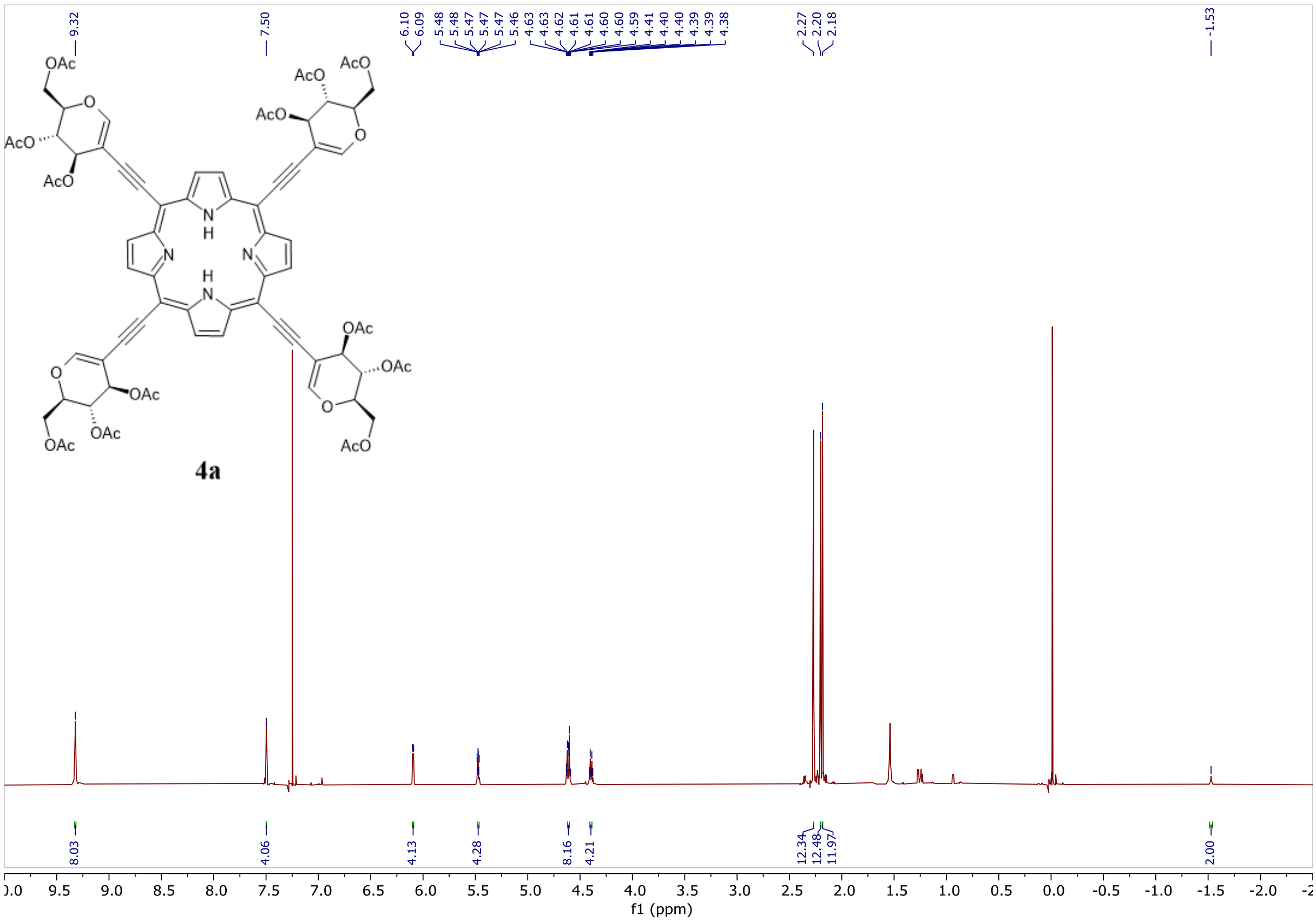

$^{13}C\{^{1}H\}$ NMR (151 MHz, $CDCl_3$) of 4a

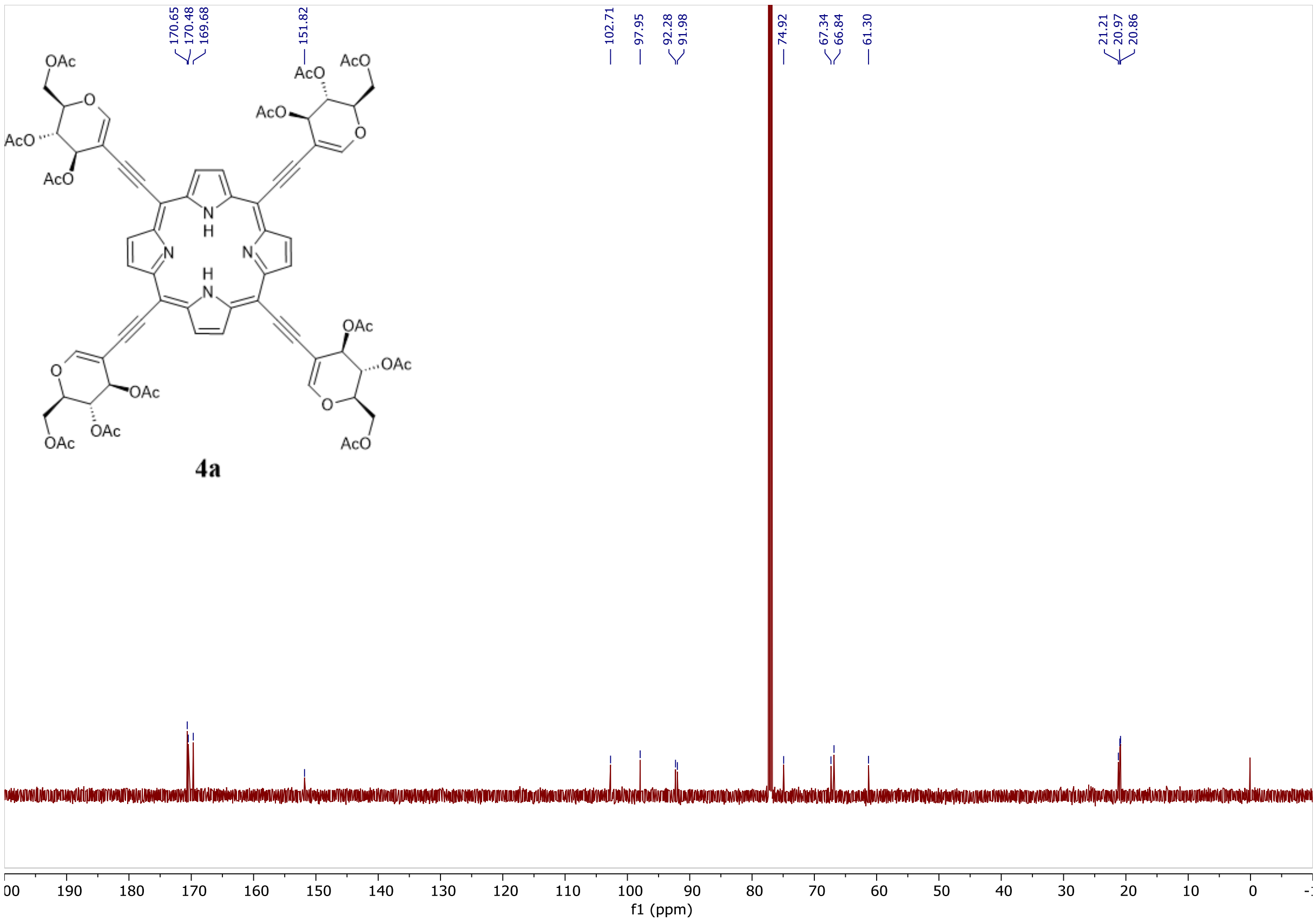

^{1}H NMR (600 MHz, $CDCl_3$) of 4b

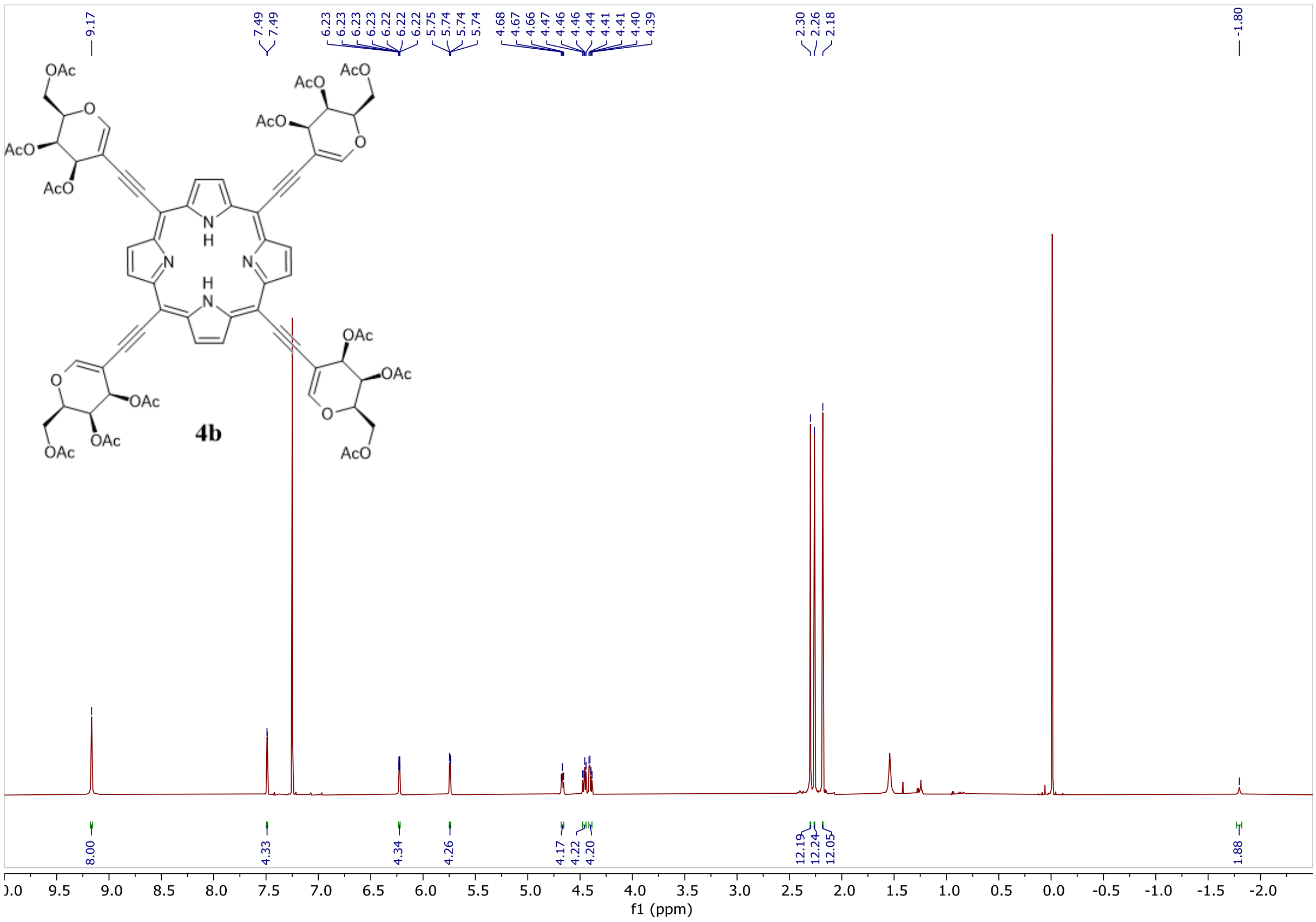

$^{13}C\{^1H\}$ NMR (151 MHz, $CDCl_3$) of 4b

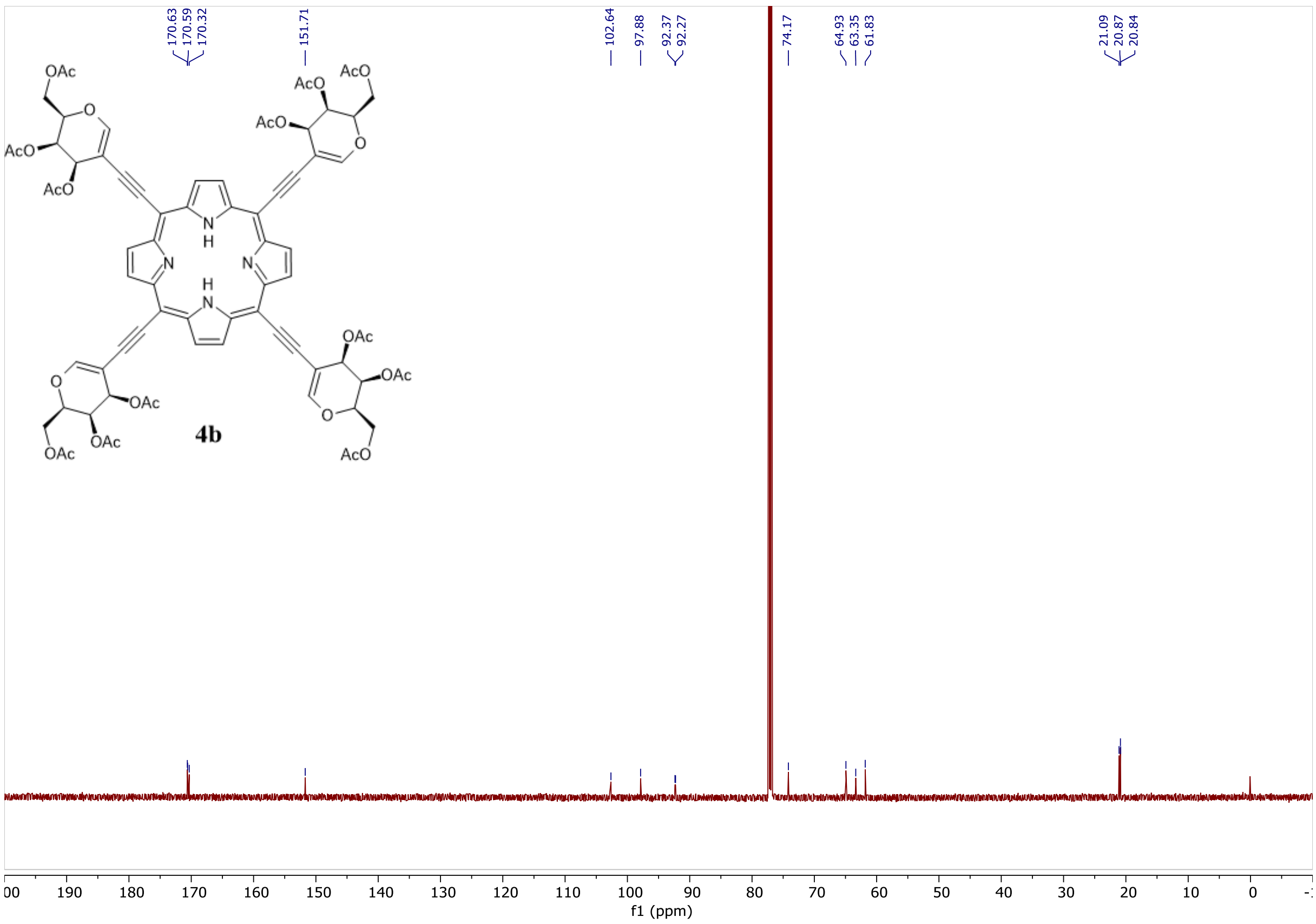

^{1}H NMR (600 MHz, $CDCl_3$) of 4c

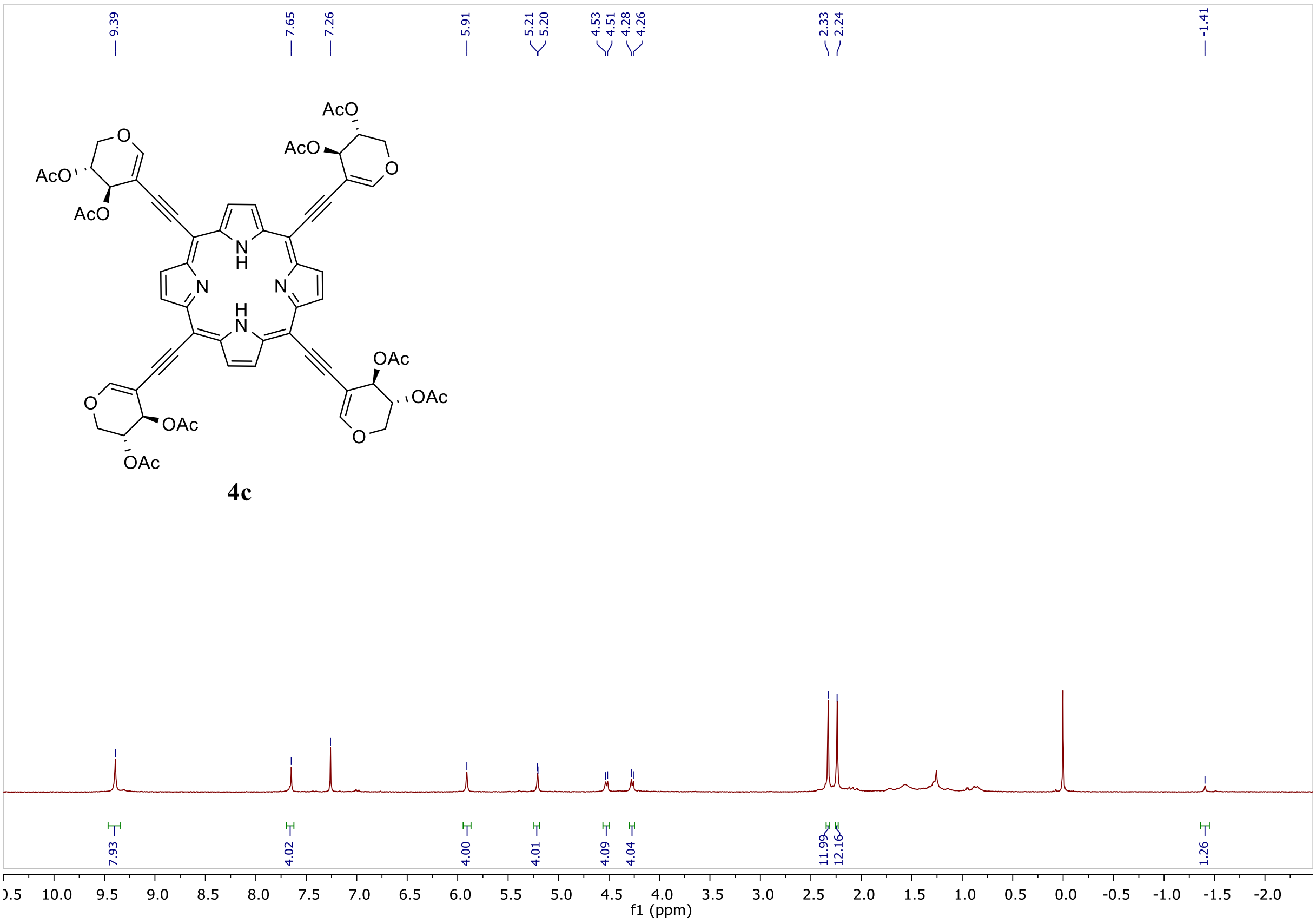

$^{13}C\{^{1}H\}$ NMR (151 MHz, $CDCl_3$) of 4c

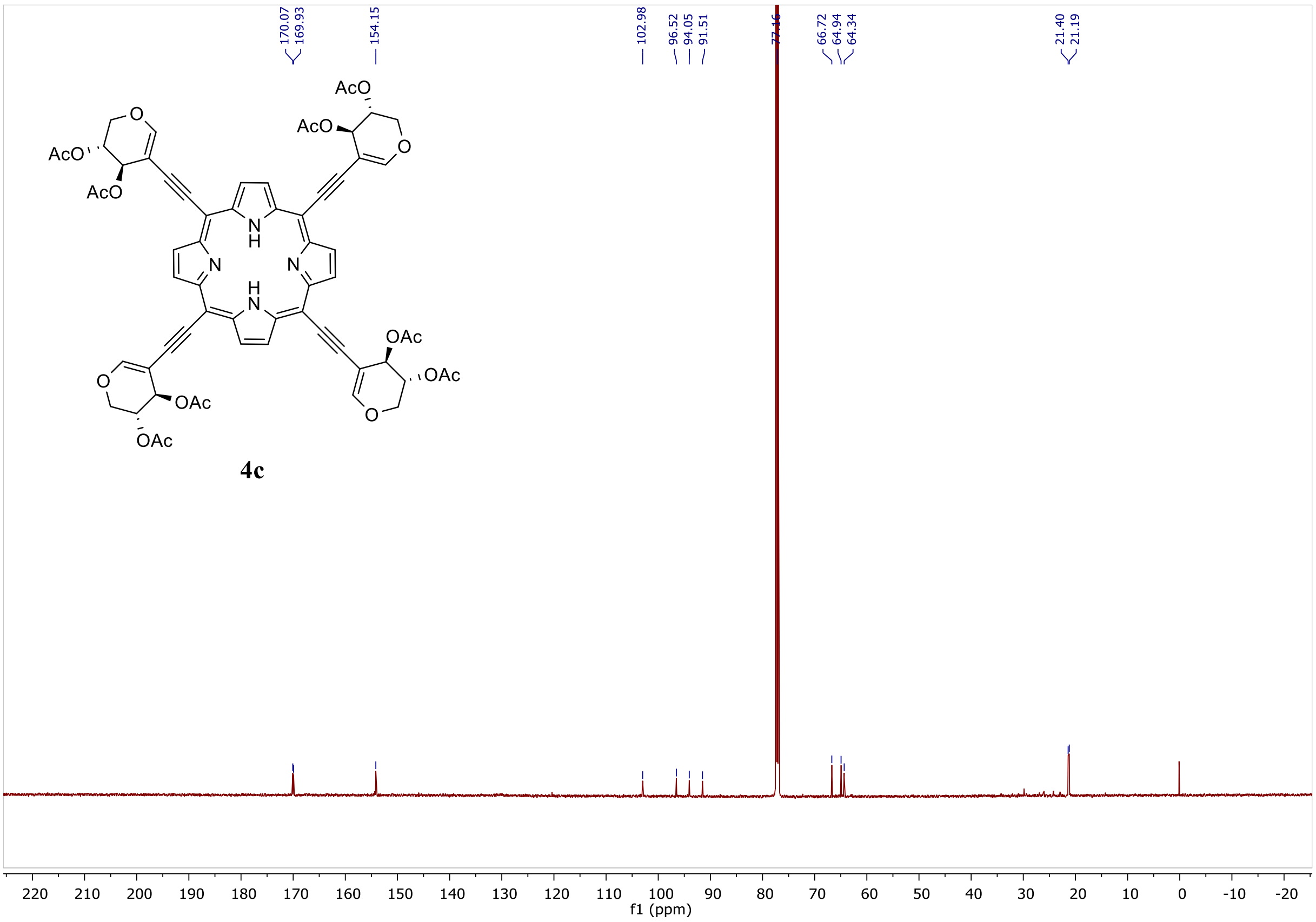

^{1}H NMR (600 MHz, $CDCl_3$) of 4d

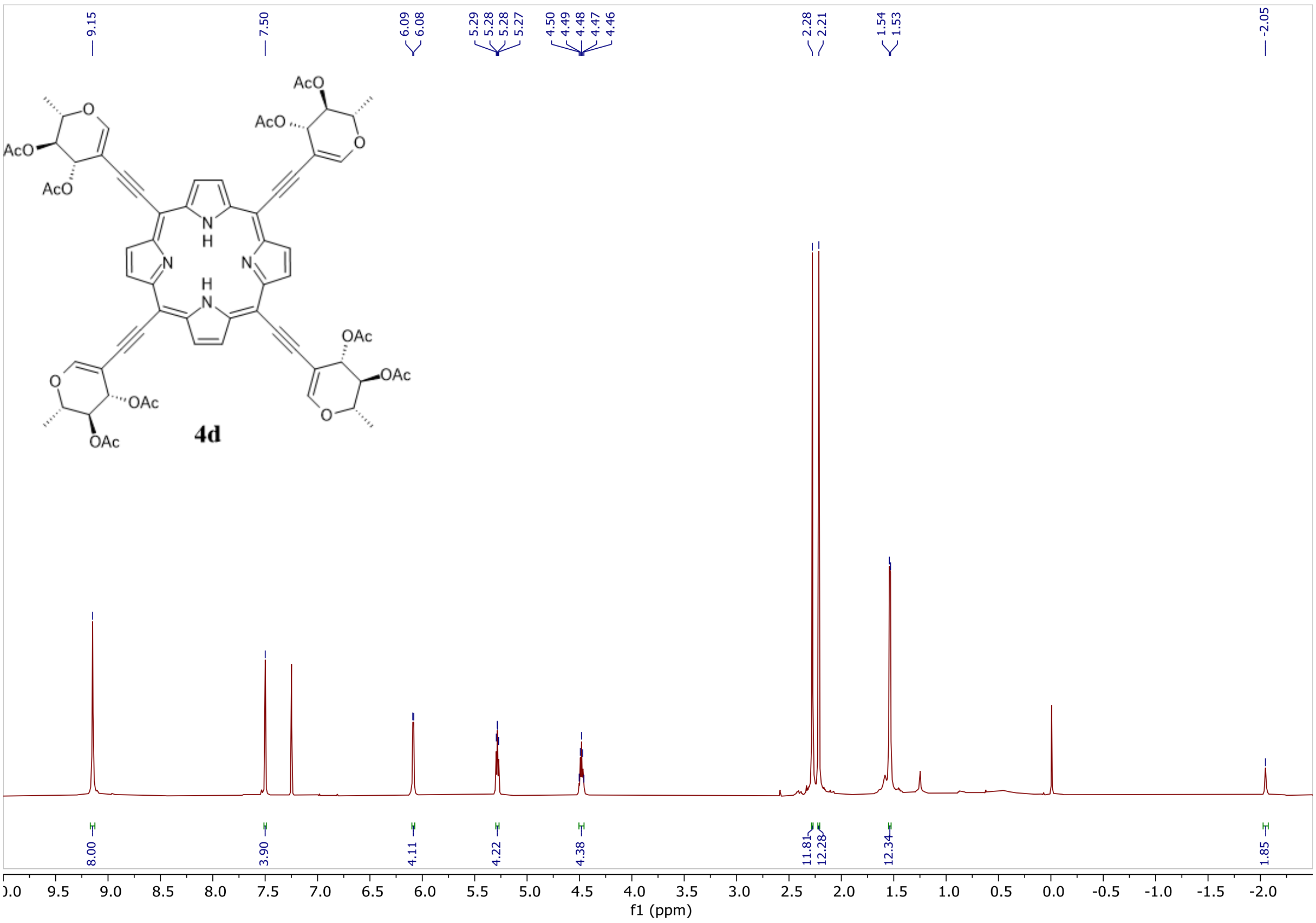

$^{13}C\{^{1}H\}$ NMR (151 MHz, $CDCl_3$) of 4d

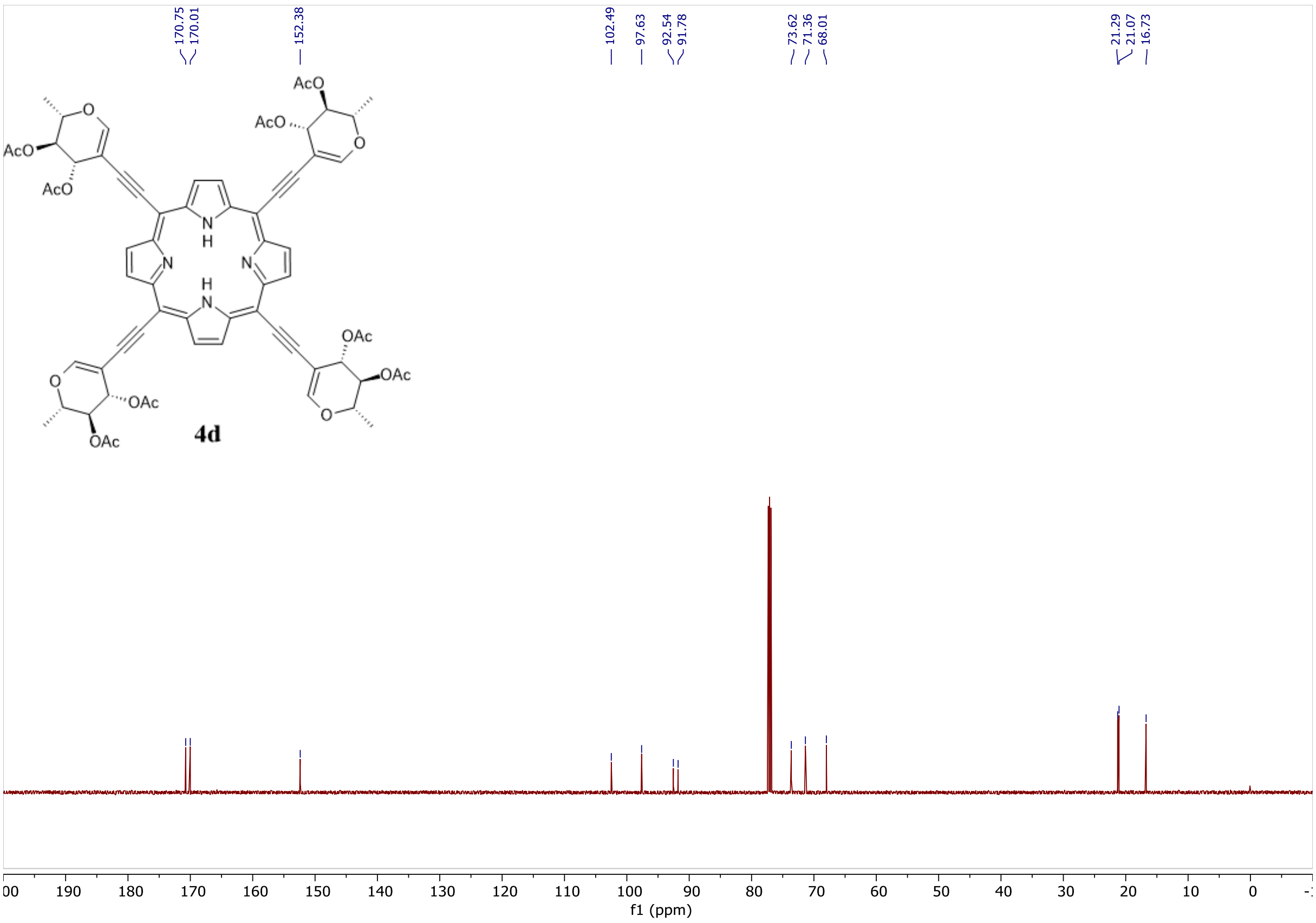

^{1}H NMR (600 MHz, $CDCl_3$) of 4e

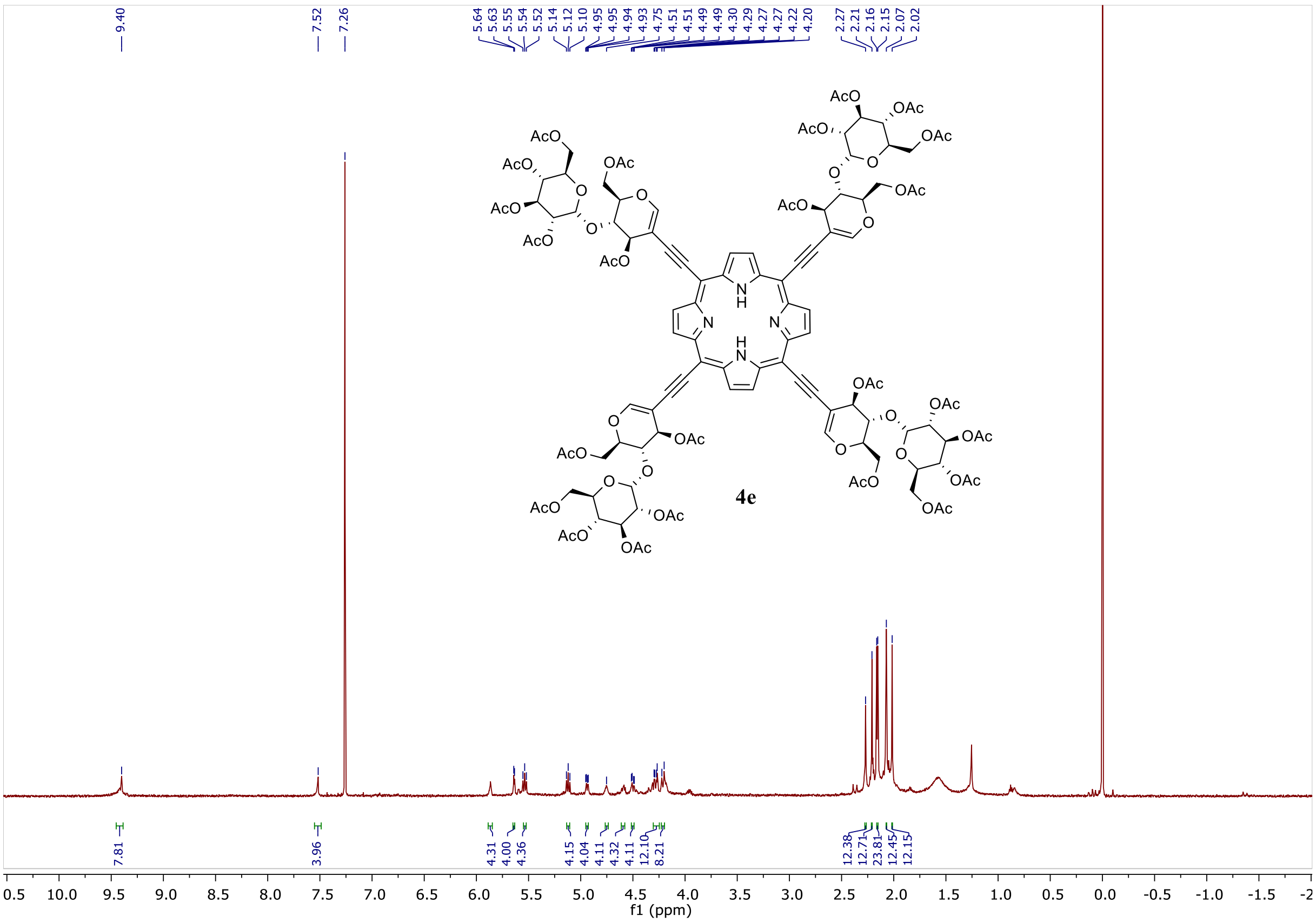

^{13}C{^{1}H} NMR (151 MHz, $CDCl_3$) of 4e

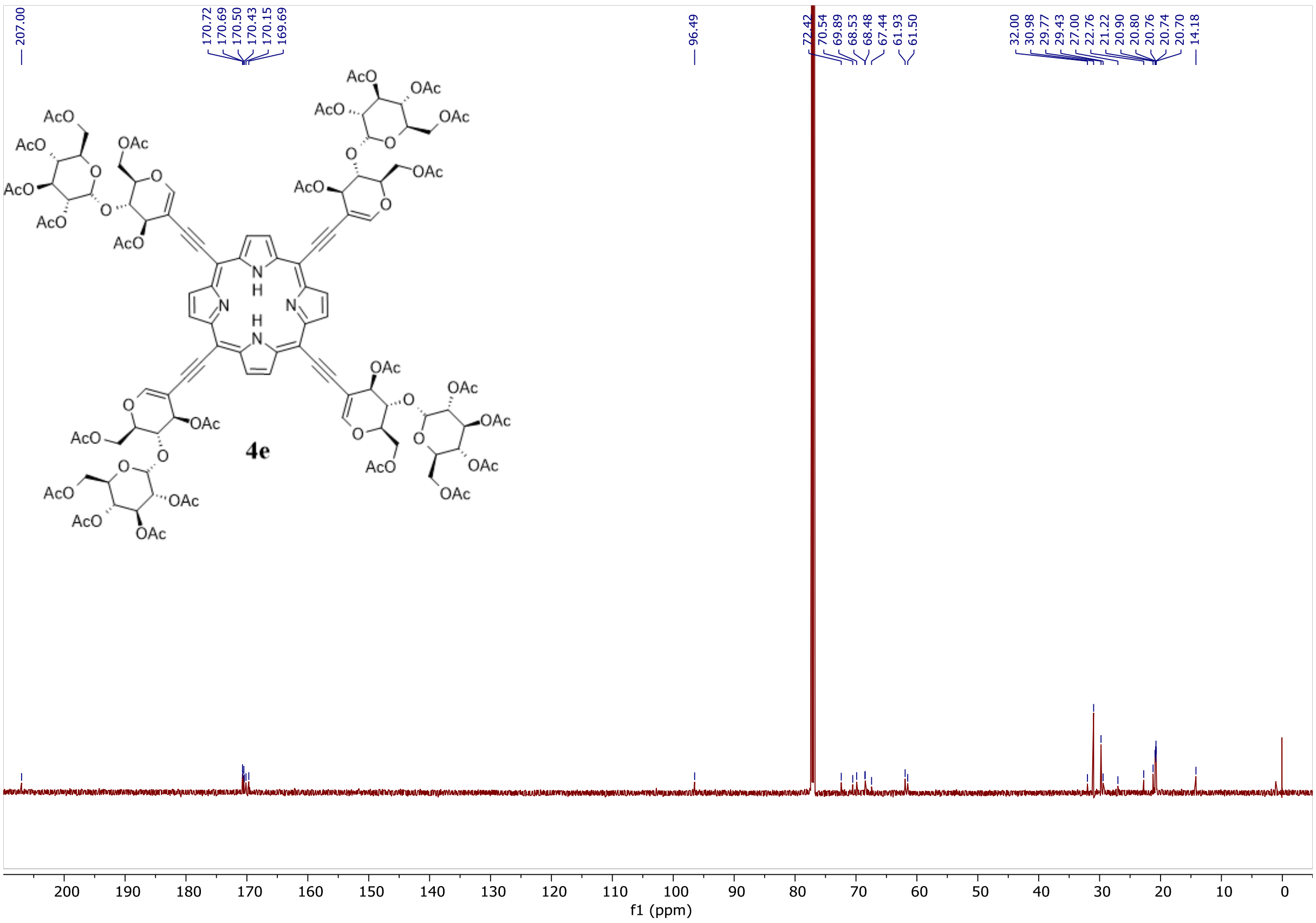

^{1}H NMR (600 MHz, DMSO-d$_6$) of 6a

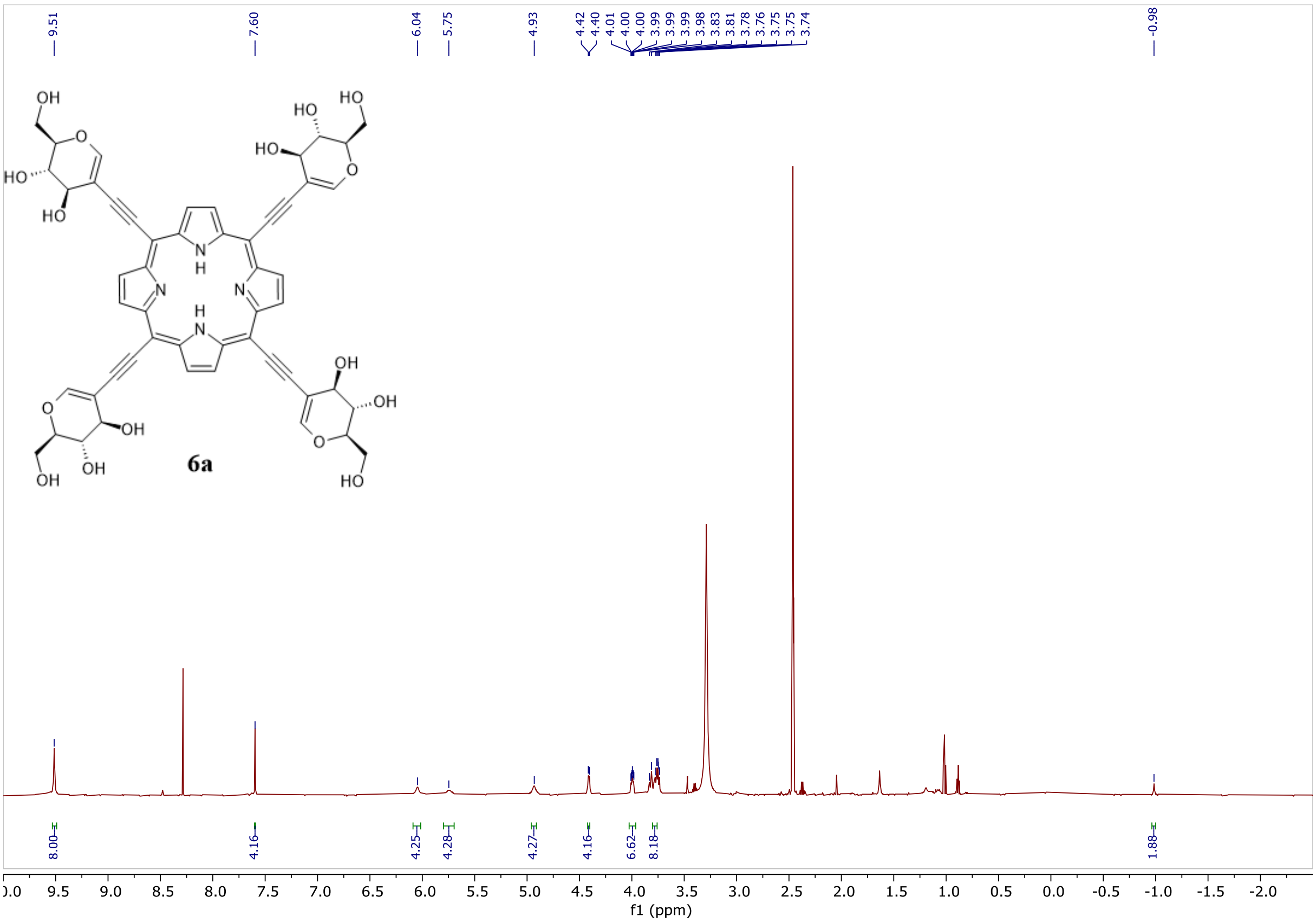

^{13}C NMR (151 MHz, APT, DMSO-d$_6$) of 6a

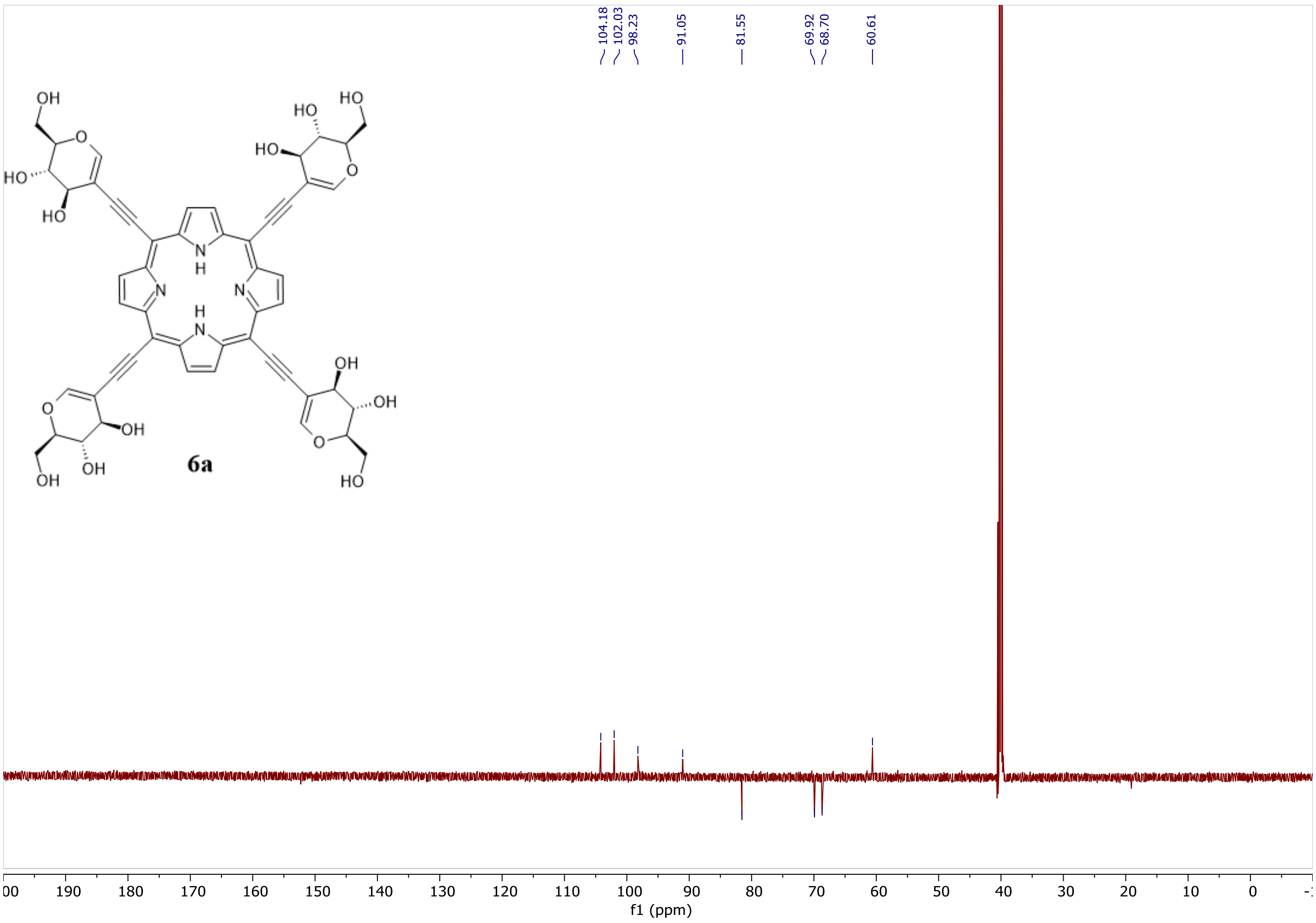

Supplement: Supplementary file 1 [file gg6c00033_si_001.pdf]
